# Design, Structure–Activity Relationships, and Computational Modeling Studies of a Series of α-Helix Biased, Ultra-Short Glucagon-like Peptide-1 Receptor Agonists

**DOI:** 10.3390/molecules30010012

**Published:** 2024-12-24

**Authors:** Jonathon R. Sawyer, Joseph A. Audie, Jon Swanson, David Diller, Solimar Santiago, Valentin K. Gribkoff, Allison Ackerman, Victor J. Hruby, Gianpaolo Gobbo, Michael A. Bellucci, William A. Glauser, Brad L. Pentelute, Tomi K. Sawyer

**Affiliations:** 1 Resolute Bio, 48 Dunham Rd., Suite 5400, Beverly, MA 01915, USA; solimarsantiago01@gmail.com (S.S.); gribkoffv@aol.com (V.K.G.); aackerman26@gmail.com (A.A.); bpentelute@gmail.com (B.L.P.); tomi.sawyer@maestrotherapeutics.com (T.K.S.); 2 Department of Chemistry and Biochemistry, The University of Arizona, 1306 E. University Blvd, Tucson, AZ 85721, USA; hruby@arizona.edu; 3 Peptide Scientia, 8 Nathan Stone Lane, Southborough, MA 01772, USA; 4 Eudoxia Life Sciences, 520 Squire Hill Rd., Cheshire, CT 06410, USA; jon@chemmodeling.com (J.S.); djrdiller@gmail.com (D.D.); 5 XtalPi US, XtalPi Inc., 245 Main Street, Cambridge, MA 02142, USA; gadelio1984@gmail.com (G.G.); michael.bellucci@xtalpi.com (M.A.B.); william.glauser@xtalpi.com (W.A.G.); 6 Maestro Therapeutics, 8 Nathan Stone Lane, Southborough, MA 01772, USA

**Keywords:** glucagon-like peptide-1 (GLP-1), Cα-methylation, *p*-phenyl-phenylalanine (Bip), 2-amino-isobutyric acid (Aib), α-methyl-phenylalanine (α-MePhe), α-methyl-phenylalanine [2-F] (α-MePhe[2-F]), structure–activity relationship (SAR), quantitative structure–activity relationship (QSAR), structure-based design

## Abstract

A systematic structure–activity and computational modeling analysis of a series of glucagon-like peptide-1 receptor (GLP-1R) agonists based upon an ultra-short GLP-1 peptide, H-His-Aib-Glu-Gly-Thr-Phe-Thr-Ser-Asp-Bip-Bip-NH2, was conducted. This highly potent 11-mer peptide led to a deeper understanding of the α-helical bias of strategic α-methylation within the linear parent template as well as optimization of GLP-1R agonist potency by 1000-fold. These data were correlated with previously reported co-structures of both full-length GLP-1 analogs and progenitor N-terminal GLP-1 fragment analogs related to such ultra-short GLP-1R agonist peptides. Furthermore, the development of a quantitative structure–activity relationship (QSAR) model to analyze these findings is described in this study.

## 1. Introduction

**Figure 1:**
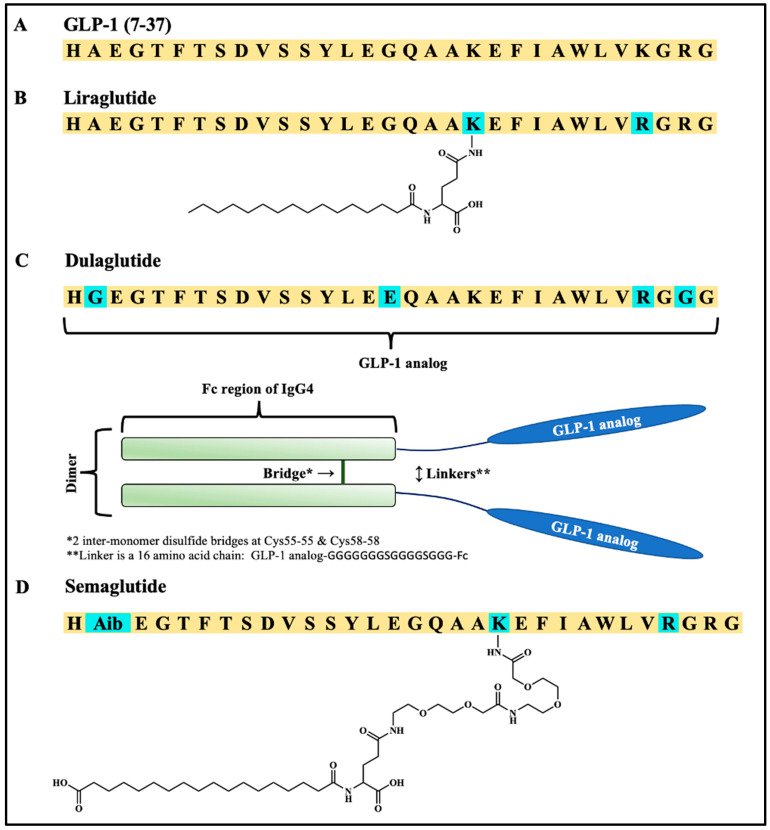
GLP-1 and selected FDA-approved GLP-1 analogs. (**A**) GLP-1 [4], (**B**) liraglutide [5], (**C**) dulaglutide [6], and (**D**) semaglutide [7]. Liraglutide, dulaglutide, and semaglutide are built off the GLP-1 (7–37) fragment. The lipid moieties of liraglutide and semaglutide are linked via γ-L-glutamic acid (γ-Glu). This figure is an approved reproduction taken from the PhD dissertation of Jonathon Sawyer [8].

Glucagon-like peptide-1 (GLP-1) is a member of the incretin family of gastrointestinal hormones that are released upon nutrient intake and modulate glucose-dependent insulin response. During periods of hyperglycemia, GLP-1 receptor activation of the insulin secretory response is increased, and the development of GLP-1 receptor agonists has evolved as a breakthrough for the treatment of type 2 diabetes and, more recently, obesity. Naturally occurring GLP-1 is highly potent as a glucose-dependent insulinotropic peptide; however, it is subject to proteolytic degradation and inactivation by the ubiquitous protease dipeptidyl peptidase-4 (DPP-4) as well as rapid elimination from circulation with a half-life of only a few minutes. GLP-1 analogs (Figure 1) have addressed these liabilities in terms of specific modifications to improve stability and half-life in vivo, including the introduction of albumin-binding moieties as exemplified by the fatty acid conjugate peptides liraglutide and semaglutide, although notable exceptions include GLP-1 analogs such as exenatide [1,2]. The fatty acid conjugation of GLP-1 analogs promotes relatively tight binding to albumin and a low free fraction (e.g., 2% or so) of the peptides in plasma and interstitial fluid, hence establishing a reservoir of albumin-bound GLP-1 with sustained pharmacokinetic and pharmacodynamic properties. In terms of drug delivery, GLP-1 analogs have been administered primarily by subcutaneous administration, although a notable exception has been the recent approval of the first orally bioavailable form of semaglutide. As exemplified by several GLP-1 analogs, substitution of Ala-2 within the GLP-1 sequence by Gly (e.g., albiglutide) or Aib (e.g., liraglutide and semaglutide) increases stability by circumventing proteolytic degradation by DDP-4, which is known to cleave dipeptides from longer peptides or proteins in which Ala (or Pro) exists at position 2. In addition, it is believed that DDP-4 may be compromised sterically by virtue of those GLP-1 analogs having fatty acid modification and their consequent binding to albumin as well as GLP-1 analogs that are covalently conjugated to proteins (e.g., dulaglutide). In the case of liraglutide, the fatty acid modification is achieved by a Lys^26^(γGlu-C16 acid) substitution, and for semaglutide it is achieved with Lys^26^(γGlu-[OEG]_2_-C18 acid). Noteworthily, semaglutide exhibits high potency (6.2 pM) at the human GLP-1 receptor (hGLP-1R) and a sustained preclinical plasma half-life and efficacy that translated to once-weekly dosing in humans [3].

**Figure 2:**
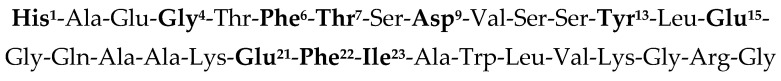
GLP-1 structure highlighting (**bold text**) key residues for its biological activity.

In relation to understanding the structure–activity properties of the N-terminal 11-mer sequence (His^1^-Ala-Glu-Gly-Thr-Phe^6^-Thr-Ser-Asp-Val-Ser^11^~) of GLP-1, Ala-scanning studies have been reported [9,10]. Generally, the overall findings of both groups included the identification of the key residues of the N-terminus of GLP-1, which were His^1^, Gly^4^, Phe^6,^ Thr^7^, and Asp^9^ in terms of Ala replacement, correlating with >10-fold–10,000-fold decreased potency (based on cAMP EC_50_). In terms of molecular recognition, this same N-terminal 11-mer sequence of GLP-1 binds directly into the GLP-1 active site as previously determined by X-ray and cryo-EM studies (vide infra). Furthermore, such structural biology studies have revealed that the C-terminal region also engages the GLP-1 receptor in terms of binding to its extracellular domain. From these same Ala-scanning studies, it was further determined that a few key C-terminal residues contributed significantly to potency (e.g., Tyr^13^, Glu^15^, Glu^21^, Phe^22^, and Ile^23^). The collective results of these early Ala-scanning studies on full-length GLP-1 are summarized in Figure 2.

**Figure 3:**
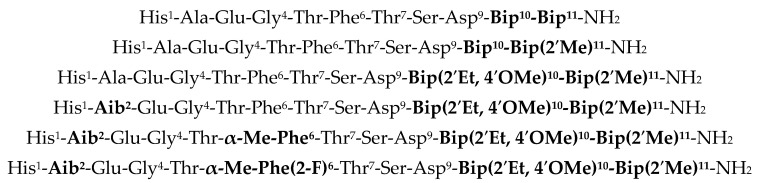
BMS ultra-short GLP-1_1–11_ analogs highlighting (**see bold text**) key sites of modification.

Beyond full-length peptide GLP-1 analogs, noteworthy progress to create ultra-short GLP-1 agonists comprising eleven amino acids was advanced by Bristol Myers Squibb (BMS) [11,12,13] which were shown to exhibit high potency in vitro. A few representative analogs are exemplified in Figure 3, and key modifications correlating with high agonist potency included Aib^2^, α-Me-Phe(2,6-F_2_)^6^, and varying hydrophobic amino acid replacements at Val^10^ and Ser^11^. We were particularly intrigued by this previous breakthrough series of ultra-short GLP-1 peptides within the context of more recent structural biology studies (e.g., X-ray or cryo-EM) of complexes of full-length GLP-1 and such ultra-short GLP-1 analogs with GLP-1 receptor (vide infra) to gain further insight into the comparative molecular recognition properties of these two GLP-1-based peptide modalities with the GLP-1 receptor.

Relative to specific molecules based upon the above series of ultra-short GLP-1 agonist peptides, we focused our structure–activity and quantitative structure–activity relationship (QSAR) computational modeling studies on a systematic analysis of both Ala- and Aib-scanning to differentiate the contribution of side chains and backbone conformational biasing throughout the 11-mer templates as well as perform a deeper exploration of Phe^6^ in terms of side-chain modifications and/or α-methylation. Specifically, three key peptides having Aib^2^ and Bip^10^-Bip^11^ modifications but varying by Phe^6^, Phe(2-F)^6^, or α-Me-Phe(2-F)^6^ (i.e., His-Aib-Glu-Gly-Thr-Phe-Thr-Ser-Asp-Bip-Bip-NH_2_, His-Aib-Glu-Gly-Thr-Phe(2-F)-Thr-Ser-Asp-Bip-Bip-NH_2_, and His-Aib-Glu-Gly-Thr-α-Me-Phe(2-F)-Thr-Ser-Asp-Bip-Bip-NH_2_) provided benchmarks to support these structure–activity and computational modeling studies.

The structural biology of G protein-coupled receptors, including the GLP-1 receptor (GLP-1R), with cognate peptides and analogs thereof as well as small-molecule ligands, provides significant insight into understanding the molecular recognition of both agonist and antagonist modalities [14,15,16,17,18,19]. In the case of GLP-1R, noteworthy progress has been made relative to co-structures with GLP-1, semaglutide, taspoglutide, and exendin peptides [20,21,22,23] as well as an ultra-short truncated peptide agonist (TPA) GLP-1 analog [24] and a small molecule agonist [23,25].

Overall, the above previously reported structure–activity studies on both full-length GLP-1 analogs and the N-terminal 11-mer GLP-1_1–11_ analogs as well as structural biology studies on GLP-1 receptor complexes with peptides and small molecule ligands provided the framework for our current work. Specifically, we wished to perform a systematic study to evaluate the impact of Ala and Aib substitutions within key reference GLP-1_1–11_ analogs along with a deeper analysis of the Phe6 residue in terms of several amino acid replacements. Collectively, what we learned in terms of structure–activity relationships then provided a useful dataset to perform a quantitative structure–activity relationship (QSAR) analysis and exploratory computational modeling studies using the program YASARA Structure (Yet Another Scientific Artificial Reality Application [26]) to interpret our findings.

## 2. Results and Discussion

**Figure 4:**
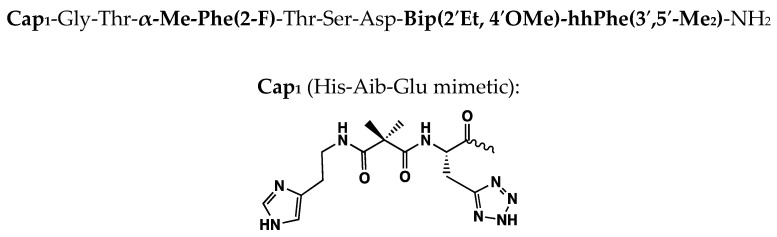
Sosei Heptares GLP-1_1–11_-NH_2_ analog (described as truncated peptide agonist or TPA) was used for X-ray studies in complex with GLP-1R (Jazayeri et al. 2017 [24]).

The early publications [11,12,13] of the BMS ultra-short glucagon-like peptide-1 (GLP-1) analogs inspired this study to revisit the structure–activity relationship (SAR) of these novel GLP-1_1–11_ agonist peptides. We focused on Bip^10^-Bip^11^ at the C-terminus for simplicity (chemistry-wise) but also for the structure–activity studies that are detailed here. Furthermore, the more recent disclosures of GLP-1R X-ray and cryo-EM structures, including apo and complexes with full-length GLP-1 agonist peptides and a C-truncated GLP-1 analog 5 [24] as shown in Figure 4, enabled computational modeling of this series of novel GLP-1 agonist peptides to further understand their SAR as described herein.

### 2.1. Phe^6^ Modifications

We first focused on the Phe^6^ position in this series of ultra-short GLP-1 peptide analogs (Table 1). Fourteen analogs were evaluated to test the original finding by the BMS team [11,12,13] that Phe(2-F)^6^ was superior to Phe^6^ as well as to expand structure–activity analysis relative to other amino acids (e.g., Hph, Bip, Tyr, and Trp) as well as D-Phe^6^. Of those Phe^6^ replacements examined, a majority resulted in decreased potency as GLP-1R agonists. Relative to Phe^6^ itself, phenyl ring substitutions provided intriguing structure–activity relationships. Perhaps most importantly, with respect to earlier studies by the BMS team [11,12,13], the Phe(2-F)^6^ analog (RXL-3000) was the simplest modification that provided superior GLP-1R agonist potency (i.e., approximately fivefold greater versus the parent peptide RXL-100). The Phe(2-F)^6^ observation was further explored relative to various other substituents, including analogs incorporating Phe(2-Cl)^6^, Phe(2-CF_3_)^6^, Phe(2-Me)^6^, and Phe(NO_2_)^6^. Such modifications resulted in 10- to 100-fold decreased GLP-1R agonist potency. Interestingly, the Phe(2,6-F)^6^ analog RXL-3039 was essentially equipotent to RXL-3000, but the penta-fluorinated Phe(2,3,4,5,6-F)^6^ analog RXL-3018 was threefold less potent than RXL-3000. These structure–activity data provide further insights into understanding the structural biology of the related Sosei Heptares GLP-1 analog (Figure 4) with respect to the Phe^6^ residue and its molecular recognition to GLP-1R.

**Figure 5:**
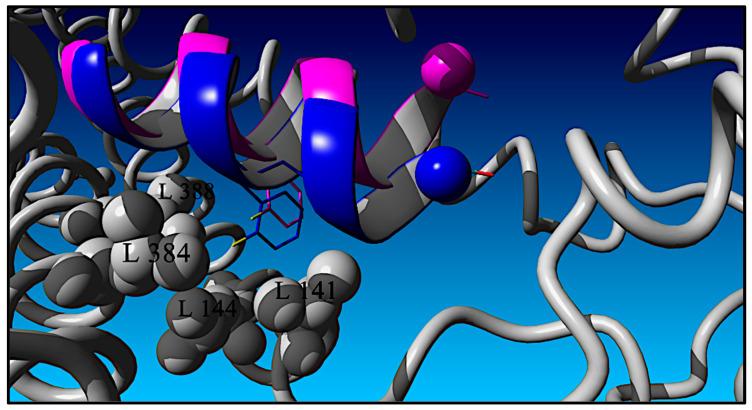
RXL-3000:GLP-1R computational model (RXL-3000 in blue and GLP-1R in grey) based on the Sosei Heptares GLP-1_1–11_-NH_2_ TPA analog complexed with GLP-1R previously determined using cryo-EM methods (5NX2) [24]. Comparative modeling of RXL-3000 based on a previously published [23] GLP-1:GLP-1R structure (6X18) is shown (magenta) to illustrate differences between predicted interactions of the Phe(2-F)^6^ side chain with a hydrophobic pocket (L141, L144, L384, and L388) based on the Sosei Heptares cryo-EM structure. In both cases, the Phe^6^ fluorine atom is colored in yellow. See text for details.

As alluded to previously and discussed in what follows, the data and QSAR analysis presented here validate the RXL-3000:GLP-1R structural binding model that is based on the TPA:GLP-1R cryo-EM (PDB: 5NX2) structure as opposed to the full-length GLP-1 ligand GLP-1:GLP-1R X-ray structure (PDB: 6X18). The TPA-based model of the RXL-3000 ligand in complex with GLP-1R is presented in Figure 5 with GLP-1R as a grey ribbon and the RXL-3000 ligand as a blue ribbon. Figure 5 emphasizes the RXL-3000 residue Phe(2-F)^6^ and its side chain configuration with respect to GLP-1R. The RXL-3000 C-terminal NH_2_ group is depicted as a blue vdw sphere. The RXL-3000 Phe(2-F)^6^ side chain is shown in blue as a stick figure, and its fluorine atom is shown in yellow. GLP-1R side residues in close contact (<4.0 Å) with the RXL-3000 Phe(2-F)^6^ fluorine atom are depicted as vdw spheres and are colored in grey (L141, L144, L384, and L388). For comparison, the GLP-1-based RXL-3000 ligand pose is shown in magenta. In the GLP-1-based model, the N-term of RXL-3000 is offset into the page and is oriented up, while the C-term is inserted slightly deeper in the GLP-1R pocket with respect to RXL-3000 in the TPA-based model. Globally, the two ligands differ by ≈ 2.4 Å in terms of their heavy atoms. The Phe(2-F)^6^ side chain of the TPA-based model is inserted deeper into and packed more tightly in the leucine hydrophobic pocket lined by the GLP-1R residues L141, L144, L384, and L388 than is the Phe(2-F)^6^ side chain of the GLP-1-based model. The distance between the two modeled coordinates of the Phe(2-F)^6^ fluorine atoms is 1.49 Å. Finally, it should be noted that the heavy atom coordinates of the two GLP-1R receptor models are significantly different, with an RMSD of ≈7.6 Å.

### 2.2. Ala-Scanning

For the first time relative to this series of ultra-short GLP-1 peptide analogs, Ala-scanning was performed (Table 2) to investigate the structure–activity relationships of the lead peptide RXL-3000. An analysis of eleven peptides showed that Ala substitution for His^1^, Gly^4^, Phe^6^, Thr^7^, Bip^10^, and Bip^11^ resulted in >100-fold decreased potency, whereas Aib^2^, Glu^3^, Thr^5^, Ser^8^, and Asp^9^ substitutions by Ala resulted in ≤100-fold decreased potency. Most noteworthy was the >1000-fold decreased potency shown by Ala substitutions of Bip^10^ and Bip^11^. Such data indicate the impact of simplification of amino acid side chains to a methyl group (Ala) and that several amino acids (e.g., Glu^3^, Thr^5^, Ser^8^, and Asp^9^) may tolerate further modifications to modulate their hydrophilic character (e.g., H-bonding and charge) and α-helicity propensity (vide infra; Aib-scanning series) to explore the structure–conformation–activity properties of this novel ultra-short GLP-1 peptide modality.

A comparative analysis of the Ala-scanning results on full-length GLP-1 [9] provides further context (see Supplementary Table S5) to those of the ultra-short GLP-1 peptide agonist series (Table 2). In that study, and with a particular focus on the N-terminal 11-mer sequence of GLP-1, it was found that Ala substitutions at His^1^, Gly^4^, and Asp^9^ produced significantly decreased agonist potencies (i.e., >1000-fold), whereas other Ala substitutions, including Phe^6^ (~10-fold), resulted in only a minimal impact on GLP-1R agonist potency. Such findings suggest that these three residues (i.e., His^1^, Gly^4^, and Asp^9^), within the context of full-length GLP-1, play an essential role in achieving a potent (functionally) complex with GLP-1R. Unquestionably, the Phe^6^ modification by Ala in GLP-1 versus the ultra-short GLP-1 analogs is striking, as a >10,000-fold decreased potency was determined for RXL-3038 versus the Phe^6^ parent peptide RXL-100. Furthermore, the hydrophobic Bip^10^ and Bip^11^ residues of RXL-3000 had a similar >1000-fold decreased potency by Ala^10^ and Ala^11^ substitutions (i.e., RXL-3008 and RXL-3009, respectively). In stark contrast, Ala substitutions for Val^10^ and Ser^11^ of GLP-1 had a nominal impact on the resultant GLP-1 analog potency. These data further exemplify the unique molecular recognition and structure–activity relationships of the ultra-short GLP-1 peptide agonists versus full-length GLP-1.

A quantitative comparison was made between the previously reported [9] GLP-1 Ala-scan analog results of Adelhorst et al. and the results for the RXL-3000 Ala-scan analog data presented here (see Supplementary Figure S7). In brief, the pEC_50_ values of the GLP-1 Ala-scan variants and the ten pEC_50_ values of the corresponding RXL-3000 variants (RXL-3001, RXL-3003, RXL-3037, RXL-3004, RXL-3038, RXL-3005, RXL-3006, RXL-3007, RXL-3008, and RXL-3009) did not show a correlation (R^2^ = 0.0098). In contrast, a significant correlation of R^2^ = 0.66 was observed between the previously reported GLP-1 Ala-scan analog pIC_50_ data [9,10]. This suggests that ultra-small RXL-3000 peptides bind to and activate GLP-1R in a different manner than full-length GLP-1 peptides. It can be further hypothesized that RXL-3000 binds and activates GLP-1R in a manner similar to the TPA peptide.

**Figure 6:**
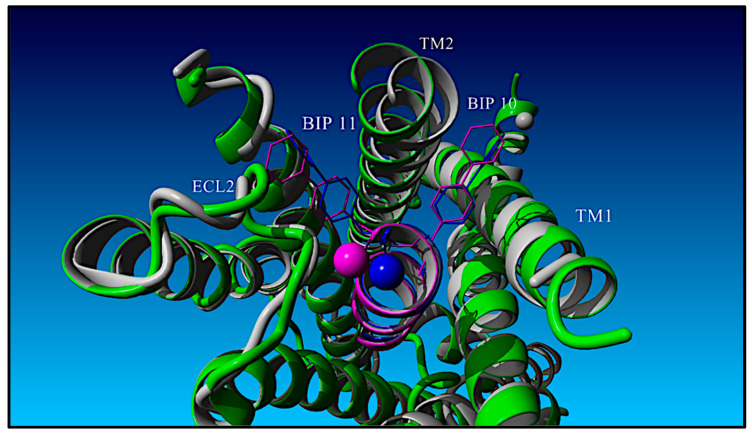
Top-down view of the RXL-3000:GLP-1R computational model based on the 5NX2 TPA-based structure (grey/magenta) versus the RXL-3000:GLP-1R model based on the 6X18 GLP-1-based structure (green/blue). RXL-3000 N-termini are depicted as spheres. In both models, the Bip^10^ side chains are positioned between TM1 and TM2 but with different conformations. Similarly, in both models, the Bip^11^ side chains are positioned close to ECL2 but with distinct conformations.

A top-down view of the TPA GLP-1R structure-based binding model of RXL-3000/GLP-1R is shown Figure 6. The N-terminal extracellular portion of GLP-1R is omitted for clarity. The TPA-based model of RXL-3000 complexed with the GLP-1R 7-transmembrane helix is colored in grey and is depicted coming out of the page, while the GLP-1-based GLP-1R 7-transmembrance helix is colored in green. The C-terminal amide moiety of the TPA-based model of RXL-3000 is represented as a sphere and is colored in blue along with the rest of the peptide, while the GLP-1-based model of the RXL-3000 helix is colored in magenta. The TPA-based model of RXL-3000’s Bip^10^ side chain is positioned between TM2 (residues 174–205) and TM1 (residues 139–169) of GLP-1R, with the first phenyl ring in a “flat” face-up orientation and the second phenyl ring in a “line” side view orientation. By way of comparison, both GLP-1-based Bip^10^ side chain phenyl groups (magenta) are positioned between TM1 and TM2 in the flat orientation. The Bip^11^ side chains in both the TPA-based model and the GLP-1-based model are oriented toward the ECL2 (residues 292–302) of GLP-1R. The TPA-based model of RXL-3000’s Bip^11^ side chain has a flat-line phenyl group configuration, while the GLP-1-based model of RXL-3000’s Bip^11^ side chain has a flat-flat phenyl ring configuration. It is noted that the coordinates of ECL2 for the GLP-1- and TPA-based GLP-1R complexes differ by ≈2.57 Å.

### 2.3. Aib-Scanning

Additionally, for the first time relative to this series of ultra-short GLP-1 peptide analogs, Aib-scanning was performed (Table 3) to inform novel GLP-1 analog optimization. The structure–activity relationships of ten peptides showed that Aib substitution for Gly^4^, Phe^6^, Thr^7^, Ser^8^, Asp^9^, and Bip^10^ resulted in ≥100-fold decreased potency (i.e., RXL-3032, RXL-3028, RXL-3034, RXL-3035, RXL-3036, and RXL-3051, respectively), whereas His^1^, Glu^3^, Gly^4^, and Thr^5^ substitutions by Aib resulted in <100-fold decreased potency. His^1^ replacement by Aib^1^ gave a surprisingly effective GLP-1 agonist (RXL-3030) which was only <10-fold less potent than the parent analog (RXL-101). Glu^3^ replacement by Aib^3^ and Ala^3^ resulted in a similar decrease (i.e., 7- and 14-fold, respectively).

These Aib-scanning structure–activity relationships were further correlated with the previous Ala-scanning data (Table 2) to further understand the impact of α-methylation. Relative to their respective His^1^ parent analog RXL-3000, the Aib^1^ and Ala^1^ substitutions were relatively similar (about 10-fold decreased potency), further implicating that His is non-essential to agonist activity. Aib^2^ versus Ala^2^ substitution resulted in essentially no difference in potency. Glu^3^ replacement by Ala^3^ or Aib^3^ resulted in similarly decreased potencies (about 30- to 60-fold, respectively). Gly^4^ substitution by Aib^4^ (RXL-3032) rescued by about 10-fold an otherwise 2000-fold decrease in potency observed with Ala^4^ substitution (RXL-3037). Thr^5^ replacement by either Aib^5^ or Ala^5^ resulted in a similar 50-fold decrease in potency. Substitution of Phe(2-F)^6^ by Aib^6^ (RXL-3028) rescued by more than 10-fold an otherwise >1000-fold loss of potency observed for Ala^6^ (RXL-3038). Thr^7^ substitution by either Aib^7^ or Ala^7^ resulted in similarly decreased potencies (400- to 600-fold, respectively). Ser^8^ replacement by Aib^8^ (RXL-3034) and Ala^8^ showed that the introduction of α-methylation led to decreased potency (about 10-fold), and in this case, Ala^8^ was superior to Aib^8^, although both were significantly less potent than Ser^8^. Asp^9^ substitution by Aib^9^ (RXL-3036) or Ala^9^ (RXL-3007) gave similar results to those observed for Ser^8^ in terms of Ala^9^ being about fivefold superior to Aib^9^, although both analogs were significantly less potent than Asp^9^. At the C-terminus, quite striking comparative structure–activity relationships were observed for Aib versus Ala substitutions. Specifically, Bip^10^ replacement by Aib^10^ (RXL-3051) or Ala^10^ (RXL-3008) resulted in >10,000-fold decreased potencies. However, Bip^11^ substitution by Aib^11^ (RXL-3052) significantly rescued by more than 100-fold an otherwise 10,000-fold decrease in potency with Ala^11^ (RXL-3009). These data implicate a possible entropically driven impact of α-helicity propensity by the Aib^11^ modification.

The pEC_50_ contribution of Cα-methylation can be estimated from the Aib-scan and Ala-scan pEC_50_ results, summarized in a side-by-side fashion in Table 4. First, the contribution of Cα-methylation at position 6 needs to be calculated using data for the Aib-scanning parent peptide (RXL-101) and the Ala-scanning parent peptide (RXL-3000). This is accomplished by subtracting the pEC_50_ of RXL-3000 (9.92) from the pEC_50_ of RXL-101 (10.89), which results in a favorable Cα-methylation pEC_50_ contribution for position 6 of 0.97. The Cα-methylation-induced change in pEC_50_ (dPE_50_) at other positions can now be calculated according to the general formula:

*dpEC_50_ = pEC_50_ (Aib) − pEC_50_ (Ala) − 0.97* (1)

where pEC_50_ (Aib) refers to the pEC_50_ of the sequence with Aib at a given position, pEC_50_ (Ala) refers to the pEC_50_ of the corresponding sequence with Ala at the same position, and 0.97 is the constant positive contribution of Cα-methylation at position 6. The results for all sequences are presented in Table 4. The results indicate that Cα-methylation is unfavorable at all positions except for position 4 (0.37), 6 (0.97) and 11 (1.11).

**Figure 7:**
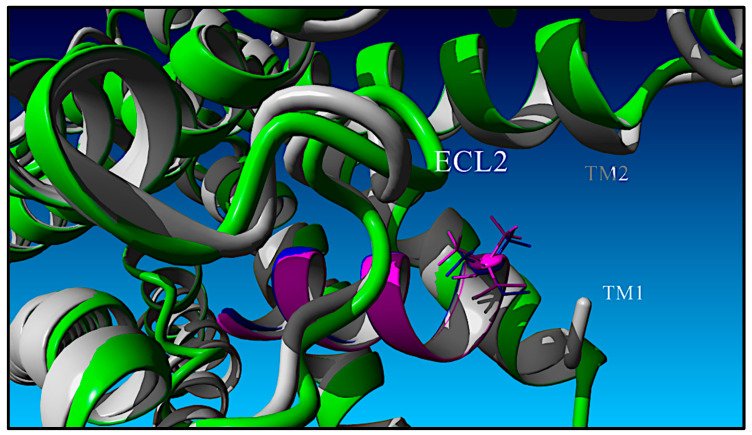
RXL-305 GLP-1R computational model based on the TPA:GLP-1R 5NX2 structure (blue/grey) compared to the RXL-3052:GLP-1R model based on the GLP-1:GLP-1R structure (magenta/green). In both models, Aib fails to make contacts with GLP-1R. This suggests that the increased potency of Aib relative to Ala is mediated by Cα-stabilization of the unbound RXL peptide α-helical binding conformation.

Given the positive contribution of Cα-methylation at position 11, an analysis of Aib^11^ (RXL-3052) was performed using computational modeling, as shown in Figure 7 below. GLP-1R for the GLP-1- and TPA-based models is colored in green and grey, respectively. RXL-3052 is colored in magenta (GLP-1-based) and blue (TPA-based). The Aib coordinates for both models are very close and are within ≈ 0.7 Å. The Aib Cα-carbon at position 11 makes no close contacts with GLP-1R in either model. This suggests that methylation at position 11 may enhance pEC_50_ through stabilization of the active helical binding conformation of the RXL peptide in the unbound state.

### 2.4. Computational Modeling and Comparative Analysis of GLP-1/GLP-R-Based and Non-GLP-1/GLP-1R-Based QSAR Analysis of Phe^6^ Modification, Ala-Scanning, and Aib-Scanning

We used stepwise multiple linear regression (SW-MLR) with multiple sets of a priori determined physics-based interface descriptors and performed calculations using two different RXL/GLP-1R models to develop four QSAR models with the goal of predicting and explaining experimentally obtained pEC_50_ measurements. The use of SW-MLR to develop QSAR models has been described in the literature [27,28,29,30,31,32]. QSAR models were evaluated, and the best models selected were chosen using an algorithmic AIC-based approach and a more nuanced, multi-parameter approach that considers theoretical, physical, experimental, and statistical factors.

A GLP-1-based model of RXL-100 was built using the 6X18 cryo-EM structure of GLP-1:GLP-1R as the template. The RXL-100 model was built by introducing side chain substitutions along positions 1–11 (7–17) of the GLP-1 backbone and optimizing their coordinates. The 5NX2 X-ray structure of GLP-1R bound to a 10-residue truncated peptide agonist (TPA) was selected as the second template on which to construct a non-GLP-1-based or TPA-based model. In this case, RXL-100 was modeled into the GLP-1R binding groove using the X-ray coordinates of the 10-residue TPA agonist backbone as constraints in an MCM optimization. The thirty-one RXL-100, RXL-101, and RXL analogs for which validated EC_50_ data were available (Table 1, Table 2 and Table 3) were generated using computational side chain substitution or Phe^6^ chemical modification followed by minimization of all peptide side chains in the NOVA2 force field.

All four QSAR models were built using structure-based and a priori determined physics-based protein–peptide interface descriptors and SW-MLR analysis. The use of a small number of a priori determined physics-based descriptors helps to guard against “p-fishing” and erroneous term and model selection based on statistical criteria alone. For example, when modeling agonist potencies, assuming that accurate structural models of the active receptor conformation in complex with agonist are available, it can be hypothesized, on physical and theoretical grounds alone and prior to any statistical analysis, that a tighter fit or higher affinity of the agonist with the active conformation will tend to produce higher potencies (pEC_50_). This, in turn, suggests the hypothesis that interface force field terms that tend to favor tight binding (favorable vdw interactions, electrostatic interactions, hydrogen bonding, etc.) will prove useful in a QSAR model for pEC_50_ estimation. By extending this logic and taking note of well-known deficiencies with force fields, the use of more coarse-grained physics-based interface descriptors (hydrophobic, pi–pi, cation–pi, ion–ion, etc.) can be scientifically justified on the grounds that they may capture the essential physics of tight binding while eliminating noise from more numerous and fine-grained but imperfect force field terms. Here, it is instructive to consider the interface vdw contribution, especially as it is mathematically modeled using the 6–12 Lennard-Jones potential. Put simply, it can be argued that the repulsive contribution penalizes small and insignificant structural deviations too much and the use of a total interface contact descriptor or hydrophobic contact descriptor would capture all essential attractive forces while omitting spurious repulsions. Given the known imperfections of fixed partial charge models, a similar line of reasoning can be used to justify the inclusion of coarse-grained descriptors with an electrostatic basis (ion–ion, cation–pi, etc.). Similar arguments can be made as it pertains to solvation phenomena. Ultimately, it seems reasonable to assume that only including terms that enjoy a scientific rationale, followed by statistics-based term selection, will tend to yield more generally explanatory, predictive, and intuitively useful QSAR models that are good candidates for downstream evaluation than reliance on statistical analysis of large numbers of scientifically dubious terms alone.

With the above in mind, the GLP-1-based and TPA physics-based QSAR models were evaluated for parameter-adjusted fit quality (R^2^_adj_), cross-term correlations using inflation variance factors (IVFs < 5.0) [27], overall statistical significance (model p(F-test) ≤ 0.05), and individual regression weight significance (p(*t*-test) ≤ 0.05). Adjusted R^2^ was deemed more useful than R^2^ as it penalizes the addition of independent variables (descriptors) that do not explain the variation in the dependent variable.

The four QSAR models were compared against a single-term NOVA2 binding energy reference model to test for an overly small QSAR model R^2^_adj_ outcome (0.37). To test for an overly high QSAR model R^2^_adj_ outcome, comparison was made with the R^2^_adj_ = 0.62 calculated from experimental pIC_50_ data reported independently by Adelhorst et al. [9] and Gallwitz et al. [10]. The assumption here is that a computational model for predicting experimental results (pEC_50_) should not significantly outperform independent assay experiments for determining related results (pIC_50_) for the same system. Ideally, the experimental control would involve independent pEC_50_ assay measurements with both assays being identical or with one assay being designed to predict the other, but due to what is available in the literature, we must settle for independent pIC_50_ measurements using different assays and without one being specifically developed to predict the other. While these bounds are only approximate, given the differences in the assays, they do provide a useful approximate measure of the quality of the fits likely to be obtained in the current study. Where appropriate, QSAR models were also evaluated for their statistical significance and predictive potential using LOOCV (Q^2^), Y-randomization, and the calculation of R^2^_pred_ and RMSE_pred_ on test sets. Models were also assessed for their physical interpretability.

The results of the 5NX2 TPA-based reference computational model, experimental pIC_50_ model, and four QSAR models are summarized in Table 5. The single-term NOVA2 force field binding energy (BE) TPA model resulted in R^2^_adj_ ≈ 0.37, clear statistical significance (p(f) ≤ 0.05), and Q^2^ ≈ 0.24. The equation relating pEC_50_ to the binding free energy is of obvious physical significance with units of kJ/mol. Recalling that the primary purpose of the one-term NOVA2 model is to establish a lower limit for R^2^_adj_, the upper limit for R^2^_adj_ provided by quantitative analysis of the GLP-1 experimental pIC_50_ data from Adelhorst and Gallwitz [9,10] is ≈0.62 (see Supplementary Table S4 and Figure S8). Hence, a promising QSAR model will exhibit an R^2^_adj_ value above 0.37 and below approximately 0.62. By a similar line of reasoning, a more complicated QSAR model must exhibit a Q^2^ > 0.24, with the important caveat that a Q^2^ value only slightly >0.24 represents a failed model and that a promising model will have a Q^2^ >> 0.24.

SW-MLR using an inclusion/exclusion criterion of α = 0.5 and 12 structure-based interface descriptors (see Formulas (2) and (3)) yielded a statistically significant model (QSAR1) with a single regression weighted term (interaction count) that entails a mediocre R^2^_adj_ = 0.41 and Q^2^ = 0.34. The interaction count term is a sum of all defined favorable weak interactions at the interface and enjoys obvious physical meaning. The same stepwise regression procedure acting on two YASARA model quality descriptors (see Formula (4)—CompMQ and BindMQ) and four 1D and 3D packing descriptors (see Formula (5)—BindPack1D, CompPack1D, BindPack3D, and CompPack3D) resulted in a statistically significant single-term (Bind1Dpack) model (QSAR2) with a poor R^2^_adj_ = 0.31 and Q^2^ = 0.20 that are both below the minimum thresholds values of R^2^_adj_ > 0.39 and Q^2^ > 0.24, respectively, proposed above.

**Figure 8:**
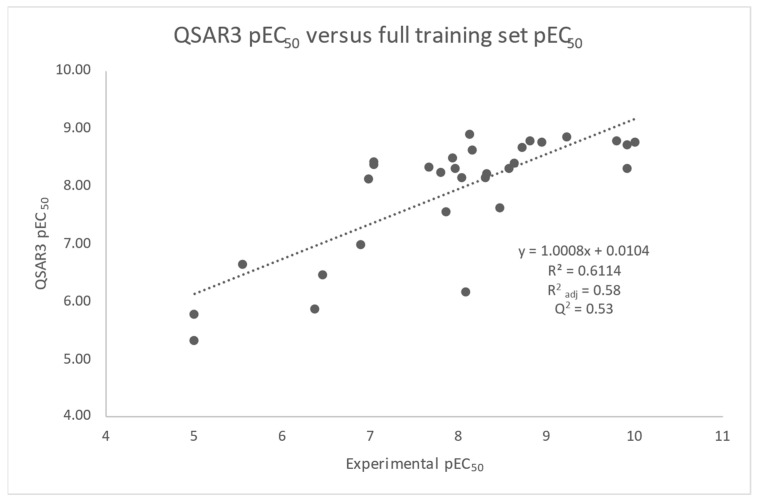
Plot of pEC_50_ values calculated using the QSAR3 model versus the 31 experimentally measured full training set pEC_50_ values.

Stepwise regression analysis on eight NOVA2 force field descriptors (see Formula (7)—BEpot, BEelec, BEvdw, BS, BSelec, BSvdw, BShydro, and BE) resulted in a promising model (QSAR3, Figure 8). The final regression equation relates pEC_50_ as a linear function of the binding potential energy (BEpot), the vdw contribution to the binding solvation free energy (BSvdw), and a constant contribution. The R^2^_adj_ ≈ 0.58 implies a reasonably good fit and that the QSAR model accounts for ≈60% of the variation in the pEC_50_ data. Two weighted terms for 31 pEC_50_ experimental data points exceed the rule of thumb that there should be 10 data points per adjustable term. The overall model p(F-test) ≈ 1.79 × 10^−6^ and the individual BEpot p(*t*-test) ≈ 8.6 × 10^−3^ and BSvdw p(*t*-test) ≈ 0.009 results clearly indicate that the R^2^_adj_ implied by the model and the individual regression weights are highly statistically significant and are not to be expected from a random experiment. The VIFs for both terms (1.36) indicate low collinearity. Importantly, the R^2^_adj_ result of the model is superior to the R^2^_adj_ result of the reference NOVA2 model and lower than the R^2^_adj_ result of the experimental pIC_50_ comparison. As the two model terms are force-field-based, they enjoy obvious physical meaning in the sense that the BEpot term quantifies the sum of attractive and repulsive electrostatic, London-dispersive, and steric clash forces present at a given protein–peptide interface and the BSvdw term captures London-dispersive and steric clash forces between solute and solvent. The sign and magnitude of the BEpot term is physically reasonable and implies that when considered alone, tighter receptor–ligand binding (more negative BEpot) by −10.0 kJ/mol increments will result in a higher pEC_50_ (more potent compound) by +0.26 increments. The sign of the BSvdw contribution implies that when analyzed in isolation, a receptor–peptide complex destabilized by solvent relative to its unbound receptor and peptide by +10 kJ/mol will result in a small increase in pEC_50_ of +0.19. Of course, variation in the predicted pEC_50_ derives from the interplay of both contributions. A reasonable mechanistic interpretation of QSAR3 is that it suggests that peptide binding to the agonist active conformation of GLP-1R is primarily driven by a favorable balance of binding-induced electrostatic and vdw contacts at the interface and the breaking of vdw contacts with the solvent as well as the concomitant and collective cancellation of other intermolecular forces. The model was tested using LOOCV for its predictive potential, resulting in a relatively high Q^2^ ≈ 0.53. Encouragingly, this represents a relatively small drop off from the R^2^_adj_ of 0.58.

Stepwise regression using all 26 descriptors resulted in a promising mixed model (QSAR4). In the final regression equation, pEC_50_ is given as a linear function of the binding potential energy (BEpot), the vdw contribution to the binding solvation free energy (BSvdw), the binding 1D YASARA packing (BindPack1D), and a constant contribution. The R^2^_adj_ ≈ 0.63 implies a good fit and that the QSAR model accounts for 63% of the variation in the pEC_50_ data, which is superior to the reference NOVA2 computational model. This result, however, also suggests that the QSAR4 model R^2^_adj_ is too high, as it exceeds the R^2^_adj_ (0.62) observed between experimental pIC_50_ datasets. Three weighted terms for 31 pEC_50_ experimental data points are compatible with the rule of thumb that there should be 10 data points per weighted term. The model statistics of p(F-test) ≈ 1.22 × 10^−6^, BEpot p(*t*-test) ≈ 1.85 × 10^−3^, BSvdw p(*t*-test) ≈ 0.0017, and BindPack1D p(*t*-test) ≈ 0.04 indicate that the model R^2^_adj_ and individual contributions are statistically significant, although the BindPack1D term is only borderline significant. The VIF for the BEpot terms is low (1.42), but the VIFs for the BSvdw term (3.95) and BindPack1D term (4.1) are borderline high. As the three individual model terms are force-field-based, they enjoy physical meaning, although the mechanistic relationship between BEpot, BSvdw, and BindPack1D is unclear. A change in the BEpot contribution of −10.0 kJ/mol will translate into a +0.29 increase in the pEC_50_. The BSvdw contribution implies that a change of +10 kJ/mol in the BSvdw term will result in a predicted increase in the pEC50 of +0.37. Improved 1D packing at the complex interface by −1000 (lower is better) is expected to translate into a decrease in the pEC_50_ of −0.39. This is a counterintuitive result of dubious physical accuracy. LOOCV analysis resulted in a relatively high but considerably lower Q^2^ ≈ 0.54 with respect to R^2^_adj_ ≈ 0.63.

Despite its slightly lower R^2^_adj_, the two-term NOVA2 TPA-based QSAR3 model arguably represents a better model than the three-term mixed TPA-based QSAR4 model. This is because the QSAR3 model has fewer terms, all terms are NOVA2-based and appear to make intuitive and theoretical physical sense, all terms are highly significant, and neither term exhibits strong collinearity. The same cannot be said about the QSAR4 model. The predictive Q^2^ metrics are essentially the same between the two models, with the QSAR4 model exhibiting a larger drop with respect to its R^2^_adj_ metric. Perhaps most importantly, the QSAR4 model arguably suffers from being over-fit to the pEC_50_ data. An AIC or AIC_c_ approach to model selection would rank the models as follows (best to worst): QSAR4 (−9.81, −7.41), QSAR3 (−6.95, −5.41), QSAR1 (3.18, 4.07), NOVA2 Reference (5.11), and QSAR2 (8.00, 8.89). However, physical evaluation of the regression terms would tend to eliminate QSAR4 and select QSAR3. Taken together, these results suggest that the inclusion of a third term in QSAR4 relative to QSAR3 is not justified and that QSAR3 is the superior model.

Importantly, all 6X18 GLP-1-based QSAR models yielded significantly worse R^2^_adj_ and AIC values when compared to the 5NX2 TPA-based models. The 6X18 GLP-1-based reference model and QSAR1 model, for example, both resulted in R^2^_adj_ = 0.20 and AIC/AIC_c_ ≈ 12.4/13.3. The QSAR2, QSAR3, and QSAR4 models produced R^2^_adj_/AIC/AIC_c_ values of ≈ 0.41/4.0/5.5, 0.41/3.7/5.3, and 0.49/0.048/2.48, respectively. Hence, the 5NX2 TPA-based QSAR models tended to significantly outperform the GLP-1-based models, with the 5NX2 TPA-based QSAR3 model emerging as the best model for RXL-100, RXL-101, and RXL-3000 variant pEC_50_ explanation and prediction. For these and the above reasons, the QSAR3 model was selected as the best available model and worthy of further consideration, and its regression training set and validation results are plotted in Figure 8.

To further evaluate its statistical significance and predictive potential, QSAR3 was evaluated using Y-randomization and three rationally built test sets [33]. The first two test sets were used to test, in a graded fashion, the use of QSAR3 to predict potencies within the training set range or to interpolate potencies. The third test set was used to test the use of QSAR3 to predict potencies outside of the training set range or to extrapolate potencies. The critical evaluation of QSAR3 using Y-randomization and all three test sets allowed for a systematic evaluation of QSAR3 statistical significance and its use to predictively interpolate or extrapolate from training set potencies.

In keeping with previous work, Y-randomization was performed on QSAR3 for 50 trials [34]. The results obtained from the Y-randomized models (average R^2^ = 0.07, average Q^2^ = −0.15, cRp^2^ = 0.58) suggest that the calculated R^2^ and Q^2^ values for the QSAR3 regression model cannot be attributed to random chance (see Supplementary Tables S7–S11). This conclusion is consistent with the preceding analysis and discussion.

The first potency-matched test set was obtained by rank-ordering the original 31-member training set, matching every other member for pEC_50_, and placing one pEC_50_-matched member in the reduced training set and the other one in the new test set. This resulted in a reduced training set of 16 data points and a relatively large test set of 15 closely matched data points, with the training set and test set having essentially identical average pEC_50_ values and standard deviations of 7.90/1.34 and 7.95/1.38, respectively. The re-trained QSAR3 model resulted in an R^2^ = 0.72, with all terms being statistically significant.

Consistent with what has been used by other researchers, the second potency-matched test set was obtained by sorting the original 31-member training compounds according to pEC_50_ and, from each group of five, allocating the first four into the training set and the fifth into the test set [27,29]. This resulted in smaller training and test sets composed of 25 and 6 peptides, respectively. As expected, the means and standard deviations of the pEC_50_ values in the resulting training and tests sets were more different at 7.90/1.37 and 8.21/1.16, respectively. Further, as expected, the re-trained QSAR3 model resulted in an encouraging but lower R^2^ = 0.61, with all terms being statistically significant.

**Figure 9:**
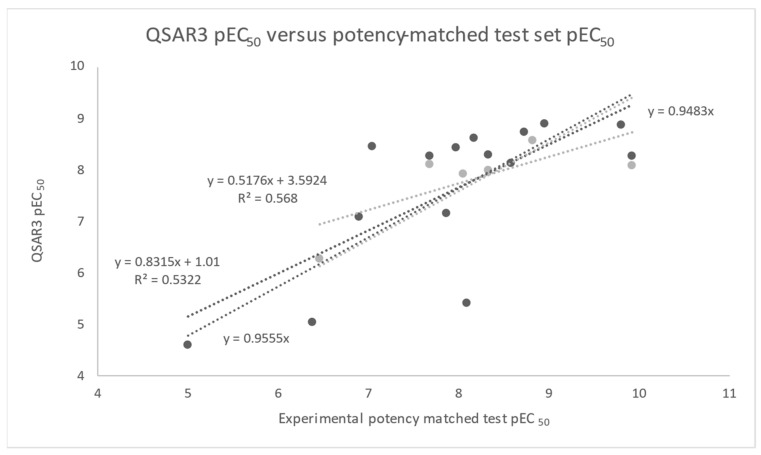
Plot of pEC_50_ values calculated using the retrained QSAR3 models versus the 15 (black) and 6 (grey) experimentally measured potency-matched test sets 1 and 2’s pEC_50_ values, respectively. The graph includes best-fit lines with constants (y = mx + b) and best-fit lines forced through the origin (y = mx). The best-fit lines for test set 1 are y = 0.8315x + 1.01 and y = 0.9555x; the best-fit lines for test set 2 are y = 0.5176x + 3.5924 and y = 0.9483x.

Comparing QSAR3 predictions of test set 1 pEC_50_ values and experimental observations resulted in a best-fit line (y = 0.8315x + 1.01) with a slope of ≈0.83 and an encouraging R^2^_pred_ = 0.53 (Figure 9). Forcing the QSAR3 test set 1 best-fit line through the origin (y = 0.9555x) resulted in a relatively small ≈ 15% deviation of the slope (≈0.96), suggesting that QSAR3 is borderline useful for prediction on the first test set. The same analysis on test set 2 resulted in a best-fit line (y = 0.5176x + 3.5924) with a slope of ≈ 0.52 and an R^2^_pred_ ≈ 0.57 (Figure 9). Encouragingly, the second test set R^2^_pred_ ≈ 0.53–0.57 values bracket the Q^2^ ≈ 0.53. In the case of the second test set, however, forcing the QSAR3 best-fit line through the origin (y = 0.9483x) resulted in a relatively large deviation in the slope, suggesting that QSAR3 is not useful for predictions on the second test set (Figure 9).

To gain more insight into the predictive power of QSAR3, the root mean standard error (RMSE_pred_) was calculated for the first test set predictions (0.75) and second test set predictions (0.52). These RMSE_pred_ results compare reasonably well with the RMSE results (0.5) of the two pIC_50_ experimental assay measurements of Gallwitz et al. and Adelhorst et al. and are within approximately 15% of the range of the target pEC_50_ experimental measurements. Importantly, Alexander, Tropsha, and Winkler have proposed that R^2^_pred_ > 0.60 and RMSE_pred_ within ≈10% of the range of the target property value provide a useful set of criteria by which to judge a QSAR model’s predictive utility in terms of whether it is superior to a standard approach which involves comparing best-fit lines with constant terms versus best-fit lines forced through the origin [35].

Taken together, our first and second test set results suggest that QSAR3, when trained on a broad range of GLP-1 Ala-scan, Aib-scan, and Phe-scan 5NX2 modeled peptide structures and potencies (pEC_50_ 5–10), is a borderline useful model for the prediction or interpolation of similar GLP-1 peptides and potencies.

An even more demanding evaluation of QSAR3 was implemented using a third, potency rank-ordered test set. In this case, QSAR3 was re-trained on the least potent 80% or 60% of data and used to predict the remaining most potent 20% or 40% of data. The R^2^_pred_ result for the 20% test set is disappointing (R^2^_pred_ (20%) = −0.19). While also disappointing, the result for the 40% test set showed a trend in the right direction (R^2^_pred_ (40%) = 0.23). For both the 20% and 40% more potent test set compounds, pEC_50_ predictions showed the right direction in terms of absolute magnitudes and were higher on average by 0.81 and 0.71, respectively. These disappointing/mixed results were not entirely unexpected, as adjusting model regression weights to one dependent variable data range will introduce a bias that will challenge predictions for a poorly represented dependent variable data range. Additionally, deficiencies with the calculated interface descriptors, missing descriptors, and inaccuracies in the underlying 5NX2-based structural models may also account for the predictive failure of QSAR3. Future research could explore these possibilities through the use of molecular dynamics (MD) simulations and ligand-based descriptors to further explore structural space and calculate additional and ensemble-based descriptors. Overall, the results from the potency-ranked test set point to limitations with QSAR3 and indicate that caution should be exercised in using QSAR3 to make predictions that are out of range with respect to its training set.

The final SW-MLR-derived QSAR3 model and its results are summarized as follows:

*pEC_50_ = −0.026BEpot + 0.019BSvdw − 7.26 (QSAR3, R^2^_adj_ = 0.58, Q^2^ = 0.53, Y-randomization = good, R^2^_pred_ = 0.53/0.57, RMSE_pred_ = 0.75/0.52)*

Consistent with the interpretation of the SAR data, the above quantitative results validate our 5NX2-based QSAR3 model for explaining and predicting pEC_50_ values that are represented in a training set and a TPA-based (5NX2) as opposed to a GLP-1-based (6X18) GLP-1R binding and activation mechanism for the RXL-100, RXL-101, and RXL-3000 series peptides presented in Table 1, Table 2, Table 3 and Table 4. The results also indicate that QSAR3 will tend to produce unreliable predictions when applied outside of its training domain. Put more simply, the results validate the TPA-based QSAR3 model for interpolation but fail to validate it for extrapolation.

## 3. Materials and Methods

### 3.1. Peptide Synthesis, Purification, and Characterization

The solid-phase peptide synthesis was achieved by standard methods. Typically, Amphispheres 40 RAM, a 75–150 µM resin (Agilent Technologies), was used to generate peptides as C-terminal carboxamides. Amino acid coupling protocols using HCTU generally included the following four steps: (a) 1st coupling—5 eq of amino acid (0.34M), 10 eq of DIEA (2M), 5 eq of HCTU (0.5M), and 5 eq of 6-Cl-HOBt (0.5M) for 30 min; (b) 2nd coupling—5 eq of amino acid (0.34M), 10 eq of DIEA (2M), 5 eq of HCTU (0.5M), and 5 eq of 6-Cl-HOBt (0.5M), 90 min; (c) one DMF wash between couplings; (d) nine DMF washes after second coupling. Amino acid coupling protocols using HATU generally included the following two steps: (a) single coupling—2 eq of amino acid (0.1M), 4 eq of DIEA (2M), 2 eq of HATU (0.5M), and 5 eq of HOAt (0.5M), 240 min; (b) nine DMF washes after coupling. Amino acid coupling protocols using PyOxim and HATU generally included the following four steps: (a) 1st coupling—5 eq of amino acid (0.34M), 10 eq of DIEA (2M), and 5 eq of PyOxim (0.5M), 120 min; (b) 2nd coupling—5 eq of amino acid (0.34M), 10 eq of DIEA (2M), 5 eq of HCTU (0.5M), and 5 eq of HOAt (0.5M), 120 min; (c) one DMF wash between couplings; (d) nine DMF washes after second coupling. Fmoc deprotection protocols generally included the following three steps: (a) 20% piperidine in DMF, 10 min; (b) 20% piperidine in DMF, 15 min; (c) eight DMF washes. Cleavage of the amino acid side chain protecting groups and the peptide from the resins was typically accomplished by the following five steps: (a) 87.5% TFA, 2.5% anisole, 5% water, and 5% triisopropylsilane, 3–4 h, with 10 mL of cleavage cocktail per 1 g of resin; (b) a modified procedure for sulfur containing amino acids including 85% TFA, 2.5% 3,6-dioxa-1,8-octanedithiol, 2.5% anisole, 5% water, and 5% triisopropylsilane, 3–4 h, with 10 mL of cleavage cocktail per 1 g of resin; (c) TFA evaporation; (d) precipitation with cold diethyl ether (minimum of 10:1, ether/cleavage cocktail) and centrifugation at 3000 rpm for 5 min, and then decanting the ether (this was repeated three times); (e) drying of peptide powder/pellets overnight. Purification by reversed-phase HPLC was achieved by the following four steps: (a) peptide was dissolved; (b) chromatography was performed using a Biotage Selekt instrument and a Biotage Sfär Bio C18 D (Duo, 300 Å, 20 μm): (c) pooling of the desired fractions was performed, followed by freezing and lyophilization; (d) 50% acetonitrile/water was added to dry peptide, followed by re-freezing and re-lyophilization. Analysis of the purified peptides was achieved by the following three steps: (a) a sample of peptide was dissolved and analyzed using an Agilent Infinity II LC/MS; (b) purity (214 and 280 nm absorbance detection) and retention time were analyzed using an Agilent Zorbax 300SB-C18 column (5 µm, 2.1 × 150 mm) and a two-solvent system consisting of A (0.1% TFA in water) and B (0.1% TFA in acetonitrile) with a gradient of 5–65% B over 20 min at 40 °C; (c) MS analysis was conducted using an Agilent Infinity Lab MSD with positive polarity (with a mass detection range of 100 to 1500 Da).

### 3.2. GLP-1 Receptor Agonism Assay

Human embryonic kidney cells (HEK) co-expressing the hGLP1 receptor and CRE-Luciferase construct were used to determine agonist potency in this assay. The cells were thawed briefly at 37 °C, transferred to a sterile tube, and re-suspended in complete media at 37 °C. Cells were centrifuged at 1000 rpm for 5 min and collected; the cells were then re-suspended in an assay buffer consisting of Dulbecco’s phosphate-buffered saline (DPBS, GIBCO) with 500 μM of the phosphodiesterase inhibitor 3-isobutyl-1-methylxanthine (IBMX). The assay medium had 0% serum albumin, as specified in Table 1, Table 2, Table 3 and Table 4. The optimal cell density was determined to be 1000 cells/well. Cells were added to wells in 384-well plates containing appropriate pre-prepared dilutions of compounds (test peptides or reference compound exendin-4), sealed, and incubated with CO_2_ for 30 min. Test peptide solutions were diluted from 10 mM stock solutions to be used in two separate runs (performed in triplicate) from a maximal concentration of 1.0 μM, with 11 concentrations tested for each peptide using serial 1:3 dilution from this maximal concentration. With peptides that were found to be particularly potent agonists, a subsequent assay was run using a maximal concentration of 1.0 nM (11 concentrations, 1:3 dilution from 1 nM). The agonist assay was a homogeneous time-resolved fluorescence (HTRF) assay (Cisbio). Following incubation of the cells for 30 min with test or reference peptides, 5 μL of the cAMP acceptor cAMP-d2, prepared previously as a working solution from frozen stock (1:20 dilution), was added to each well of the assay plate along with 5 μL of anti-cAMP antibody-cryptate working solution (diluted 1:20 from frozen stock). The wells were incubated for 1 h at room temperature, and fluorescence was then read at 665 and 615 nm with an Envision reader with a TRF laser. Data were saved and analyzed using Prism software (GraphPad). Concentration–response analysis was performed using 4-parameter logistic fits of the resulting data, and EC_50_ values were obtained for each test and reference compound.

### 3.3. Computational Structural Modeling and QSAR Model Development, Validation, and Testing

**Figure 10:**
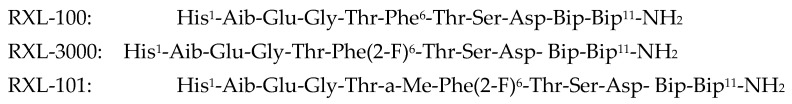
Key parent peptides for structure–activity analysis described in this study.

RXL-100, RXL-3000, and RXL-101 (Figure 10) are key parent peptides for the structure–activity studies described in this study. A systematic series of peptide analogs are detailed in the Results and Discussion sections (vide supra). All of the ultra-short GLP-1 peptide analogs were tested experimentally to determine their EC_50_ values; for computational modeling, pEC_50_ values (pEC_50_ = −log [EC_50_]) were used preferably. The structure and activity relationship (SAR) data were analyzed to identify patterns, including patterns with respect to previously reported [9,10] full-length GLP-1 Ala-scanning results. To facilitate quantitative SAR (QSAR) analysis, two initial structural models of RXL-100 complexed with GLP-1R were constructed. The first model is a GLP-1-based structural model based on full-length GLP-1 complexed with GLP-1R [23], while the second model is based on the related ultra-short GLP-1 truncated peptide agonist (TPA) complexed with GLP-1R [24]. QSAR models were developed using the two RXL-100:GLP-1R input model structures, a common set of a priori determined physics-based peptide–receptor interface descriptors, and stepwise multiple linear regression (SW-MLR) analysis. The QSAR models were critically validated with respect to reference or control models and according to statistical and experimental criteria and evaluated against test sets where appropriate. It was inferred that the best structure-based QSAR model (a) provides a useful basis for continued structure-based design and pEC_50_ prediction and (b) validates the coordinates of the RXL-100:GLP-1R structural model with respect to the competing model.

#### 3.3.1. Peptide/Receptor Structural Modeling Using Sampler and YASARA

Peptide sequences and conformations were built using our own Monte Carlo with minimization (MCM)-based program, Sampler [36]. Peptide conformations were generated from sequences which were constructed from a prebuilt library of amino acids. Each amino acid was built in multiple forms: acylated/amidated, free amino/amidated, and acylated/free carboxy. Each form of each amino acid was parameterized using General Amber Force Field, available in AmberTools20 [37,38]. The appropriate form of each amino acid was chosen (i.e., the free amino/amidated form was used for N-term amino acids, the acylated/free carboxy form was used for C-term amino acids, and the acylated/amidated form was used for the remainder). The initial conformations and full force field parameters were then assembled by joining the individual building blocks using the standard bond length and force field parameters for an amide bond. Peptide conformational searching was performed in dihedral space using multiple copy simulated annealing with a minimization algorithm [36,39]. In brief, in the absence of any constraints, 500 independent runs per peptide were performed. Each run built a stack of 200 low-energy conformations from 10,000 Monte Carlo/minimization steps. Various constraints could be imposed on conformational sampling runs, and the runs could be performed in the presence of a protein binding partner. Implicit solvation effects could also be modeled using the EEF1 implicit solvent model [40].

Peptide sequences and conformations were also built using the modeling and simulation package YASARA [26]. YASARA is a fully featured molecular visualization, modeling, simulation, and analysis software environment that provides access to molecular building and chemical modification functionality, numerous force fields, conformational search and optimization algorithms, molecular dynamics simulation and minimization code, molecular property calculations, and homology modeling and structure validation tools, among other features and functions [41,42].

##### Initial Model Building of the RXL-100:GLP-1R Complex

Two initial RXL-100:GLP-1R structural models were built. One model is referred to as the TPA (truncated peptide agonist) model and is based on the structure of a closely related ultra-short GLP-1 decapeptide complexed with GLP-1R. The second model is referred to as the GLP-1 model and is based on the all-atom structure of GLP-1 bound to GLP-1R.

The all-atom non-GLP-1, or TPA-based, RXL-100 model was built using the 3.7 Å resolution X-ray structure of thermostabilized, full-length GLP-1R in complex with a truncated 10-residue peptide agonist (TPA) as a template (PDB: 5NX2) [24]. All modeling procedures and calculations were performed using the peptide Monte Carlo modeling program Sampler (see above, [36]). The RXL-100 peptide sequence was built as described above. The force field was set to explicitly consider intra-peptide and protein–peptide vdw and electrostatic interactions. Solvation effects were calculated using the EEF1 implicit solvent model. The RXL-100 peptide was subjected to distance-constrained multiple copy Monte Carlo minimization as described above and according to the 5NX2 atomic backbone coordinates of the TPA ligand.

The all-atom GLP-1-based model of RXL-100 in complex with GLP-1R was built using the template provided by the 6X18 cryo-EM GLP-1:GLP-1R structure with a resolution of 2.1 Å [23]. All modeling procedures and calculations were performed using YASARA (see above, [42]). First, 6X18 was downloaded from the PDB and all water molecules and proteins other than GLP-1 and GLP-1R were removed. Secondly, the 6X18 GLP-1 peptide was truncated to residues 7–17 (1–11). Thirdly, the RXL-100 N-terminal His^1^ was protonated, and the C-terminal Bip^11^ was made into a carboxamide. Fourthly, hydrogens were added, and other standard clean-up operations were performed on the RXL-100:GLP-1R complex using the YASARA Clean algorithm. Fifthly, using the YASARA Swap tool, amino acid residues were systematically substituted along the truncated GLP-1 peptide backbone to reflect the RXL-100 sequence. Each residue substitution was immediately followed by optimization of its side chain conformation using the YASARA Optimization algorithm (all available methods selected) [43]. After introducing all individual residue substitutions/optimizations, all peptide ligand side chains were subject to semi-flexible energy minimization using the YASARA Energy Minimization Experiment algorithm with default parameters [44]. All non-side chain atoms were held fixed during energy minimization.

##### Phe, Ala-Scan, and Aib-Scan Chemical Modifications and Structural Modeling

Phe, Ala, and Aib modifications for the GLP-1-based and TPA-based RXL-100:GLP-1R models were performed using the YASARA molecular modeling and simulation software package. Phe^6^ modifications (Table 1) were performed by clicking on the relevant Phe^6^ benzyl ring atom and using the YASARA Swap tool to introduce the relevant chemical group modification. This was followed by flexible side chain minimization using the YASARA Energy Minimization Experiment with default parameters, with all other atoms being held fixed. The Ala-scan analogs (Table 2) were modeled into the RXL-100 peptide using simple side chain truncation. The Aib-scan analogs (Table 3) were modeled by swapping the Cα-hydrogen atom with an α-carbon atom and adding hydrogens. This was followed by Aib residue side chain minimization (using the YASARA Energy Minimization Experiment algorithm with default settings), with other atoms held fixed to their starting coordinates.

##### Physics-Based QSAR Descriptors and Calculations

QSAR models were developed using stepwise multiple linear regression (SW-MLR) analysis acting on a priori determined physics-based descriptors, as implemented in Real Statistics Using Excel (Zaiontz). The dependent or response variable used in the SW-MLR was pEC_50_. Independent or predictor variables were calculated using various receptor–peptide force-field-based descriptors and physics-based interface descriptors. The descriptors were put into sets based on the type of descriptor. All calculations were performed using in-house YASARA macro scripts.

The first set of calculated physics-based interface descriptors included counts and energies of interface hydrogen bonds, hydrophobic bonds, cation–pi bonds, π–π bonds, and ion–ion bonds. Simple derived scoring functions of total favorable interface interaction counts and energies were also calculated (see Formulas (2) and (3) below). All calculated energies are reported in the unit of kJ/mol. A total of 12 descriptors made up the set.

*InteractionCount = H-bond count + Hydrophobic count +* *Cation-π count + Pi-Pi count + Ion-Ion count* (2)

*InteractionEnergy = H-bond energy + Hydrophobic energy +*

*Cation-π energy +Pi-Pi energy + Ion-Ion energy* (3)

The second set of interface descriptors included the YASARA2 force-field-based model quality (MQ) score, 1D YASARA2 force-field-based packing score, and 3D YASARA2 force-field-based packing score [43]. All three YASARA scores were calculated for receptor–peptide complexes (CompMQ, CompPack1D, and CompPack3D). Receptor–peptide binding scores were also calculated by calculating the model quality, 1D packing, or 3D packing score for a complex and subtracting it from the corresponding scores calculated for the separated peptide and protein (see Formulas (4)–(6) below). BindMQ is unitless, while BindPack1D and BinbdPack3D are reported in the unit of kJ/mol. A total of 6 complex and binding descriptors comprised the set.

*BindMQ = CompMQ − ReceptorMQ − PeptideMQ* (4)

*BindPack1D = CompPack1D − ReceptorPack1D − PeptidePack1D* (5)

*BindPack3D = CompPack3D − ReceptorPack3D − PeptidePack3D* (6)

The third set of interface descriptors was calculated based on the YASARA NOVA2 force field and an implicit solvation model [44]. A similar force field/solvent model combination was used successfully by our group in a previous modeling study [36]. To speed up calculations, the solvation energy was calculated using the boundary element method implemented in YASARA [45]. The boundary between the solvent (dielectric constant 78) and solute (dielectric constant 1) was formed by the latter’s molecular surface, constructed with a solvent probe radius of 1.4 Å and the following radii for the solute elements: polar hydrogens, 0.32 Å; other hydrogens, 1.017 Å; carbon, 1.8 Å; oxygen, 1.344 Å; nitrogen, 1.14 Å; sulfur, 2.0 Å. The solute charges were assigned based on the NOVA2 force field [43,44] using GAFF2/AM1BCC [38,46] for the ligands. Seven NOVA2 force field binding/implicit solvation terms (binding potential energy (BEpot), binding coulombic or electrostatic energy (BEelec), binding van der Waals energy (BEvdw), binding total solvation free energy (BS), binding electrostatic or coulombic solvation free energy (BSelec), binding van der Waals solvation free energy (BSvdw), and binding hydrophobic solvation free energy (BShydro)) were calculated for receptor–peptide structures. The overall NOVA2 binding free energy (BE) was also calculated (see Formula (7) below). The BE and all NOVA2 force field and implicit solvent model terms are in kJ/mol. The single-term NOVA2 BE model served as the reference or benchmark model for all the SW-MLR-derived QSAR models.

*BE = BEpot − BS − BShydro* (7)

##### QSAR Model Training

All QSAR models were trained by SW-MLR using a representative set of 31 peptide sequences for which validated EC_50_ (pEC_50_) results were available. The 31 peptides are among those presented in Table 1, Table 2 and Table 3. Excluded peptides are indicated with a star (*). All Ala-scan and Aib-scan peptide analogs were included, and 9 Phe-modified peptides were included. As mentioned above, the quantitative structure activity (QSAR) models for predicting and explaining pEC_50_ results (dependent variable) were trained using SW-MLR acting on 26 physics-based interface descriptors (independent variables) that were also divided into sets based on the descriptor type. The stepwise α descriptor inclusion or exclusion in the MLR model was set to 0.05. Three distinct QSAR models were built based on the interface descriptor type: (a) NOVA2 force field descriptors (8), (b) YASARA interface contact descriptors (12), and (c) YASARA quality/packing descriptors (6). A fourth mixed QSAR model based on all 26 descriptor types was also built. The single-term NOVA2 force-field-based binding energy model served as the control or reference model.

##### QSAR Model Validation and Selection Using Two Approaches

Two model validation and selection protocols were applied. In the first protocol, the models were evaluated for parameter-adjusted goodness-of-fit (R^2^_adj_), overall statistical significance (overall model f-tests (p(f-test))), individual regression coefficient statistical significance (regression term *t*-tests (p(*t*-test))), and inter-regression term correlations using variance inflation factors (VIFs). The models were also evaluated for having an overly small R^2^_adj_ effect (underfitting) in comparison to the single-term NOVA2 force field reference model. The models were also evaluated for having an overly large R^2^_adj_ effect (overfitting) by comparing QSAR model regression R^2^_adj_ effects with the R^2^_adj_ effect of experimental pIC_50_ results for GLP-1:GLP-1R (i.e., R^2^_adj_ of experimental GLP-1 pIC_50_ values reported by Gallwitz and co-workers versus experimental pIC_50_ values reported for GLP-1 by Adelhorst and co-workers). Model terms were also evaluated for their physical interpretability (e.g., it makes physical or theoretical sense for a term to have a negative regression weight). Finally, the models were evaluated for their predictive potential by calculating each model’s leave-one-out cross-validation R^2^ (Q^2^). To converge on the best validated model, an inductive argument must be made that considers all these factors.

In the second model validation and selection protocol, a more strictly algorithmic approach was used. In brief, the QSAR models were ranked by their Akaike Information Criterion (AIC) and corrected Akaike Information Criterion (AIC_c_). The model with the best (lowest) AIC/AIC_c_ score was then evaluated for the physical meaningfulness of its individual terms. If the model with the best AIC/AIC_c_ score also passed the physical interpretability test, it was selected as the best model. If the physical interpretability test was not passed, the model with the next best AIC/AIC_c_ score was assessed. The procedure was continued until the best validated model was identified (or until all models failed).

##### QSAR Model Testing

The best QSAR model (or models) was evaluated against three test sets. The first potency-sorted test set was built by first re-ordering the 31-sequence training set according to pEC_50_ from lowest to highest and then matching every other sequence and pEC_50_ and placing the matched pEC_50_ pairs into the training set or test set. This resulted in a 16-member training set and 15-member potency-matched pEC_50_ test set. The second potency-sorted and matched test set was also obtained by sorting the peptides by ascending pEC50 values. For the second test set, however, every fifth peptide was allocated to the test set, which resulted in a total of 6 test set peptides. The relevant QSAR model(s) were then re-trained by MLR on the 16-member or 25-member training set and used to make predictions for the 15-member and 6-member test sets. The third test set was built by re-ordering the original 31-sequence training set according to pEC50 from lowest (lowest pEC50) to highest (highest pEC50). Relevant QSAR models were then re-trained using MLR on either 60% or 80% of the least potent sequences and used to predict pEC50 values for the remaining 40% or 20% most potent sequences. Where appropriate, test results were assessed by 50 trials of Y-randomization (randomly change the dependent pEC_50_ variables (Y) while holding constant the independent descriptor variables (Xs)) and by comparing the experimentally measured test set pEC_50_ values with the predicted pEC_50_ values (R^2^_pred_, RMSE_pred_) as well as by comparing differences between the average calculated potencies for the least and most potent peptides (average pEC_50_ [more potent test set] − average pEC_50_ [less potent training set]).

## 4. Conclusions and Future Directions

In this investigation, we have recapitulated key findings of the pioneering work of BMS researchers who first showed that ultra-short GLP-1 peptide agonists having a similar potency to native GLP-1 were achievable. Their work highlighted modifications of Aib^2^ and α-Me-Phe(2-F)^6^ and varying hydrophobic amino acid modifications at residues 10 and 11 of an 11-mer template. Furthermore, we have expanded the structure–conformation–activity relationship understanding of this linear peptide series by systematic analysis of Phe^6^ as well as both Ala- and Aib-scanning to determine the contribution of side chains throughout the 11-mer peptide template. The noteworthy findings were that His^1^ may be replaced by either Ala^1^ or Aib^1^ and that relative to our benchmark peptides, having Bip^10^-Bip^11^ modifications with Aib^11^ yielded a low nM GLP-1R agonist.

A comparative profile of each ultra-short GLP-1 analog within the Ala-, Aib-, and Phe^6^-scanning series was prepared using a colorimetric potency analysis (Table 6). This profiling clearly shows the significance in terms of GLP-1R agonist potency contributions at positions 6 (e.g., α-Me-Phe(2-F)), 10 (e.g., Bip), and 11 (e.g., Bip). For example, Cα-methylation of Phe at position 6 is a key contributor to potency improvement, as exemplified by comparative analysis with Phe, Phe(2-F), and α-Me-Phe(2-F). A comparison of both the Ala-scan and Aib-scan at both Bips at positions 10 and 11 showed that Aib was considerably better tolerated at position 11. Given that there was a significant loss in terms of potency at position 11 as seen in the Ala-scanning series, the discrepancy in terms of potency regarding the Ala and Aib substitutions suggests that the enhanced helicity induced by Aib^11^ (RXL-3052) alleviated the potency impact caused by a loss of enthalpic interactions of Bip^11^ at the GLP-1R.

Our QSAR modeling efforts and results must be assessed against the background of two sets of experimental pIC_50_ data that exhibit limited correlation, which limits the expected QSAR model correlation. Additionally, while much QSAR work has been focused on small molecules, our work focused on large peptide ligands. Given this context, our results indicate that stepwise multiple linear regression and QSAR modeling using a relatively small set of physics-based descriptors and a relatively small training set of pEC_50_ measurements can be used to train and derive a good explanatory model. However, obtaining good predictive results on test sets has proven to be more problematic, with borderline success in predictive interpolation and failure in predictive extrapolation. This indicates that when it comes to prediction, other factors need to be considered that are not in the model. Future work will focus on identifying and incorporating those missing factors into an improved model. Finally, given the comparative and consistent superiority of our 5NX2 (small peptide ligand)-based QSAR models relative to our 6X18 (large GLP-1 ligand)-based QSAR models, our results provide evidence that the ultra-short GLP-1 analogs bind GLP-1R in a manner similar to the small peptide ligand of the 5NX2 structure.

Our computational modeling studies, based upon GLP-1R structural studies related to complexes with both full-length GLP-1 [23] and an ultra-short GLP-1 TPA analog [24] as well as comparative structure–activity analysis of Ala-scanning studies previously reported [9,10] versus data described here, indicate that α-helicity is a dominant conformational feature of such GLP-1 peptide agonists and that residues 2, 6, and 11 may especially be correlated with α-helical propensity. Further, the study found that hydrophobic amino acid substitutions of two amino acids at residues 10 and 11 within the ultra-short GLP-1 peptide agonist template (e.g., Bip^10^-Bip^11^) achieve the equivalent agonist potency of full-length GLP-1. This suggests that even for this class of G protein-coupled receptors, in which the extended C-terminal sequence contributes to added enthalpy of binding to the extracellular domain of the receptor, there may be opportunistic design strategies to identify and further optimize ultra-short peptide agonists as well as antagonists which may become next-generation therapeutic modalities. Of course, such work will require successful achievement of pharmacokinetic and drug delivery properties to advance such ultra-short peptide drugs, and this will be the focus of our future efforts to integrate more sophisticated computational modeling methods along with innovative synthetic chemistry.

Comparative structure-based computational modeling and physics-based QSAR analyses were undertaken to explain and predict pEC_50_ results. To this end, computational models of GLP-1R in complex with the tested peptides were built using a 5NX2 GLP-1R/TPA template and a 6X18 GLP-1R/GLP-1 template. A priori determined physics-based receptor–peptide interface descriptors were calculated, and SW-MLR was used to train several QSAR models. The QSAR models were critically compared and evaluated for underfitting (relative to the R^2^_adj_ single-term binding energy control model) and overfitting (relative to the R^2^_adj_ observed between independently collected experimental pIC_50_ measurements) and according to physical and statistical criteria, including LOOCV (Q^2^). In all cases, the 5NX2-based models outperformed the 6X18-based models, with the 5NX2-based QSAR3 model emerging as the best validated model. As such, QSAR3 was subjected to three rounds of increasingly demanding testing against two potency-matched test sets and one potency-ranked test set. The results for the first two potency-matched test sets proved to be encouraging, while the results for the potency-ranked testing proved to be disappointing, if not unexpected. Overall, the results suggest that the 5NX2-based QSAR3 model provides a borderline useful and physically intuitive model for quantitatively explaining and predicting the pEC_50_ consequences for a range of chemical modifications to short GLP-1R peptide agonists when it is applied within the ranges of its training data. That is to say, the TPA-based QSAR3 test set results tend to validate QSAR3 in predictive interpolation. Caution, however, must be exercised when QSAR3 is extrapolated beyond its training set. That is to say, our results failed to validate QSAR3 in extrapolation. Importantly, the results presented here validate a TPA- or 5NX2-based binding mode for our peptides as being more probable than a GLP-1- or 6X18-based binding mode. Our results also call attention to the role Cα-methylation may play in enhancing GLP-1R potency through the stabilization of unbound helical binding conformations. Hence, to better account for the effects of Cα-methylation and other chemical modifications, future work will focus on the incorporation of ligand-based descriptors in QSAR model training and testing. Future work will also focus on the use of receptor/peptide and peptide molecular dynamics simulations to generate ensemble-based descriptors for QSAR model training and testing.

## Figures and Tables

**Table 1:** Structure–activity relationships of GLP_1–11_ analogs: Phe6 modifications (0% HSA).

Peptide	Peptide Structure (N-Terminus, Amino Acid Sequence, C-Terminus)			cAMP EC_50_, nM
GLP-1	H	His^1^-Ala-Glu-Gly-Thr-Phe^6^-Thr-Ser-Asp-Val-Ser^11^	Residues 12–30	***
RXL-100	H	His-Aib-Glu-Gly-Thr-Phe-Thr-Ser-Asp-Bip-Bip	NH_2_	0.5
RXL-3000	H	His-Aib-Glu-Gly-Thr**-Phe(2-F)-**Thr-Ser-Asp-Bip-Bip	NH_2_	0.1
RXL-3010	H	His-Aib-Glu-Gly-Thr-**Phe(2-Br)**-Thr-Ser-Asp-Bip-Bip	NH_2_	3.85
RXL-3011	H	His-Aib-Glu-Gly-Thr-**Phe(2-Cl**)-Thr-Ser-Asp-Bip-Bip	NH_2_	0.9
RXL-3012	H	His-Aib-Glu-Gly-Thr-**Phe(2-CF_3_)-**Thr-Ser-Asp-Bip-Bip	NH_2_	13.9
RXL-3013	H	His-Aib-Glu-Gly-Thr-**Phe(2-CH_3_)-**Thr-Ser-Asp-Bip-Bip	NH_2_	2.1
RXL-3014	H	His-Aib-Glu-Gly-Thr-**Phe(2-NO_2_)**-Thr-Ser-Asp-Bip-Bip	NH_2_	1.1
RXL-3015	H	His-Aib-Glu-Gly-Thr-**Phe(2-CN)**-Thr-Ser-Asp-Bip-Bip	NH_2_	7.7
RXL-3016	H	His-Aib-Glu-Gly-Thr-**Phe(3-CF_3_)**-Thr-Ser-Asp-Bip-Bip	NH_2_	13.9
RXL-3039	H	His**-**Aib-Glu-Gly-Thr-**Phe(2,6-F)**-Thr-Ser-Asp-Bip-Bip	NH_2_	0.16
RXL-3017	H	His**-**Aib-Glu-Gly-Thr-**Phe(3,4,5-F)**-Thr-Ser-Asp-Bip-Bip	NH_2_	7.2
RXL-3018	H	His**-**Aib-Glu-Gly-Thr-**Phe(2,3,4,5,6-F)**-Thr-Ser-Asp-Bip-Bip	NH_2_	0.3
RXL-3019	H	His-Aib-Glu-Gly-Thr**-Hph-**Thr-Ser-Asp-Bip-Bip	NH_2_	16.0
RXL-3024	H	His-Aib-Glu-Gly-Thr**-Bip-**Thr-Ser-Asp-Bip-Bip	NH_2_	10.8
RXL-3021	H	His-Aib-Glu-Gly-Thr**-Tyr-**Thr-Ser-Asp-Bip-Bip	NH_2_	3.0
RXL-3023	H	His-Aib-Glu-Gly-Thr**-Trp-**Thr-Ser-Asp-Bip-Bip	NH_2_	13.8
RXL-3022	H	His-Aib-Glu-Gly-Thr**-D-Phe-**Thr-Ser-Asp-Bip-Bip	NH_2_	23.2

The EC_50_ and E_MAX_ values for all peptides were determined at the GLP-1 receptor using an HTRF cAMP assay. The average for both values is provided based on two independent experiments in triplicate titrations with the exception for RXL-3039 which was only tested one time. All EC_50_ values are expressed in nanomolar (nM) concentrations. An EC_50_ > 1000 nM indicates that it was out of range. See Supplementary Figures S1 and S2 for the original dose–response curves.

**Table 2:** Structure–activity relationships of GLP_1–11_ analogs: Ala-scanning (0% HSA).

Peptide	Peptide Structure (N-Terminus, Amino Acid Sequence, C-Terminus)			cAMP EC_50_, nM
GLP-1	H	His^1^-Ala-Glu-Gly-Thr-Phe^6^-Thr-Ser-Asp-Val-Ser^11^	Residues 12–30	***
RXL-100	H	His-**Aib**-Glu-Gly-Thr-Phe-Thr-Ser-Asp-**Bip-Bip**	NH_2_	0.5
RXL-3000	H	His-Aib-Glu-Gly-Thr**-Phe(2-F)-**Thr-Ser-Asp-Bip-Bip	NH_2_	0.1
RXL-3001	H	**Ala**-Aib-Glu-Gly-Thr-Phe(2-F)-Thr-Ser-Asp-Bip-Bip	NH_2_	21.9
RXL-3002	H	His-**Ala**-Glu-Gly-Thr-Phe(2-F)-Thr-Ser-Asp-Bip-Bip	NH_2_	0.2
RXL-3003	H	His-Aib-**Ala**-Gly-Thr-Phe(2-F)-Thr-Ser-Asp-Bip-Bip	NH_2_	7.9
RXL-3037	H	His-Aib**-**Glu-**Ala**-Thr-Phe(2F)-Thr-Ser-Asp-Bip-Bip	NH_2_	210.0
RXL-3004	H	His-Aib-Glu-Gly-**Ala**-Phe(2-F)-Thr-Ser-Asp-Bip-Bip	NH_2_	7.7
RXL-3038	H	His-Aib-Glu-Gly-Thr-**Ala**-Thr-Ser-Asp-Bip-Bip	NH_2_	>1000
RXL-3005	H	His-Aib-Glu-Gly-Thr-Phe(2-F)-**Ala**-Ser-Asp-Bip-Bip	NH_2_	47.5
RXL-3006	H	His-Aib-Glu-Gly-Thr-Phe(2-F)-Thr-**Ala**-Asp-Bip-Bip	NH_2_	8.5
RXL-3007	H	His-Aib-Glu-Gly-Thr-Phe(2-F)-Thr-Ser-**Ala**-Bip-Bip	NH_2_	14.0
RXL-3008	H	His-Aib-Glu-Gly-Thr-Phe(2-F)-Thr-Ser-Asp-**Ala**-Bip	NH_2_	>1000
RXL-3009	H	His-Aib-Glu-Gly-Thr-Phe(2-F)-Thr-Ser-Asp-Bip-**Ala**	NH_2_	>1000

The EC_50_ and E_MAX_ values for all peptides were determined at the GLP-1 receptor using an HTRF cAMP assay. The average for both values is provided based on two independent experiments in triplicate titrations. All EC_50_ values are expressed in nanomolar (nM) concentrations; >1000 nM indicates that the EC_50_ was out of range. See Supplementary Figures S3 and S4 for the original dose–response curves.

**Table 3:** Structure–activity relationships of GLP_1–11_ analogs: Aib-scanning (0% HSA).

Peptide	Peptide Structure (N-Terminus, Amino Acid Sequence, C-Terminus)			cAMP EC_50_, nM
GLP-1	H	His^1^-Ala-Glu-Gly-Thr-Phe^6^-Thr-Ser-Asp-Val-Ser^11^	Residues 12–30	***
RXL-100	H	His-**Aib**-Glu-Gly-Thr-Phe-Thr-Ser-Asp-**Bip-Bip**	NH_2_	0.5
RXL-3000	H	His-Aib-Glu-Gly-Thr-**Phe(2-F)**-Thr-Ser-Asp-Bip-Bip	NH_2_	0.1
RXL-101	H	His-Aib-Glu-Gly-Thr-**a-Me-Phe(2-F)**-Thr-Ser-Asp-Bip-Bip	NH_2_	0.01
RXL-3030	H	**Aib-**Aib-Glu-Gly-Thr-**a-Me-Phe(2-F)**-Thr-Ser-Asp-Bip-Bip	NH_2_	1.3
RXL-3031	H	His-Aib-**Aib**-Gly-Thr-**a-Me-Phe(2-F)**-Thr-Ser-Asp-Bip-Bip	NH_2_	4.0
RXL-3032	H	His-Aib-Glu-**Aib**-Thr-**a-Me-Phe(2-F)**-Thr-Ser-Asp-Bip-Bip	NH_2_	14.2
RXL-3033	H	His-Aib-Glu-Gly-**Aib-a-Me-Phe(2-F)**-Thr-Ser-Asp-Bip-Bip	NH_2_	6.9
RXL-3028	H	His-Aib-Glu-Gly-Thr-**Aib**-Thr-Ser-Asp-Bip-Bip	NH_2_	161.0
RXL-3034	H	His-Aib-Glu-Gly-Thr-**a-Me-Phe(2-F)-Aib**-Ser-Asp-Bip-Bip	NH_2_	80.2
RXL-3035	H	His-Aib-Glu-Gly-Thr-**a-Me-Phe(2-F)**-Thr-**Aib-**Asp-Bip-Bip	NH_2_	111.0
RXL-3036	H	His-Aib-Glu-Gly-Thr-**a-Me-Phe(2-F)**-Thr-Ser-**Aib**-Bip-Bip	NH_2_	67.5
RXL-3051	H	His-Aib-Glu-Gly-Thr-**a-Me-Phe(2-F)**-Thr-Ser-Asp-**Aib**-Bip	NH_2_	>1000
RXL-3052	H	His-Aib-Glu-Gly-Thr-**a-Me-Phe(2-F)**-Thr-Ser-Asp-Bip-**Aib**	NH_2_	7.4

The EC_50_ and E_MAX_ values for all peptides were determined at the GLP-1 receptor using an HTRF cAMP assay. The average for both values is provided based on two independent experiments in triplicate titrations. All EC_50_ values are expressed in nanomolar (nM) concentrations; >1000 nM indicates that the EC_50_ was out of range. See Supplementary Figures S5 and S6 for the original dose–response curves.

**Table 4:** Structure–activity relationships of GLP_1–11_ analogs: the contribution of Cα-methylation derived from Aib/Ala-scanning (0% HSA) results.

Peptide	Peptide Structure	pEC_50_	Peptide	Peptide Structure	pEC_50_	dpEC_50_
RXL-101	His-Aib-Glu-Gly-Thr-**α-Me-Phe(2-F)**-Thr-Ser-Asp-Bip-Bip	10.89	RXL-3000	His-Aib-Glu-Gly-Thr-**Phe(2-F)**-Thr-Ser-Asp-Bip-Bip	9.92	0.97
RXL-3030	**Aib**-Aib-Glu-Gly-Thr-**α-Me-Phe(2-F)**-Thr-Ser-Asp-Bip-Bip	8.58	RXL-3001	**Ala**-Aib-Glu-Gly-Thr-Phe(2-F)-Thr-Ser-Asp-Bip-Bip	7.86	−0.26
RXL-101	His-**Aib**-Glu-Gly-Thr-**α-Me-Phe(2-F)**-Thr-Ser-Asp-Bip-Bip	10.89	RXL-3002	His-**Ala**-Glu-Gly-Thr-Phe(2-F)-Thr-Ser-Asp-Bip-Bip	9.92	0.00
RXL-3031	His-Aib-**Aib**-Gly-Thr-**α-Me-Phe(2-F)**-Thr-Ser-Asp-Bip-Bip	8.31	RXL-3003	His-Aib-**Ala**-Gly-Thr-Phe(2-F)-Thr-Ser-Asp-Bip-Bip	8.47	−1.13
RXL-3032	His-Aib-Glu-**Aib**-Thr-**α-Me-Phe(2-F)**-Thr-Ser-Asp-Bip-Bip	7.80	RXL-3037	His-Aib-Glu-**Ala**-Thr-Phe(2F)-Thr-Ser-Asp-Bip-Bip	6.46	0.37
RXL-3033	His-Aib-Glu-Gly-**Aib-α-Me-Phe(2-F)**-Thr-Ser-Asp-Bip-Bip	8.13	RXL-3004	His-Aib-Glu-Gly-**Ala**-Phe(2-F)-Thr-Ser-Asp-Bip-Bip	8.16	−1.00
RXL-3028	His-Aib-Glu-Gly-Thr-**Aib**-Thr-Ser-Asp-Bip-Bip	6.98	RXL-3038	His-Aib-Glu-Gly-Thr-**Ala**-Thr-Ser-Asp-Bip-Bip	5.55	0.46
RXL-3034	His-Aib-Glu-Gly-Thr-**α-Me-Phe(2-F)-Aib**-Ser-Asp-Bip-Bip	7.04	RXL-3005	His-Aib-Glu-Gly-Thr-Phe(2-F)-**Ala**-Ser-Asp-Bip-Bip	7.67	−1.60
RXL-3035	His-Aib-Glu-Gly-Thr-**α-Me-Phe(2-F)**-Thr-**Aib**-Asp-Bip-Bip	6.89	RXL-3006	His-Aib-Glu-Gly-Thr-Phe(2-F)-Thr-**Ala**-Asp-Bip-Bip	8.72	−2.80
RXL-3036	His-Aib-Glu-Gly-Thr-**α-Me-Phe(2-F)**-Thr-Ser-**Aib**-Bip-Bip	7.04	RXL-3007	His-Aib-Glu-Gly-Thr-Phe(2-F)-Thr-Ser-**Ala**-Bip-Bip	8.32	−2.26
RXL-3051	His-Aib-Glu-Gly-Thr-**α-Me-Phe(2-F)**-Thr-Ser-Asp-**Aib**-Bip	6.00	RXL-3008	His-Aib-Glu-Gly-Thr-Phe(2-F)-Thr-Ser-Asp-**Ala**-Bip	6.37	−1.34
RXL-3052	His-Aib-Glu-Gly-Thr-**α-Me-Phe(2-F)**-Thr-Ser-Asp-Bip-**Aib**	8.08	RXL-3009	His-Aib-Glu-Gly-Thr-Phe(2-F)-Thr-Ser-Asp-Bip-**Ala**	6.00	1.11

**Table 5:** The 5NX2 PEP/GLP-1R-based reference model and stepwise multiple linear regression-derived QSAR models.

Model ^a^	Template	Descriptors	Equation ^b^	R^2^_adj_	AIC ^c^ AIC ^c^	p(F)	p(t1) ^d^	p(t2) ^e^	P(t3) ^f^	Q^2^
Assay	N/A	pIC_50_	N/A	0.62	−10.9 −6.1	4.1 × 10^−3^	4.1 × 10^−3^	N/A	N/A	0.53
Ref.	5NX2	NOVA2 Binding	pEC_50_ = −0.009BE − 8.58	0.37	5.11 6.00	1.8 × 10^−4^	1.8 × 10^−4^	N/A	N/A	0.24
QSAR1	5NX2	Interface Contacts	pEC_50_ = 0.203InteractionCount − 0.717	0.41	3.18 4.07	7.0 × 10^−5^	7.0 × 10^−5^	N/A	N/A	0.34
QSAR2	5NX2	YASARA Packing	pEC_50_ = −0.0018BindPack1D + 2.63	0.31	8.00 8.89	7.5 × 10^−4^	7.5 × 10^−4^	N/A	N/A	0.20
QSAR3	5NX2	NOVA2 Terms	pEC_50_ = −0.026BEpot + 0.019BSvdw − 7.26	0.58	−6.95 −5.41	1.8 × 10^−6^	8.6 × 10^−4^	0.009	N/A	0.53
QSAR4	5NX2	Mixed	pEC_50_ = −0.029BEpot + 0.037BSvdw + 0.00038BindPack1D − 9.13	0.63	−9.81 −7.41	1.2 × 10^−6^	1.9 × 10^−4^	0.0017	0.04	0.54

^a^ Assay refers to the comparative experimental pIC_50_ assay results of Adelhorst et al. and Gallwitz et al., Ref. refers to the NOVA2 single-term reference computational model, and QSAR refers to the computational QSAR models. ^b^ BE refers to NOVA2 force field receptor-ligand binding energy (BE = BEpot − BS − BShydro, where BEpot is the sum of the vdw and electrostatic potential energies of binding, BS is the sum of the vdw and electrostatic solvation free energies of binding, and BShydro refers to the binding hydrophobic effect), InteractionCount refers to the total number of favorable interface contacts (*InteractionCount = H-bond count + Hydrophobic count + Cation-π count + Pi-Pi count + Ion-Ion count*), BindPack1D refers to the YASARA force field 1D binding packing score (*BindPack1D = CompPack1D − ReceptorPack1D − PeptidePack1D*), BEpot refers to the NOVA2 force field binding potential energy alone, and BSvdw refers to the vdw contribution to the binding-induced solvation free energy alone. ^c^ AIC and AICc refer to the Akaike Information Criterion and corrected Akaike Information Criterion. ^d,e,f^ p(t) refers to the probability of the t-score for the first, second, and third terms of the regression equation, respectively. See the Materials and Methods section for more details.

**Table 6:** Potency profile of key ultra-short GLP-1 analogs.

Peptide	Peptide Structure	EC_50_ (nM)	E_MAX_, %	RXL-3000	RXL-101
**RXL-3000**	His-Aib-Glu-Gly-Thr-Phe(2-F)-Thr-Ser-Asp-Bip-Bip-NH_2_	0.1	109.2	PARENT	
**RXL-3001**	**Ala**-Aib-Glu-Gly-Thr-Phe(2-F)-Thr-Ser-Asp-Bip-Bip-NH_2_	21.9	107.5		
**RXL-3002**	His-**Ala**-Glu-Gly-Thr-Phe(2-F)-Thr-Ser-Asp-Bip-Bip-NH_2_	0.2	102.5		
**RXL-3003**	His-Aib-**Ala**-Gly-Thr-Phe(2-F)-Thr-Ser-Asp-Bip-Bip-NH_2_	7.9	108.2		
**RXL-3037**	His-Aib-Glu-**Ala**-Thr-Phe(2-F)-Thr-Ser-Asp-Bip-Bip-NH_2_	210.0	73.8		
**RXL-3004**	His-Aib-Glu-Gly-**Ala**-Phe(2-F)-Thr-Ser-Asp-Bip-Bip-NH_2_	7.7	108.2		
**RXL-3038**	His-Aib-Glu-Gly-Thr-**Ala**-Thr-Ser-Asp-Bip-Bip-NH_2_	>1000	37.4		
**RXL-3005**	His-Aib-Glu-Gly-Thr-Phe(2-F)-**Ala**-Ser-Asp-Bip-Bip-NH_2_	47.5	102.4		
**RXL-3006**	His-Aib-Glu-Gly-Thr-Phe(2-F)-Thr-**Ala**-Asp-Bip-Bip-NH_2_	8.5	120.1		
**RXL-3007**	His-Aib-Glu-Gly-Thr-Phe(2-F)-Thr-Ser-**Ala**-Bip-Bip-NH_2_	14	131.6		
**RXL-3008**	His-Aib-Glu-Gly-Thr-Phe(2-F)-Thr-Ser-Asp-**Ala**-Bip-NH_2_	>1000	43.6		
**RXL-3009**	His-Aib-Glu-Gly-Thr-Phe(2-F)-Thr-Ser-Asp-Bip-**Ala**-NH_2_	>1000	43.2		
**RXL-101**	His-Aib-Glu-Gly-Thr-***α***-**Me-Phe(2-F)**-Thr-Ser-Asp-Bip-Bip-NH_2_	0.01	116.7		PARENT
**RXL-3030**	**Aib-**Aib-Glu-Gly-Thr-***α***-**Me-Phe(2-F)**-Thr-Ser-Asp-Bip-Bip-NH_2_	1.3	111.6		
**RXL-3031**	His-Aib-**Aib**-Gly-Thr-***α***-**Me-Phe(2-F)**-Thr-Ser-Asp-Bip-Bip-NH_2_	4.0	121.3		
**RXL-3032**	His-Aib-Glu-**Aib**-Thr-***α***-**Me-Phe(2-F)**-Thr-Ser-Asp-Bip-Bip-NH_2_	14.2	117.7		
**RXL-3033**	His-Aib-Glu-Gly-**Aib**-*****α***-Me-Phe(2-F)**-Thr-Ser-Asp-Bip-Bip-NH_2_	6.9	119.9		
**RXL-3028**	His-Aib-Glu-Gly-Thr-**Aib**-Thr-Ser-Asp-Bip-Bip-NH_2_	161.0	76.1		
**RXL-3034**	His-Aib-Glu-Gly-Thr-***α***-**Me-Phe(2-F)**-**Aib**-Ser-Asp-Bip-Bip-NH_2_	80.2	105.7		
**RXL-3035**	His-Aib-Glu-Gly-Thr-***α***-**Me-Phe(2-F)**-Thr-**Aib**-Asp-Bip-Bip-NH_2_	111.0	79.1		
**RXL-3036**	His-Aib-Glu-Gly-Thr-***α***-**Me-Phe(2-F)**-Thr-Ser-**Aib**-Bip-Bip-NH_2_	67.5	96.7		
**RXL-3051**	His-Aib-Glu-Gly-Thr-***α***-**Me-Phe(2-F)**-Thr-Ser-Asp-**Aib**-Bip-NH_2_	>1000	26.8		
**RXL-3052**	His-Aib-Glu-Gly-Thr-***α***-**Me-Phe(2-F)**-Thr-Ser-Asp-Bip-**Aib**-NH_2_	7.4	112.3		
**RXL-100**	His-Aib-Glu-Gly-Thr-**Phe**-Thr-Ser-Asp-Bip-Bip-NH_2_	0.5	109.6		
**RXL-3010**	His-Aib-Glu-Gly-Thr-**Phe(2-Br)**-Thr-Ser-Asp-Bip-Bip-NH_2_	3.9	120.6		
**RXL-3011**	His-Aib-Glu-Gly-Thr-**Phe(2-Cl)**-Thr-Ser-Asp-Bip-Bip-NH_2_	0.9	91.3		
**RXL-3012**	His-Aib-Glu-Gly-Thr-**Phe(2-CF_3_)**-Thr-Ser-Asp-Bip-Bip-NH_2_	13.9	99.3		
**RXL-3013**	His-Aib-Glu-Gly-Thr-**Phe(2-CH_3_)**-Thr-Ser-Asp-Bip-Bip-NH_2_	2.1	98.6		
**RXL-3014**	His-Aib-Glu-Gly-Thr-**Phe(2-NO_2_)**-Thr-Ser-Asp-Bip-Bip-NH_2_	1.1	104.5		
**RXL-3015**	His-Aib-Glu-Gly-Thr-**Phe(2-CN)**-Thr-Ser-Asp-Bip-Bip-NH_2_	7.7	101		
**RXL-3016**	His-Aib-Glu-Gly-Thr-**Phe(3-CF_3_)**-Thr-Ser-Asp-Bip-Bip-NH_2_	13.9	108.9		
**RXL-3017**	His-Aib-Glu-Gly-Thr-**Phe(3,4,5-F)**-Thr-Ser-Asp-Bip-Bip-NH_2_	7.2	113.1		
**RXL-3018**	His-Aib-Glu-Gly-Thr-**Phe(2,3,4,5,6-F)**-Thr-Ser-Asp-Bip-Bip-NH_2_	0.3	102.5		
**RXL-3019**	His-Aib-Glu-Gly-Thr-**hPhe**-Thr-Ser-Asp-Bip-Bip-NH_2_	16.0	109.5		
**RXL-3020**	His-Aib-Glu-Gly-Thr**-*α***-**Me-Phe-**Thr-Ser-Asp-Bip-Bip-NH_2_	0.02	107.9		
**RXL-3021**	His-Aib-Glu-Gly-Thr-**Tyr**-Thr-Ser-Asp-Bip-Bip-NH_2_	3.0	100.2		
**RXL-3022**	His-Aib-Glu-Gly-Thr-**D-Phe-**Thr-Ser-Asp-Bip-Bip-NH_2_	23.2	101.4		
**RXL-3023**	His-Aib-Glu-Gly-Thr-**Trp**-Thr-Ser-Asp-Bip-Bip-NH_2_	13.8	106.3		
**RXL-3024**	His-Aib-Glu-Gly-Thr-**Bip**-Thr-Ser-Asp-Bip-Bip-NH_2_	10.8	102.3		

The ultra-short GLP-1 analogs are grouped according to the Ala-scan, Aib-scan, and Phe^6^ modifications. Amino acid substitutions compared to RXL-3000 are in **bold**. The EC_50_ and E_MAX_ as well as the relative potency (see color scheme below) of the various analogs are provided as compared to the parent peptides (RXL-3000 and RXL-101). “OR” means out of the range of the assay conditions. “N/A” means not applicable.
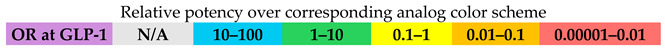

## Data Availability

Data are contained within the article.

## Supplementary Materials

The following supporting information can be downloaded at: https://www.mdpi.com/article/10.3390/molecules30010012/s1.

## Abbreviations

MLR	Multiple linear regression
SW-MLR	Stepwise multiple linear regression
R^2^	Correlation (R) squared goodness-of-fit for training set MLR/experimental data
R^2^_adj_	Parameter adjusted goodness-of-fit for the training set MLR/experimental data
p(f-test)	f-test probability of regression model
p(*t*-test)	*t*-test probability of regression model terms
VIF	Variance inflation factor obtained in regression analysis
Q^2^	Leave-one-out cross-validation R^2^ obtained using a training set
AIC	Akaike Information Criterion
AIC_c_	Corrected Akaike Information Criterion
R^2^_pred_	Correlation (R) squared goodness-of-fit for test set predictions
RMSE	Root mean squared error for training set MLR/experimental data
RMSE_pred_	Root mean squared error for test set predictions

## References

[1] Nauk M.A., Quast D.R., Wefers J., Meier J.J. (2021). GLP-1 receptor agonists in the treatment of type 2 diabetes –– state-of-the-art. Mol. Metab..

[2] Suzuki R., Brown G.A., Christopher J.A., Scully C.C.G., Congress M. (2020). Recent developments in therapeutic peptides for the glucagon-like peptide 1 and 2 receptors. J. Med. Chem..

[3] Knudsen L.B., Lau J. (2019). The discovery and development of liraglutide and semaglutide. Front. Endocrinol..

[4] Mojsov S., Weir G.C., Habener J.F. (1987). Insulinotropin: Glucagon-like peptide I (7-37) co-encoded in the glucagon gene is a potent stimulator of insulin release in the perfused rat pancreas. J. Clin. Investig..

[5] Agersø H., Jensen L.B., Elbrønd B., Rolan P., Zdravkovic M. (2002). The pharmacokinetics, pharmacodynamics, safety and tolerability of NN2211, a new long-acting GLP-1 derivative, in healthy men. Diabetologia.

[6] Naver S.V., Jimenez-Solem E., Christensen M., Andersen J.T., Knop F.K. (2014). Dulaglutide: A novel once-weekly glucagon-like peptide-1 receptor agonist. Clin. Investig..

[7] Lau J., Bloch P., Schäffer L., Pettersson I., Spetzler J., Kofoed J., Madsen K., Knudsen L.B., McGuire J., Steensgaard D.B. (2015). Discovery of the once-weekly glucagon-like peptide-1 (GLP-1) analogue semaglutide. J. Med. Chem..

[8] Sawyer J.S. (2022). Structure-Activity Studies of α-melanotropin and Ultra-Short GLP-1 Analogs to Benchmark the Development of Improved Anti-Obesity Drugs. Ph.D. Dissertation.

[9] Adelhorst K., Hedegaard, Knudsen L.B., Kirk O. (1994). Structure-activity studies of glucagon-like peptide-1. J. Biol. Chem..

[10] Gallwitz B., Witt M., Paetzold G., Morys-Wortmann C., Zimmerman B., Eckart K., Folsch U.R., Schmidt W.E. (1994). Structure/activity characterization of glucagon-like peptide-1. Eur. J. Biochem..

[11] Mapelli C., Natarajan S.I., Meyer J.P., Bastos M.M., Bernatowicz M.S., Lee V.G., Pluscec J., Riexinger D.J., Sieber-McMaster E.S., Constantine K.L. (2009). Eleven amino acid glucagon-like peptide-1 receptor agonists with antidiabetic activity. J. Med. Chem..

[12] Haque T.S., Lee V.G., Riexinger D., Lei M., Malmstrom S., Xin L., Han S., Mapelli C., Cooper C.B., Zhang G. (2010). Identification of potent 11-mer glucagon-like peptide-1 receptor peptides with novel c-terminal amino acids: Homohomophenylalanine analogs. Peptides.

[13] Haque T.S., Martinez R.L., Lee V.G., Riexinger D.G., Lei M., Feng M., Koplowitz B., Mapelli C., Cooper C.B., Zhang G. (2010). Exploration of structure-activity relationships at the two c-terminal residues of potent 11-mer glucagon-like peptide-1 receptor agonist peptides via parallel synthesis. Peptides.

[14] Yabut J.M., Drucker D.J. (2023). Glucagon-like peptide-1 receptor-based therapeutics for metabolic liver disease. Endocr. Rev..

[15] Palani A., Nawrocki A.R., Orvieto F., Bianchi E., Mandic E., Pessi A., Huang C., Deng Q., Toussaint N., Walsh E. (2022). Discovery of MK-1462: GlP-1 and glucagon receptor dual agonist for the treatment of obesity and diabetes. ACS Med. Chem. Lett..

[16] Griffith D.A., Edmonds D.J., Fortin J.P., Kalgutkar A.S., Kuzmiski J.B., Loria P.M., Saxena A.R., Bagley S.W., Buckeridge C., Curto J.M. (2022). A small molecule oral agonist of the human glucagon-like peptide-1 receptor. J. Med. Chem..

[17] Cong Z., Zhou Q., Li Y., Chen L.N., Zhang Z.C., Liang A., Liu Q., Wu X., Dai A., Xia T. (2022). Structural basis of peptidomimetic agonist revealed by small-molecule GLP-1R agonists Boc5 and WB4-24. Proc. Natl. Acad. Sci. USA.

[18] Girdhar K., Thakur S., Gaur P., Choubey A., Dogra S., Dehury B., Kumar S., Biswas B., Dwivedi D.K., Ghosh S. (2022). Design, synthesis, and biological evaluation of a small molecule oral agonist or the glucagon-like-peptide-1 receptor. J. Biol. Chem..

[19] Decara J.M., Vazquez-Villa H., Brea J., Alonso M., Srivastava R.K., Orio L., Alen F., Suarez J., Baixeras E., Garcia-Carceles J. (2022). Discovery of V-0219: A small-molecule positive allosteric modulator of the glucagon-like peptide-1 receptor toward oral treatment for “diabesity. J. Med. Chem..

[20] Zhang Y., Sun B., Feng D., Hu H., Chu M., Qu Q., Tarrasch J.T., Li S., Sun Kobilka T., Kobilka B.K. (2017). Cryo-EM structure of the activated GLP-1 receptor in complex with a G protein. Nature.

[21] Liang Y.L., Khoshouei M., Glukhova A., Furness S.G.B., Zhao P., Clydesdale L., Koole C., Truong T.T., Thal D.M., Lei S. (2018). Phase-plate cryo-EM structure of a biased agonist-bound human GLP-1 receptor-Gs complex. Nature.

[22] Oddo A., Mortensen S., Thogersen H., De Maria L., Hennen S., McGuire J.N., Kofoed J., Linderoth L., Reedtz-Runge S. (2018). α-Helix or β-turn? An investigation into N-terminally constrained analogues of glucagon-like peptide-1 (GLP-1) and exendin-4. Biochemistry.

[23] Zhang X., Belousoff M.J., Zhao P., Kooistra A.J., Truong T.T., Ang S.Y., Underwood C.R., Egebjerg T., Šenel P., Stewart G.D. (2020). Differential GLP-1R binding and activation and peptide and non-peptide agonists. Mol. Cell.

[24] Jazayeri A., Rappas M., Brown A.J.H., Kean J., Errey J.C., Robertson N.J., Fiez-Vandal C., Andrews S.P., Congreve M., Bortolato A. (2017). Crystal structure of the GLP-1 receptor bound to a peptide agonist. Nature.

[25] Zhao P., Liang Y.L., Belousoff M.J., Deganutti G., Fletcher M.M., Willard F.S., Bell M.G., Christe M.E., Sloop K.W., Inoue A. (2020). Activation of the GLP-1 receptor by a non-peptide agonist. Nature.

[26] YASARA. http://www.yasara.org.

[27] Chen N., Chen J., Yao B., Li Z. (2018). QSAR study on antioxidant tripeptides and the antioxidant activity of the designed tripeptides in free radical systems. Molecules.

[28] Akbari S., Zebardast T., Zarghi A., Hajimahdi Z. (2017). QSAR modeling of COX-2 inhibitory activity of some dihydropyridine and hydroquinoline derivatives using multiple linear regression (MLR) method. Iran. J. Pharm. Res..

[29] Ghafourian T., Amin Z. (2013). QSAR models for the prediction of plasma protein binding. Bioimpacts.

[30] Tomorowicz L., Slawinski J., Zolnowska B., Szafranski K., Kawiak A. (2020). Synthesis, antitumor evaluation, molecular modeling and quantitative structure-activity relationship (QSAR) of novel 2-[(4-Amino-6-*N*-substituted-1,3,5-triazin-2-yl)methylthio]-4-chloro-5-methyl-*N*-(1 *H*-benzo[*d*]imidazol-2(3 *H*)-ylidene)benzenesulfonamides. Int. J. Mol. Sci..

[31] Chen M., Yang F., Kang J., Gan H., Yang X., Lai X., Gao Y. (2018). Identification of potent LXRβ-selective agonists without LXRα activation by in silico approaches. Molecules.

[32] Hajimahdi Z., Safizadeh F., Zarghi Z. (2016). QSAR analysis for some 1,2-Benzisothiazol-3-one derivatives as caspase-3 inhibitors by stepwise MLR method. Iran. J. Pharm. Res..

[33] Scior T., Medina-Franco J.L., Do Q.-T., Martinez-Mayorga K., Yunes Rojas J.A., Bernard P. (2009). How to recognize and workaround pitfalls in QSAR studies: A critical review. Curr. Med. Chem..

[34] Daoui O., Elkhattabi S., Chtita S., Elkhalabi R., Zgou H., Benjelloun A.T. (2021). QSAR, molecular docking and ADMET properties in silico studies of novel 4,5,6,7-tetrahydrobenzo[D]-thiazol-2-Yl derivatives derived from dimedone as potent anti-tumor agents through inhibition of C-Met receptor tyrosine kinase. Heliyon.

[35] Alexander D.L., Tropsha A., Winkler D.A. (2015). Beware of R(2): Simple, unambiguous assessment of the prediction accuracy of QSAR and QSPR models. J. Chem. Inf. Model..

[36] Diller D.J., Swanson J., Bayden A.S., Brown C.J., Thean D., Lane D.P., Partridge A.W., Sawyer T.K., Audie J. (2019). Rigorous computational and experimental investigations on MDM2/MDMX-targeted linear and macrocyclic peptides. Molecules.

[37] Wang J., Wang W., Kollman P.A., Case D.A. (2006). Automatic atom type and bond type perception in molecular mechanical calculations. J. Mol. Graph. Model..

[38] Wang J., Wolf R.M., Caldwell J.W., Kollman P.A., Case D.A. (2004). Development and testing of a general amber force field. J. Comput. Chem..

[39] Abagyan R., Totrov M. (1994). Biased probability Monte Carlo conformational searches and electrostatic calculations for peptides and proteins. J. Mol. Biol..

[40] Lazaridis T., Karplus M. (1999). Effective energy function for proteins in solution. Proteins.

[41] Ozvoldik K., Stockner T., Rammner B., Krieger E. (2021). Assembly of biomolecular gigastructures and visualization with the Vulkan graphics API. J. Chem. Inf. Model..

[42] Krieger E., Vriend G. (2014). YASARA View—Molecular graphics for all devices—From smartphones to workstations. Bioinformatics.

[43] Krieger E., Joo K., Lee J., Lee J., Raman S., Thompson J., Tyka M., Baker D., Karplus K. (2009). Improving physical realism, stereochemistry, and side-chain accuracy in homology modeling: Four approaches that performed well in CASP8. Proteins.

[44] Krieger E., Koraimann G., Vriend G. (2002). Increasing the precision of comparative models with YASARA NOVA--a self-parameterizing force field. Proteins.

[45] Klamt A. (1995). Conductor-like screening model for real solvents: A new approach to the quantitative calculation of solvation phenomena. J. Phys. Chem..

[46] Jakalian A., Jack D.B., Bayly C.I. (2002). Fast, efficient generation of high-quality atomic charges. AM1-BCC model: II. parameterization and validation. J. Comput. Chem..

