# Supplementary material for: Design, Structure–Activity Relationships, and Computational Modeling Studies of a Series of α-Helix Biased, Ultra-Short Glucagon-like Peptide-1 Receptor Agonists
Source: Molecules. 2024 Dec 24;30(1):12. doi: 10.3390/molecules30010012 (PMC11721672; doi:10.3390/molecules30010012)

Supplementary Table S1. Analytical data and yield for the peptides encompassed in Phe^6 scan.

GLP-1 ID	MW (g/mol)	LC/MS Method and Gradient	Yield (mg)	Purity (%)	R.T. (min)	%B	Most Abundant Ion	Ion Charge
**RXL-100**	1423.55	5-65%B over 20 min @ 40°C	19.0	90.0	15.1	44.3	712.5	2
**RXL-3000**	1441.54	5-65%B over 20 min @ 40°C	12.8	90.0	15.2	44.6	721.4	2
**RXL-3010**	1502.45	5-65%B over 20 min @ 40°C	18.9	96.0	15.5	45.5	752.3	2
**RXL-3011**	1457.99	5-65%B over 20 min @ 40°C	10.0	88.0	15.5	45.5	729.4	2
**RXL-3012**	1491.55	5-65%B over 20 min @ 40°C	14.9	98.0	15.9	46.7	746.4	2
**RXL-3013**	1437.58	5-65%B over 20 min @ 40°C	21.0	99.0	15.5	45.5	719.5	2
**RXL-3014**	1468.55	5-65%B over 20 min @ 40°C	14.8	91.0	15.1	44.3	734.9	2
**RXL-3015**	1448.56	5-65%B over 20 min @ 40°C	17.5	80.0	14.9	43.7	725	2
**RXL-3016**	1491.55	5-65%B over 20 min @ 40°C	18.4	89.0	16.1	47.3	746.4	2
**RXL-3017**	1477.52	5-65%B over 20 min @ 40°C	16.6	93.0	15.6	45.8	739.4	2
**RXL-3018**	1513.50	5-65%B over 20 min @ 40°C	11.2	92.0	15.7	46.1	757.4	2
**RXL-3019**	1437.58	5-65%B over 20 min @ 40°C	11.0	99.0	15.6	45.8	719.4	2
**RXL-3020**	1437.58	5-65%B over 20 min @ 40°C	23.4	78.0	16.0	47.13	719.4	2
**RXL-3021**	1439.55	5-65%B over 20 min @ 40°C	19.5	93.0	14.4	42.2	720.4	2
**RXL-3022**	1423.55	5-65%B over 20 min @ 40°C	11.1	89.0	15.6	45.8	712.5	2
**RXL-3023**	1462.59	5-65%B over 20 min @ 40°C	13.0	96.0	15.4	45.2	731.9	2
**RXL-3024**	1499.65	5-65%B over 20 min @ 40°C	9.4	88.0	16.7	49.1	750.5	2

All purified peptides were analyzed by LC/MS. A Zorbax Agilent 300SB-C18 column was used. The flow rate was 0.8 mL/min. R.T. refers to retention time. %B indicates when the peptide eluted on the gradient. The peptide purity was determined by HPLC at a wavelength of 214 nm. The most abundant target ion is indicated as well as the charge.

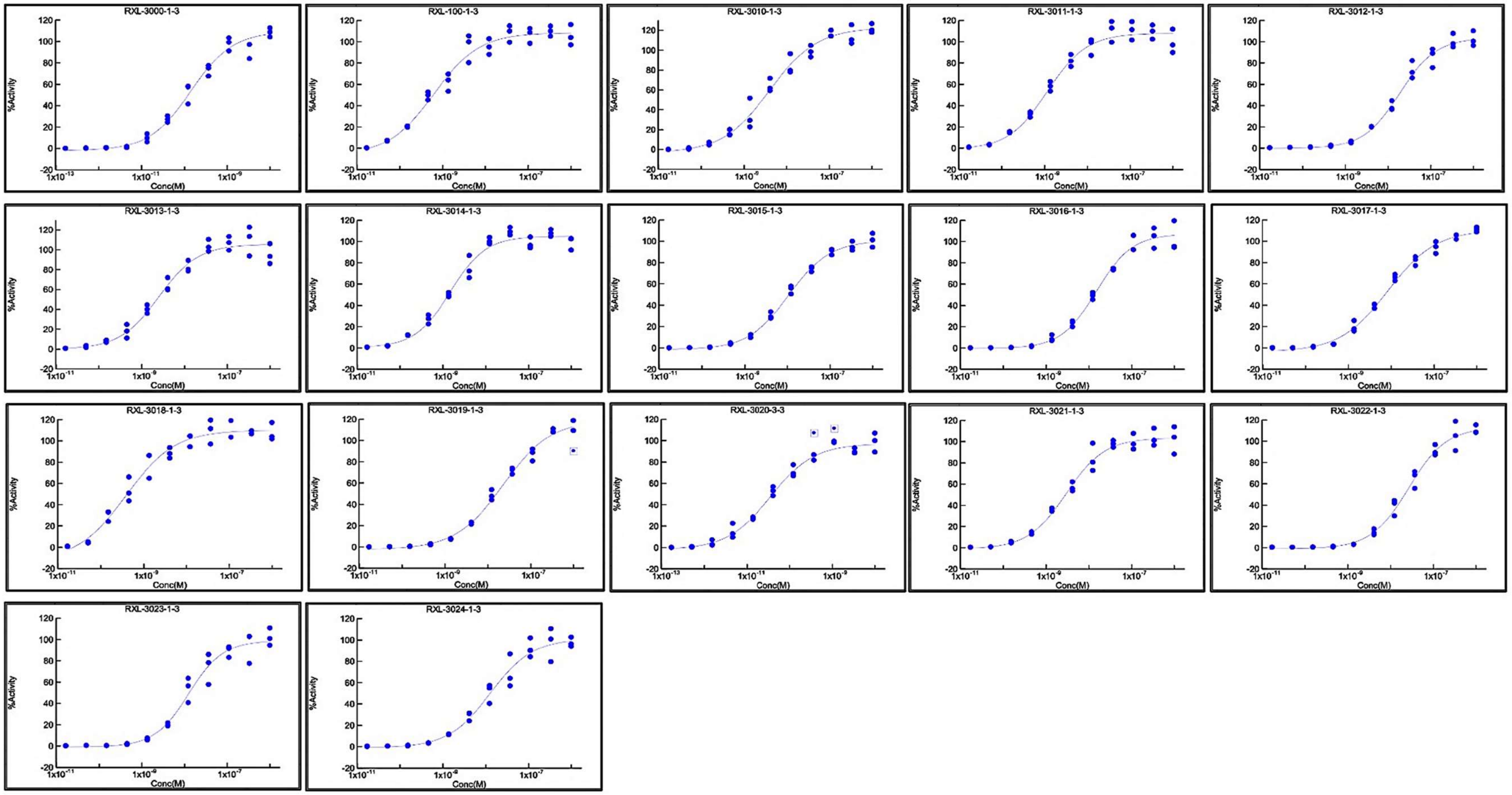

Supplementary Figure S1. Experiment 1 cAMP assay raw data curves for all ultra-short GLP-1 position 6 analogs. Each concentration was tested in triplicate. Non-linear regression was applied to construct a sigmoid variable slope using GraphPad Prism 5.

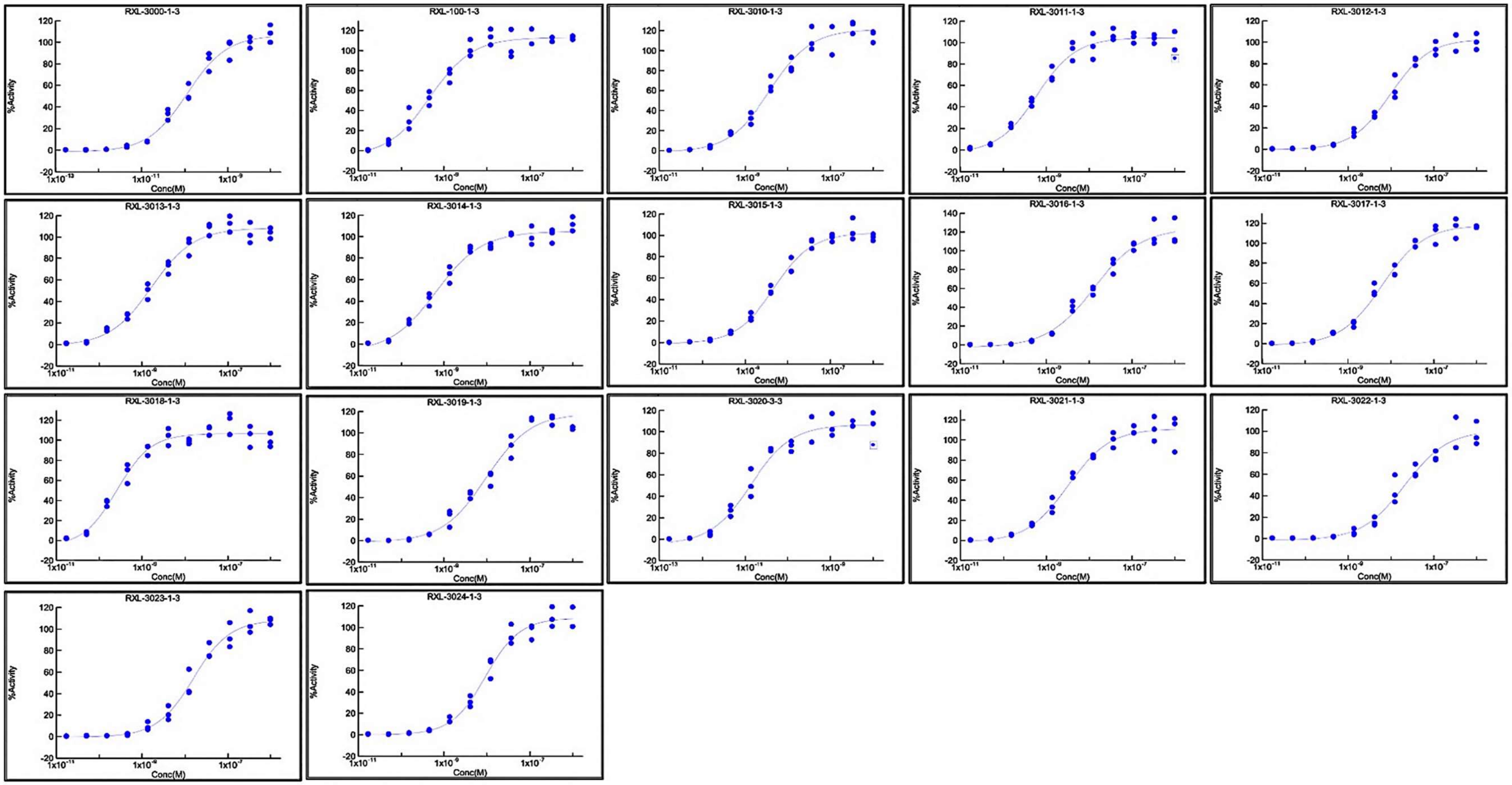

Supplementary Figure S2. Experiment 2 cAMP assay raw data curves for all ultra-short GLP-1 position 6 analogs. Each concentration was tested in triplicate. Non-linear regression was applied to construct a sigmoid variable slope using GraphPad Prism 5.

Supplementary Table S2. Analytical data and yield for the peptides encompassed in the Ala-scan.

GLP-1 ID	MW (g/mol)	LC/MS Method and Gradient	Yield (mg)	Purity (%)	R.T. (min)	%B	Most Abundant Ion	Ion Charge
**RXL-3000**	1441.54	5-65%B over 20 min @ 40°C	12.8	90	15.2	44.6	721.4	2
**RXL-3001**	1375.48	5-65%B over 20 min @ 40°C	11.3	93	15.6	45.8	688.4	2
**RXL-3002**	1427.51	5-65%B over 20 min @ 40°C	4.5	90	15.1	44.3	714.4	2
**RXL-3003**	1383.50	5-65%B over 20 min @ 40°C	14.3	84	15.3	44.9	692.4	2
**RXL-3004**	1411.51	5-65%B over 20 min @ 40°C	7.7	94	15.2	44.6	706.4	2
**RXL-3005**	1411.51	5-65%B over 20 min @ 40°C	14.8	89	15.5	45.5	706.4	2
**RXL-3006**	1425.54	5-65%B over 20 min @ 40°C	13.1	87	15.4	45.2	713.4	2
**RXL-3007**	1397.53	5-65%B over 20 min @ 40°C	5.3	83	15.4	45.2	699.4	2
**RXL-3008**	1289.34	5-65%B over 20 min @ 40°C	19.1	96	11.2	32.6	645.3	2
**RXL-3009**	1289.34	5-65%B over 20 min @ 40°C	14.1	92	11.2	32.6	645.4	2
**RXL-3037**	1455.57	5-65%B over 20 min @ 40°C	21.1	93	15.3	44.9	728.4	2
**RXL-3038**	1347.45	5-65%B over 20 min @ 40°C	27.4	93	14.2	41.5	674.4	2

All purified peptides were analyzed by LC/MS. A Zorbax Agilent 300SB-C18 column was used. The flow rate was 0.8 mL/min. R.T. refers to retention time. %B indicates when the peptide eluted on the gradient. The peptide purity was determined by HPLC at a wavelength of 214 nm. The most abundant target ion is indicated as well as the charge.

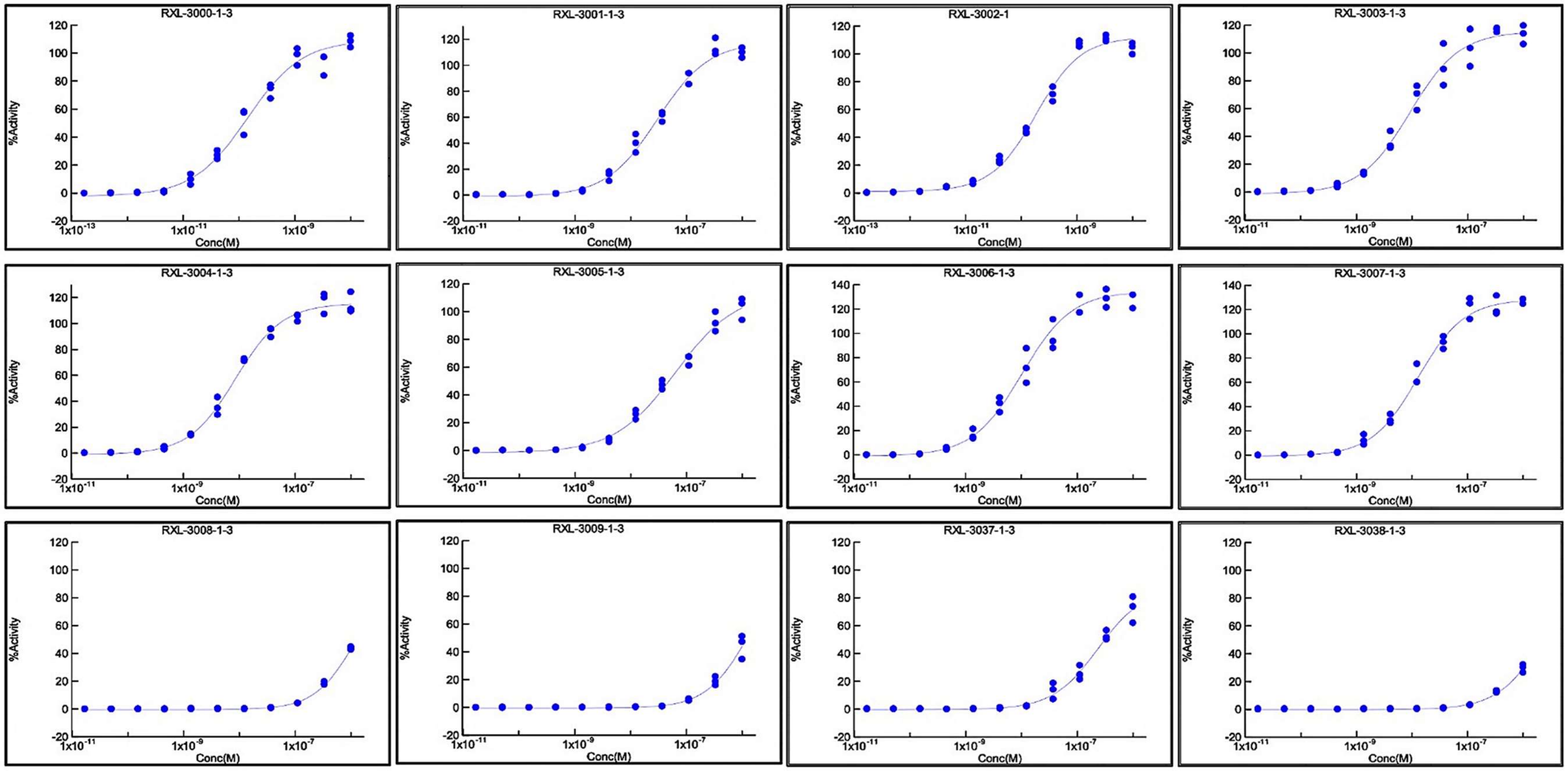

Supplementary Figure S3. Experiment 1 cAMP assay raw data curves for all ultra-short GLP-1 Ala-scan analogs. Each concentration was tested in triplicate. Non-linear regression was applied to construct a sigmoid variable slope using GraphPad Prism 5.

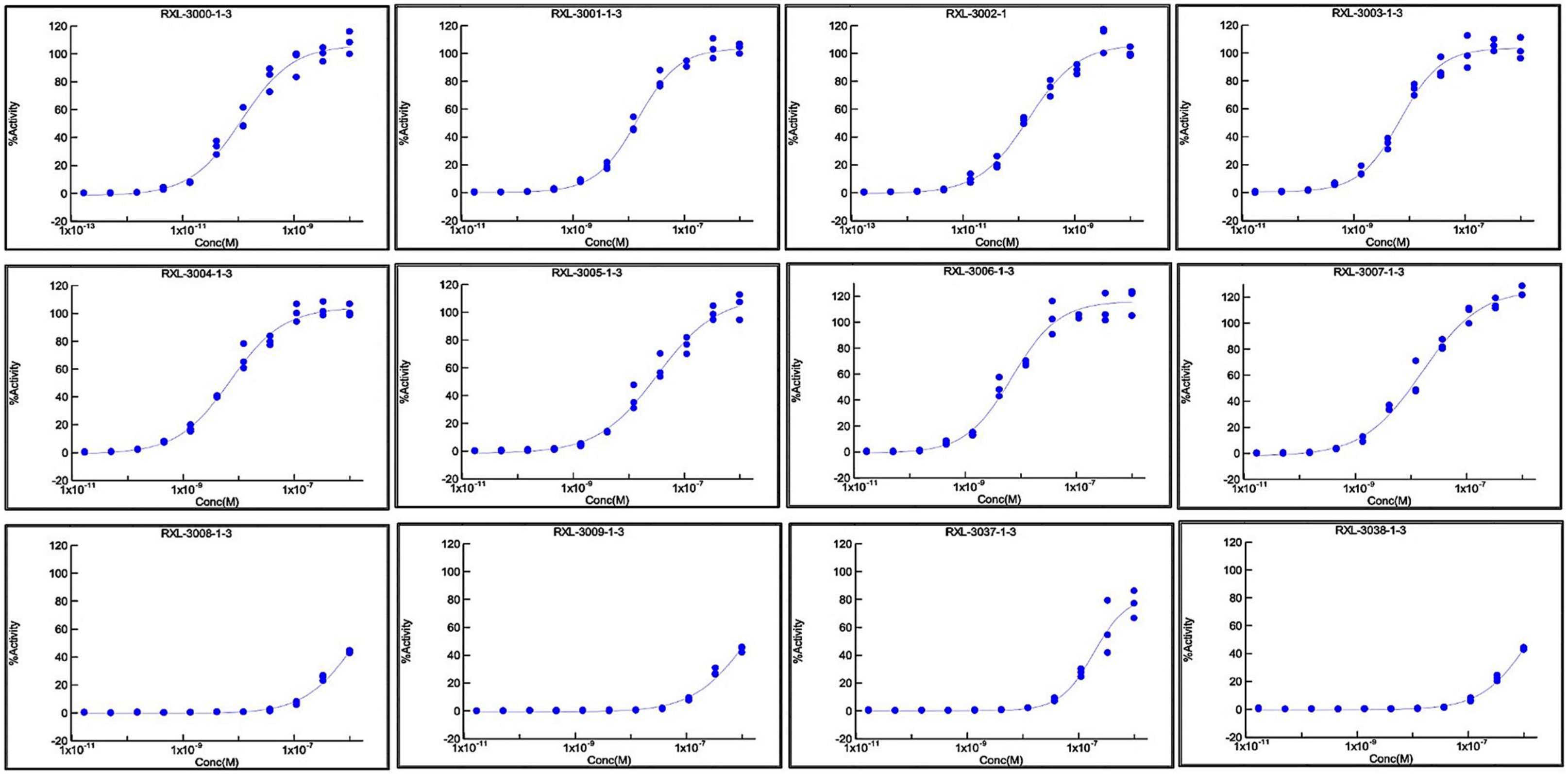

Supplementary Figure S4. Experiment 2 cAMP assay raw data curves for all ultra-short GLP-1 Ala-scan analogs. Each concentration was tested in triplicate. Non-linear regression was applied to construct a sigmoid variable slope using GraphPad Prism 5.

Supplementary Table S3. Analytical data and yield for the peptides encompassed in the Aib-scan.

GLP-1 ID	MW (g/mol)	LC/MS Method and Gradient	Yield (mg)	Purity (%)	R.T. (min)	%B	Most Abundant Ion	Ion Charge
**RXL-101**	1455.57	20-80%B over 20 min @ 40°C	9.8	92.5	11.3	47.9	728.4	2
**RXL-3028**	1361.48	5-65%B over 20 min @ 40°C	18.2	95.0	14.6	42.8	681.4	2
**RXL-3030**	1403.53	5-65%B over 20 min @ 40°C	17.5	96.0	16.5	48.5	702.4	2
**RXL-3031**	1411.56	5-65%B over 20 min @ 40°C	15.8	93.0	16.5	48.4	706.5	2
**RXL-3032**	1483.62	5-65%B over 20 min @ 40°C	16.9	92.0	16.1	47.4	742.2	2
**RXL-3033**	1439.57	5-65%B over 20 min @ 40°C	15.8	85.0	15.9	46.8	720.4	2
**RXL-3034**	1439.57	5-65%B over 20 min @ 40°C	10.7	75.0	16.9	49.7	720.4	2
**RXL-3035**	1453.59	5-65%B over 20 min @ 40°C	38.0	97.0	16.3	47.8	727.4	2
**RXL-3036**	1425.58	5-65%B over 20 min @ 40°C	12.8	98.0	16.7	49.2	713.5	2
**RXL-3051**	1317.40	20-80%B over 20 min @ 40°C	22.7	92.6	7.89	37.7	659.4	2
**RXL-3052**	1317.40	20-80%B over 20 min @ 40°C	17.3	83.0	7.98	38.0	659.4	2

All purified peptides were analyzed by LC/MS. A Zorbax Agilent 300SB-C18 column was used. The flow rate was 0.8 mL/min. R.T. refers to retention time. %B indicates when the peptide eluted on the gradient. The peptide purity was determined by HPLC at a wavelength of 214 nm. The most abundant target ion is indicated as well as the charge.

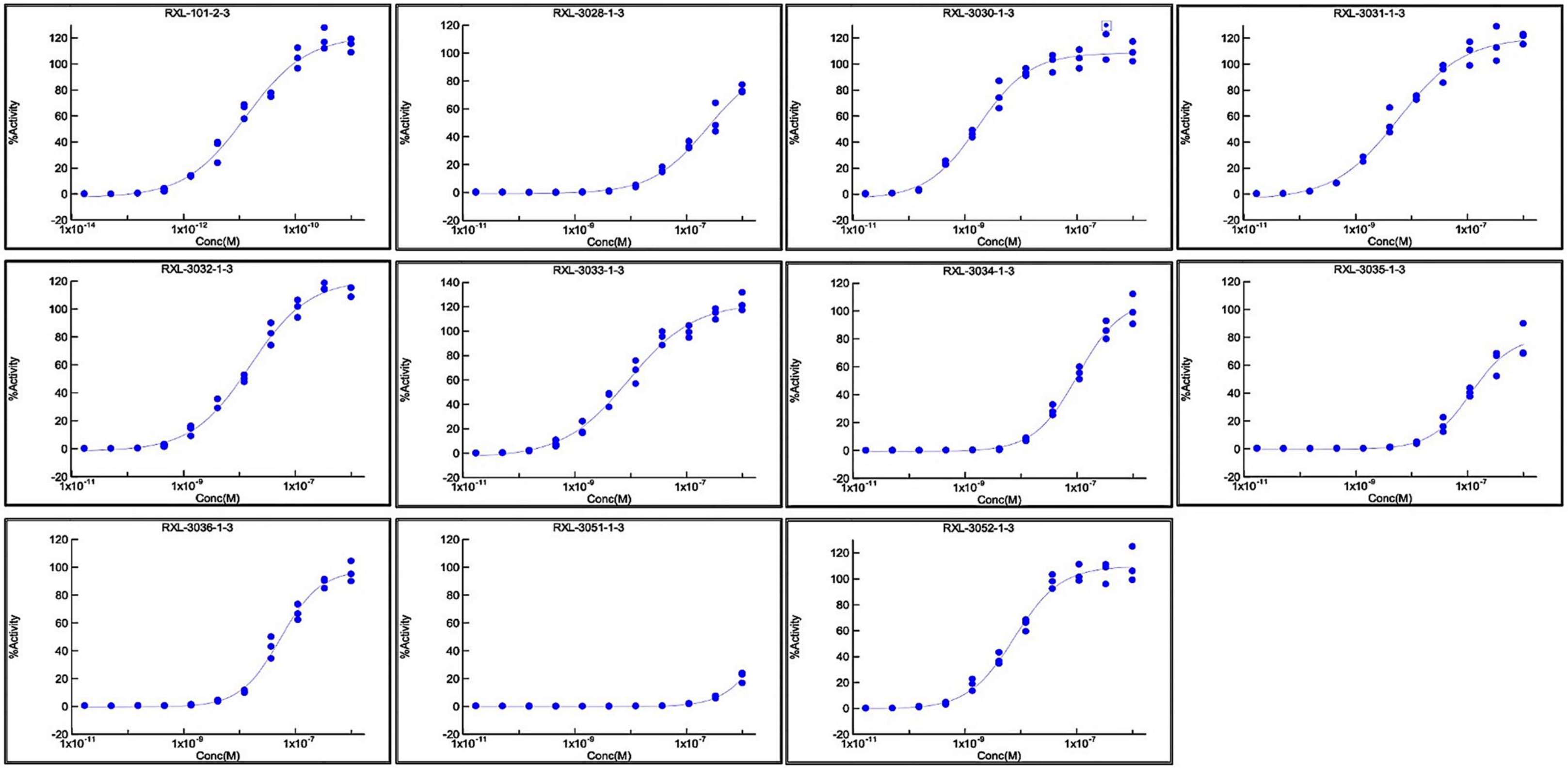

Supplementary Figure S5. Experiment 1 cAMP assay raw data curves for all ultra-short GLP-1 Aib-scan analogs. Each concentration was tested in triplicate. Non-linear regression was applied to construct a sigmoid variable slope using GraphPad Prism 5.

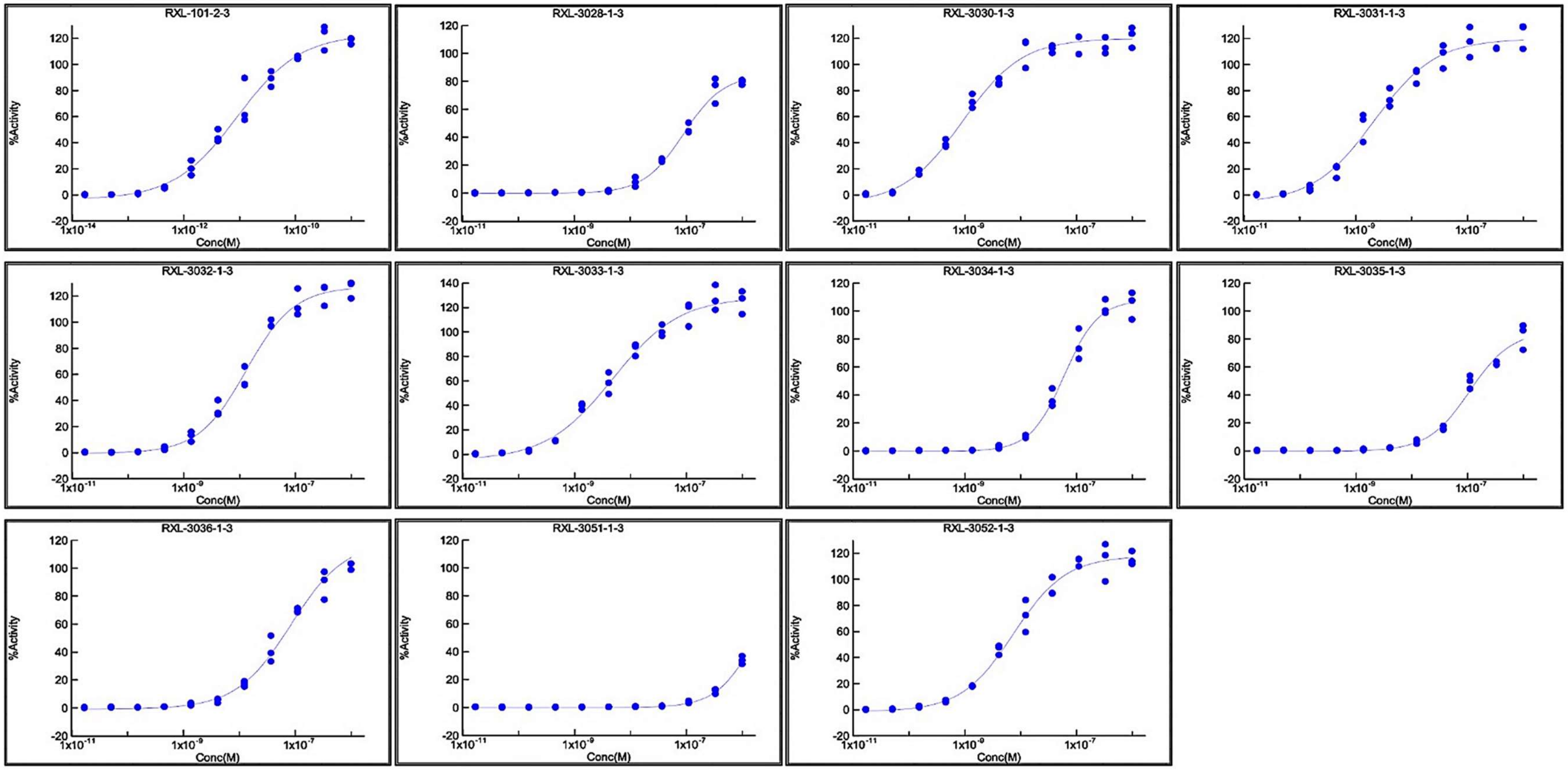

Supplementary Figure S6. Experiment 1 cAMP assay raw data curves for all ultra-short GLP-1 Aib-scan analogs. Each concentration was tested in triplicate. Non-linear regression was applied to construct a sigmoid variable slope using GraphPad Prism 5.

Supplementary Table S4. Adelhorst et al. and Gallwitz et al. residue scanning GLP-1 IC_{50} and EC_{50} data (note the non-Ala S8, R24, and Q30 results).

Alderhorts	Mutant	IC50	pIC50	EC50	pEC50	Gallwitz	Mutant	IC50	pIC50	Kd	dGexp
	GLP-1	0.27	9.57	2.6	8.59		H7A	131	6.88	176.00	-9.33
	A7	30	7.52	10000	5.00		E9A	11	7.96	9.00	-11.12
	S8	2.4	8.62	2	8.70		G10A	94	7.03	62.00	-9.96
	A9	8.1	8.09	2	8.70		T11A	3	8.52	4.00	-11.60
	A10	59	7.23	10000	5.00		F12A	47	7.33	68.00	-9.90
	A11	3.5	8.46	5	8.30		T13A	193	6.71	200.00	-9.25
	A12	36	7.44	33	7.48		S14A	22	7.66	21.00	-10.61
	A13	36	7.44	65	7.19		D15A	69	7.16	65.00	-9.93
	A14	0.76	9.12	5	8.30		V16A	3	8.52	3.00	-11.77
	A15	11	7.96	10000	5.00		S17A	2	8.70	2.00	-12.02
	A16	1.7	8.77	7	8.15		S18A	1	9.00	1.00	-12.43
	A17	0.46	9.34	3	8.52		Y19A	83	7.08	119.00	-9.57
	A18	0.68	9.17	2	8.70		L20A	11	7.96	10.00	-11.05
	A19	3.5	8.46	55	7.26		E21A	46	7.34	57.00	-10.01
	A20	1.7	8.77	7	8.15		G22A	1	9.00	1.00	-12.43
	A21	4.1	8.39	65	7.19		Q23A	1	9.00	1.00	-12.43
	A22	0.57	9.24	4	8.40		K26A	4	8.40	3.00	-11.77
	A23	1.1	8.96	5	8.30		E27A	9	8.05	77.00	-9.83
	R24	0.89	9.05	17	7.77		F28A	1000	6.00	1000.00	-8.29
	A26	1.4	8.85	13	7.89		I29A	137	6.86	203.00	-9.25
	A27	0.24	9.62	1	9.00		W31A	11	7.96	10.00	-11.05
	A28	351	6.45	2600	5.59		L32A	41	7.39	40.00	-10.22
	A29	25	7.60	70	7.15		V33A	6	8.22	4.00	-11.60
	Q30	1.4	8.85	0.5	9.30		K34A	2	8.70	2.00	-12.02
	A31	1.6	8.80	15	7.82		G35A	5	8.30	4.00	-11.60
	A32	4.7	8.33	4	8.40		R36A	5	8.30	3.00	-11.77
	A33	1.4	8.85	2	8.70						
	A34	1.7	8.77	2	8.70						
	A35	1.3	8.89	1	9.00						
	A36	4.6	8.34	7	8.15						

Supplementary Table S5. Adelhorst Ala-scanning and RXL-3000 Ala-scanning EC_{50} data.

Adelhorst					Resolute			
Mutant	IC50	pIC50	EC50	pEC50	Peptide	Peptide Sequence (N-Term, Sequence, C-Term)	cAMP E(	pEC50
GLP-1	0.27	9.57	2.6	8.59	RXL-3000	H His-Aib-Glu-Gly-Thr-Phe(2-F)-Thr-Ser-Asp-Bip-Bip NH2	0.12	9.92
A7	30	7.52	10000	5.00	RXL-3001	H Ala-Aib-Glu-Gly-Thr-Phe(2-F)-Thr-Ser-Asp-Bip-Bip NH2	13.7	7.86
A9	8.1	8.09	2	8.70	RXL-3003	H His-Aib-Ala-Gly-Thr-Phe(2-F)-Thr-Ser-Asp-Bip-Bip NH2	3.4	8.47
A10	59	7.23	10000	5.00	RXL-3037	H His-Aib-Glu-Ala-Thr-Phe(2F)-Thr-Ser-Asp-Bip-Bip NH2	347	6.46
A11	3.5	8.46	5	8.30	RXL-3004	H His-Aib-Glu-Gly-Ala-Phe(2-F)-Thr-Ser-Asp-Bip-Bip NH2	6.88	8.16
A12	36	7.44	33	7.48	RXL-3038	H His-Aib-Glu-Gly-Thr-Ala-Thr-Ser-Asp-Bip-Bip NH2	2800	5.55
A13	36	7.44	65	7.19	RXL-3005	H His-Aib-Glu-Gly-Thr-Phe(2-F)-Ala-Ser-Asp-Bip-Bip NH2	21.2	7.67
A14	0.76	9.12	5	8.30	RXL-3006	H His-Aib-Glu-Gly-Thr-Phe(2-F)-Thr-Ala-Asp-Bip-Bip NH2	1.91	8.72
A15	11	7.96	10000	5.00	RXL-3007	H His-Aib-Glu-Gly-Thr-Phe(2-F)-Thr-Ser-Ala-Bip-Bip NH2	4.74	8.32
A16	1.7	8.77	7	8.15	RXL-3008	H His-Aib-Glu-Gly-Thr-Phe(2-F)-Thr-Ser-Asp-Ala-Bip NH2	429	6.37
A17	0.46	9.34	3	8.52	RXL-3009	H His-Aib-Glu-Gly-Thr-Phe(2-F)-Thr-Ser-Asp-Bip-Ala NH2	10000	5.00

Supplementary Figure S7. Adelhorst Ala-scanning pEC50 versus RXL-3000 Ala-scanning pEC50 data.

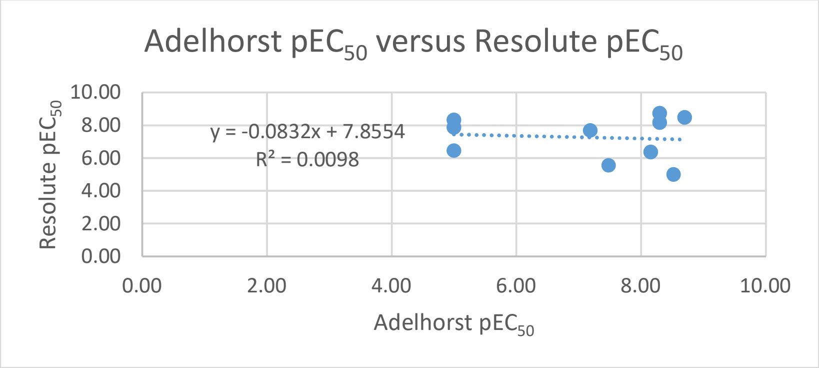

Supplementary Figure S8. Adelhorst Ala-scanning pIC50 versus Gallwitz Ala-scanning pIC50 data (Ala residue substitutions that correspond to RXL-3000 to RXL-3009 data).

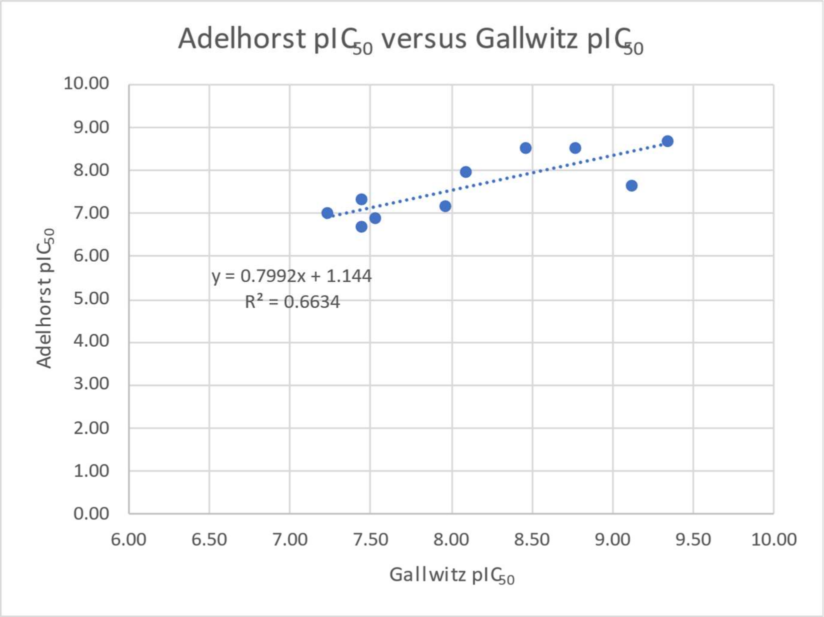

Supplementary QSAR methods and results

Supplementary Table S6. All 5NX2-based QSAR descriptors.

Peptide	Peptide Structure (N-Terminus, Amino Acid Sequence, C-Terminus)	cAMP EC50	pEC50	Molecules	bindpot	bindpotcoul	bindpotvdw	bindsolv	bindsolvcoul	bindsolvvdw	bindentropy	bindenergy	CompMQ	BindMQ	HB Energy	HB Total	H Num	H Energy	CationPI Num	CationPI Energy	IonIon	IonIon Energy	PIPI Num	PIPI Energy	Total Energy	Interface Count	CompPack1D	bindPack1D	CompPack3D	BindPack3D
Res 1 100	His-Aib-Glu-Gly-Thr-Phe-Thr-Ser-Asp-Bip-Bip	2.3	8.64	Snx2_ha_0_min	-263.21	-30.29	-232.92	724.61	256.07	468.53	-2334.79	-1872.89	-2.00	0.03	136.63	7.00	35.00	20.78	0.00	0.00	0.00	0.00	4.00	3.42	161.41	46.00	-20066.82	-3206.45	406495.77	18289.75
RXL-3000	His-Aib-Glu-Gly-Thr-Phe(2-F)-Thr-Ser-Asp-Bip-Bip	0.12	9.92	Snx2_ha_1_min	-275.38	-32.37	-243.01	734.54	265.05	469.49	-2338.32	-1879.17	-2.02	0.05	136.63	7.00	33.00	18.79	0.00	0.00	0.00	0.00	4.00	3.68	159.42	44.00	-19945.25	-3102.33	406269.84	18096.63
RXL-3011	His-Aib-Glu-Gly-Thr-Phe(2-Cl)-Thr-Ser-Asp-Bip-Bip	0.59	9.23	Snx2_ha_2_min	-277.51	-33.00	-244.51	735.52	262.02	473.50	-2338.50	-1880.49	-2.01	0.05	136.63	7.00	34.00	20.53	0.00	0.00	0.00	0.00	5.00	4.22	162.16	46.00	-20042.97	-3183.44	406316.43	18129.90
RXL-3012	His-Aib-Glu-Gly-Thr-Phe(2-CF3)-Thr-Ser-Asp-Bip-Bip	11.7	7.93	Snx2_ha_3_min	-263.04	-35.54	-227.50	744.46	270.45	474.01	-2336.63	-1855.21	-2.00	0.18	136.63	7.00	34.00	20.39	0.00	0.00	0.00	0.00	6.00	4.62	163.02	47.00	-20178.65	-3284.76	406604.87	18411.74
RXL-3013	His-Aib-Glu-Gly-Thr-Phe(2-CH3)-Thr-Ser-Asp-Bip-Bip	1.54	8.81	Snx2_ha_4_min	-275.36	-31.52	-243.84	735.47	262.57	472.90	-2334.67	-1874.56	-2.00	0.04	136.63	7.00	38.00	23.00	0.00	0.00	0.00	0.00	5.00	4.19	164.63	50.00	-20081.46	-3206.56	406788.77	18551.63
RXL-3014	His-Aib-Glu-Gly-Thr-Phe(2-NO2)-Thr-Ser-Asp-Bip-Bip	1.13	8.95	Snx2_ha_5_mi	-275.54	-32.61	-242.93	738.01	266.27	471.74	-2338.46	-1875.99	-2.01	0.26	136.63	7.00	33.00	19.95	0.00	0.00	0.00	0.00	3.00	1.96	159.58	43.00	-20327.74	-3443.99	406334.54	18129.35
RXL-3015	His-Aib-Glu-Gly-Thr-Phe(2-CN)-Thr-Ser-Asp-Bip-Bip	9.05	8.04	Snx2_ha_6_min	-256.17	-34.44	-221.73	792.49	326.79	465.70	-2338.94	-1802.62	-2.01	0.13	136.63	7.00	33.00	18.17	0.00	0.00	0.00	0.00	1.00	1.00	155.80	41.00	-20098.51	-3214.15	406827.71	18544.15
RXL-3039	His-Aib-Glu-Gly-Thr-Phe(2,6-F)-Thr-Ser-Asp-Bip-Bip	0.16	9.80	Snx2_ha_7_min	-277.86	-33.61	-244.26	733.86	265.08	468.77	-2337.19	-1881.20	-2.03	0.34	136.63	7.00	33.00	18.89	0.00	0.00	0.00	0.00	4.00	3.70	159.52	44.00	-19757.81	-2944.29	406212.51	18028.36
RXL-3017	His-Aib-Glu-Gly-Thr-Phe(3,4,5-F)-Thr-Ser-Asp-Bip-Bip	10.8	7.97	Snx2_ha_8_min	-258.79	-26.60	-232.19	743.90	273.17	470.73	-2340.33	-1855.22	-2.05	0.64	136.63	7.00	31.00	17.86	0.00	0.00	0.00	0.00	1.00	1.00	155.49	39.00	-19689.89	-2916.81	405946.66	17737.11
RXL-3001	H Ala-Aib-Glu-Gly-Thr-Phe(2-F)-Thr-Ser-Asp-Bip-Bip NH2	13.7	7.86	Snx2_ala_1_min	-260.53	-21.54	-238.99	666.38	238.64	427.75	-2290.25	-1884.39	-2.03	-0.31	120.00	6.00	33.00	18.78	0.00	0.00	0.00	0.00	4.00	3.68	142.46	43.00	-17411.16	-2459.28	440394.83	19732.64
RXL-3002	H His-Ala-Glu-Gly-Thr-Phe(2-F)-Thr-Ser-Asp-Bip-Bip NH2	0.12	9.92	Snx2_ala_2_min	-267.06	-33.66	-233.40	725.05	266.39	458.66	-2326.80	-1868.81	-2.01	-0.18	136.63	7.00	33.00	19.16	0.00	0.00	0.00	0.00	4.00	3.68	159.47	44.00	-19948.94	-3113.45	406265.74	18184.83
RXL-3003	H His-Aib-Ala-Gly-Thr-Phe(2-F)-Thr-Ser-Asp-Bip-Bip NH2	3.4	8.47	Snx2_ala_3_min	-254.20	-18.55	-235.65	682.73	247.85	439.88	-2284.97	-1856.43	-2.04	-0.15	115.90	6.00	33.00	18.79	0.00	0.00	0.00	0.00	4.00	3.68	138.37	43.00	-19636.79	-2999.63	409002.73	17908.66
RXL-3037	H His-Aib-Glu-Ala-Thr-Phe(2F)-Thr-Ser-Asp-Bip-Bip NH2	347	6.46	Snx2_ala_4_min	-188.52	-32.86	-155.66	735.20	264.03	471.17	-2335.43	-1788.75	-2.02	0.22	136.63	7.00	32.00	18.09	0.00	0.00	0.00	0.00	4.00	3.68	158.40	43.00	-19909.34	-3084.19	406498.21	18308.77
RXL-3004	H His-Aib-Glu-Gly-Ala-Phe(2-F)-Thr-Ser-Asp-Bip-Bip NH2	6.88	8.16	Snx2_ala_5_min	-277.26	-33.43	-243.83	726.17	265.01	461.16	-2320.25	-1871.34	-2.02	0.05	136.63	7.00	33.00	18.82	0.00	0.00	0.00	0.00	4.00	3.68	159.13	44.00	-19807.04	-3007.60	406203.63	18032.87
RXL-3038	H His-Aib-Glu-Gly-Thr-Ala-Thr-Ser-Asp-Bip-Bip NH2 2,80	2800	5.55	Snx2_ala_6_min	-244.31	-29.65	-214.66	643.25	247.15	401.09	-2246.41	-1847.47	-2.05	0.09	136.63	7.00	30.00	17.38	0.00	0.00	0.00	0.00	1.00	1.00	155.01	38.00	-19318.44	-2586.93	405109.67	16947.58
RXL-3005	H His-Aib-Glu-Gly-Thr-Phe(2-F)-Ala-Ser-Asp-Bip-Bip NH2	21.2	7.67	Snx2_ala_7_min	-270.36	-30.32	-240.04	719.90	264.40	455.50	-2318.22	-1868.68	-2.02	-0.04	136.63	7.00	30.00	16.99	0.00	0.00	0.00	0.00	4.00	3.68	157.30	41.00	-19812.34	-3007.76	406063.96	17914.59
RXL-3006	H His-Aib-Glu-Gly-Thr-Phe(2-F)-Thr-Ala-Asp-Bip-Bip NH2	1.91	8.72	Snx2_ala_8_min	-275.06	-33.22	-241.84	730.69	264.03	466.65	-2332.85	-1877.22	-2.02	0.06	136.63	7.00	33.00	18.79	0.00	0.00	0.00	0.00	4.00	3.68	159.10	44.00	-19895.37	-3077.13	406174.31	18020.26
RXL-3007	H His-Aib-Glu-Gly-Thr-Phe(2-F)-Thr-Ser-Ala-Bip-Bip NH2	4.74	8.32	Snx2_ala_9_min	-258.37	-15.77	-242.60	700.17	233.78	466.39	-2320.69	-1878.88	-2.03	0.16	92.08	5.00	34.00	18.90	0.00	0.00	0.00	0.00	4.00	3.68	114.66	43.00	-19849.20	-3036.43	406338.05	18195.49
RXL-3008	H His-Aib-Glu-Gly-Thr-Phe(2-F)-Thr-Ser-Asp-Ala-Bip NH2	429	6.37	Snx2_ala_10_mir	-219.59	-31.21	-188.38	638.72	244.61	394.11	-2048.43	-1629.30	-2.07	-0.19	136.63	7.00	21.00	11.43	0.00	0.00	0.00	0.00	3.00	2.68	150.74	31.00	-16538.54	-2073.29	444212.79	14308.79
RXL-3009	H His-Aib-Glu-Gly-Thr-Phe(2-F)-Thr-Ser-Asp-Bip-Ala NH2	10000	5.00	Snx2_ala_11_mir	-222.68	-37.26	-185.42	618.53	233.31	385.22	-1984.78	-1588.94	-2.02	-0.21	136.63	7.00	24.00	13.56	0.00	0.00	0.00	0.00	4.00	3.68	153.87	35.00	-14151.54	-1941.74	487272.14	19515.34
Res 1 101	His-Aib-Glu-Gly-Thr-aMePhe(2-F)-Thr-Ser-Asp-Bip-Bip	0.1	10.00	Snx2_aib_2_min	-277.63	-32.86	-244.76	732.82	264.12	468.20	-2326.12	-1871.43	-2.02	0.34	136.63	7.00	35.00	21.33	0.00	0.00	0.00	0.00	4.00	3.32	161.28	46.00	-20041.65	-3172.30	406406.32	18096.64
RXL-3030	Aib-Aib-Glu-Gly-Thr-aMePhe(2-F)-Thr-Ser-Asp-Bip-Bip	2.66	8.58	Snx2_aib_1_min	-275.36	-22.27	-253.09	696.29	248.97	447.32	-2296.83	-1875.89	-2.03	0.11	120.00	6.00	36.00	22.01	0.00	0.00	0.00	0.00	4.00	3.32	145.33	46.00	-18483.57	-2608.00	432983.84	19294.17
RXL-3031	His-Aib-Aib-Gly-Thr-aMePhe(2-F)-Thr-Ser-Asp-Bip-Bip	4.87	8.31	Snx2_aib_3_min	-266.35	-21.00	-245.35	692.75	241.62	451.13	-2284.22	-1857.82	-2.04	0.35	115.90	6.00	35.00	21.33	0.00	0.00	0.00	0.00	4.00	3.32	140.55	45.00	-19717.01	-3047.41	407195.83	17868.92
RXL-3032	His-Aib-Glu-Aib-Thr-aMePhe(2-F)-Thr-Ser-Asp-Bip-Bip	15.8	7.80	Snx2_aib_4_min	-252.19	-33.27	-218.92	743.42	267.43	475.99	-2349.90	-1858.67	-2.04	0.94	136.63	7.00	34.00	20.64	0.00	0.00	0.00	0.00	4.00	3.32	160.59	45.00	-20049.46	-3214.98	406661.99	18210.49
RXL-3033	His-Aib-Glu-Gly-Aib-aMePhe(2-F)-Thr-Ser-Asp-Bip-Bip	7.33	8.13	Snx2_aib_5_min	-283.73	-31.07	-252.66	731.39	264.60	466.80	-2321.25	-1873.59	-2.03	0.58	136.63	7.00	37.00	22.47	0.00	0.00	0.00	0.00	4.00	3.32	162.42	48.00	-19967.85	-3136.49	406355.18	17948.96
RXL-3028	His-Aib-Glu-Gly-Thr-Aib-Thr-Ser-Asp-Bip-Bip	104	6.98	Snx2_aib_6_min	-274.55	-33.08	-241.47	695.07	257.03	438.04	-2233.90	-1813.39	-1.42	-0.05	112.72	6.00	31.00	17.15	0.00	0.00	0.00	0.00	0.00	0.00	129.87	37.00	-20113.29	-2773.67	391836.61	16193.91
RXL-3034	His-Aib-Glu-Gly-Thr-aMePhe(2-F)-Aib-Ser-Asp-Bip-Bip	91.4	7.04	Snx2_aib_7_min	-274.59	-33.00	-241.58	715.29	260.76	454.53	-2310.60	-1869.90	-2.04	0.63	136.63	7.00	32.00	19.54	0.00	0.00	0.00	0.00	4.00	3.32	159.49	43.00	-19997.04	-3157.29	406232.33	17822.46
RXL-3035	His-Aib-Glu-Gly-Thr-aMePhe(2-F)-Thr-Aib-Asp-Bip-Bip	179	6.80	Snx2_aib_8_min	-209.66	-30.87	-178.80	727.72	259.13	468.59	-2327.36	-1809.30	-2.04	0.70	136.63	7.00	35.00	21.34	0.00	0.00	0.00	0.00	4.00	3.32	161.29	46.00	-20071.12	-3224.99	406436.57	18002.49
RXL-3036	His-Aib-Glu-Gly-Thr-aMePhe(2-F)-Thr-Ser-Aib-Bip-Bip	91.6	7.04	Snx2_aib_9_min	-264.65	-17.04	-247.61	704.17	237.73	466.44	-2327.34	-1887.82	-2.05	0.48	92.08	5.00	35.00	21.33	0.00	0.00	0.00	0.00	4.00	3.32	116.73	44.00	-19960.32	-3132.34	406498.76	18121.95
RXL-3051	His-Aib-Glu-Gly-Thr-aMePhe(2-F)-Thr-Ser-Asp-Aib-Bip	10000	5.00	Snx2_aib_10_mir	-192.99	-31.81	-161.19	647.84	245.52	402.32	-2060.10	-1605.26	-2.09	0.44	136.63	7.00	24.00	14.58	0.00	0.00	0.00	0.00	3.00	2.32	153.53	34.00	-16419.33	-1992.49	446122.30	14258.84
RXL-3052	His-Aib-Glu-Gly-Thr-aMePhe(2-F)-Thr-Ser-Asp-Bip-Aib	8.3	8.08	Snx2_aib_11_mir	-228.64	-32.57	-196.06	678.99	230.67	398.37	-1989.55	-1589.19	-2.03	0.34	136.63	7.00	27.00	16.35	0.00	0.00	0.00	0.00	4.00	3.32	156.30	38.00	-14180.98	-1938.68	487165.37	19253.73

Supplementary Tables S7-S11. 5NX2-based reference and QSAR models and Y-randomization results for the QSAR3 model.

Reference	NOVA2 Binding Energy QSAR Model					
**Peptide**	**Peptide Structure (N-Terminus, Amino Acid Sequence, C-T**	**cAMP EC5**	**pEC50**	**Molecules|**	**pEC50**	**BE**
Res 1-100	His-Aib-Glu-Gly-Thr-Phe-Thr-Ser-Asp-Bip-Bip	2.3	8.638272	5nx2_ha_0_min	8.638272	-1872.89
RXL-3000	His-Aib-Glu-Gly-Thr-Phe(2-F)-Thr-Ser-Asp-Bip-Bip	0.12	9.920819	5nx2_ha_1_min	9.920819	-1879.17
RXL-3011	His-Aib-Glu-Gly-Thr-Phe(2-Cl)-Thr-Ser-Asp-Bip-Bip	0.59	9.229148	5nx2_ha_2_min	9.229148	-1880.49
RXL-3012	His-Aib-Glu-Gly-Thr-Phe(2-CF3)-Thr-Ser-Asp-Bip-Bip	11.7	7.931814	5nx2_ha_3_min	7.931814	-1855.21
RXL-3013	His-Aib-Glu-Gly-Thr-Phe(2-CH3)-Thr-Ser-Asp-Bip-Bip	1.54	8.812479	5nx2_ha_4_min	8.812479	-1874.56
RXL-3014	His-Aib-Glu-Gly-Thr-Phe(2-NO2)-Thr-Ser-Asp-Bip-Bip	1.13	8.946922	5nx2_ha_5_mi	8.946922	-1875.99
RXL-3015	His-Aib-Glu-Gly-Thr-Phe(2-CN)-Thr-Ser-Asp-Bip-Bip	9.05	8.043351	5nx2_ha_6_min	8.043351	-1802.62
RXL-3016	His-Aib-Glu-Gly-Thr-Phe(2,6-F)-Thr-Ser-Asp-Bip-Bip	0.16	9.79588	5nx2_ha_7_min	9.79588	-1881.2
RXL-3017	His-Aib-Glu-Gly-Thr-Phe(3,4,5-F)-Thr-Ser-Asp-Bip-Bip	10.8	7.966576	5nx2_ha_8_min	7.966576	-1855.22
RXL-3001	H Ala-Aib-Glu-Gly-Thr-Phe(2-F)-Thr-Ser-Asp-Bip-Bip NH2	13.7	7.863279	5nx2_ala_1_min	7.863279	-1884.39
RXL-3002	H His-Ala-Glu-Gly-Thr-Phe(2-F)-Thr-Ser-Asp-Bip-Bip NH2	0.12	9.920819	5nx2_ala_2_min	9.920819	-1868.81
RXL-3003	H His-Aib-Ala-Gly-Thr-Phe(2-F)-Thr-Ser-Asp-Bip-Bip NH2	3.4	8.468521	5nx2_ala_3_min	8.468521	-1856.43
RXL-3037	H His-Aib-Glu-Ala-Thr-Phe(2F)-Thr-Ser-Asp-Bip-Bip NH2	347	6.459671	5nx2_ala_4_min	6.459671	-1788.75
RXL-3004	H His-Aib-Glu-Gly-Ala-Phe(2-F)-Thr-Ser-Asp-Bip-Bip NH2	6.88	8.162412	5nx2_ala_5_min	8.162412	-1871.34
RXL-3038	H His-Aib-Glu-Gly-Thr-Ala-Thr-Ser-Asp-Bip-Bip NH2 2,80	2800	5.552842	5nx2_ala_6_min	5.552842	-1847.47
RXL-3005	H His-Aib-Glu-Gly-Thr-Phe(2-F)-Ala-Ser-Asp-Bip-Bip NH2	21.2	7.673664	5nx2_ala_7_min	7.673664	-1868.68
RXL-3006	H His-Aib-Glu-Gly-Thr-Phe(2-F)-Thr-Ala-Asp-Bip-Bip NH2	1.91	8.718967	5nx2_ala_8_min	8.718967	-1877.22
RXL-3007	H His-Aib-Glu-Gly-Thr-Phe(2-F)-Thr-Ser-Ala-Bip-Bip NH2	4.74	8.324222	5nx2_ala_9_min	8.324222	-1878.88
RXL-3008	H His-Aib-Glu-Gly-Thr-Phe(2-F)-Thr-Ser-Asp-Ala-Bip NH2	429	6.367543	5nx2_ala_10_min	6.367543	-1629.3
RXL-3009	H His-Aib-Glu-Gly-Thr-Phe(2-F)-Thr-Ser-Asp-Bip-Ala NH2	10000	5	5nx2_ala_11_min	5	-1588.94
Res 1-101	His-Aib-Glu-Gly-Thr-aMePhe(2-F)-Thr-Ser-Asp-Bip-Bip	0.1	10	5nx2_aib_2_min	10	-1871.43
RXL-3030	Aib-Aib-Glu-Gly-Thr-aMePhe(2-F)-Thr-Ser-Asp-Bip-Bip	2.66	8.575118	5nx2_aib_1_min	8.575118	-1875.89
RXL-3031	His-Aib-Aib-Gly-Thr-aMePhe(2-F)-Thr-Ser-Asp-Bip-Bip	4.87	8.312471	5nx2_aib_3_min	8.312471	-1857.82
RXL-3032	His-Aib-Glu-Aib-Thr-aMePhe(2-F)-Thr-Ser-Asp-Bip-Bip	15.8	7.801343	5nx2_aib_4_min	7.801343	-1858.67
RXL-3033	His-Aib-Glu-Gly-Aib-aMePhe(2-F)-Thr-Ser-Asp-Bip-Bip	7.33	8.134896	5nx2_aib_5_min	8.134896	-1873.59
RXL-3028	His-Aib-Glu-Gly-Thr-Aib-Thr-Ser-Asp-Bip-Bip	104	6.982967	5nx2_aib_6_min	6.982967	-1813.39
RXL-3034	His-Aib-Glu-Gly-Thr-aMePhe(2-F)-Aib-Ser-Asp-Bip-Bip	91.4	7.039054	5nx2_aib_7_min	7.039054	-1869.9
RXL-3035	His-Aib-Glu-Gly-Thr-aMePhe(2-F)-Thr-Aib-Asp-Bip-Bip	129	6.88941	5nx2_aib_8_min	6.88941	-1809.3
RXL-3036	His-Aib-Glu-Gly-Thr-aMePhe(2-F)-Thr-Ser-Aib-Bip-Bip	91.6	7.038105	5nx2_aib_9_min	7.038105	-1887.82
RXL-3051	His-Aib-Glu-Gly-Thr-aMePhe(2-F)-Thr-Ser-Asp-Aib-Bip	10000	5	5nx2_aib_10_min	5	-1605.26
RXL-3052	His-Aib-Glu-Gly-Thr-aMePhe(2-F)-Thr-Ser-Asp-Bip-Aib	8.3	8.080922	5nx2_aib_11_min	8.080922	-1589.19

Regression Analysis									
OVERALL FIT									
Multiple R	0.623241607		AIC	5.110079				Q^2	0.244278
R Square	0.388430101		AICc	5.998968					
Adjusted R Square	0.367341483		SBC	7.978054					
Standard Error	1.05258621								
Observations	31								
ANOVA				Alpha	0.05				
	df	*SS*	*MS*	*F*	*p-value*	*sig*			
Regression	1	20.40704517	20.40705	18.41895	0.00018	yes			
Residual	29	32.13019418	1.107938						
Total	30	52.53723934							
	coeff	*std err*	*t stat*	*p-value*	*lower*	*upper*			
Intercept	-8.580118459	3.850263428	-2.22845	0.033765	-16.4548	-0.70545			
BE	-0.009031541	0.002104406	-4.29173	0.00018	-0.01334	-0.00473			

QSAR 1	Interface Contacts QSAR Model					
**Peptide**	**Peptide Structure (N-Terminus, Amino Acid Sequence, C-T**	**cAMP EC5**	**pEC50**	**Molecules|**	**pEC50**	**Interaction Count**
Res 1-100	His-Aib-Glu-Gly-Thr-Phe-Thr-Ser-Asp-Bip-Bip	2.3	8.638272	5nx2_ha_0_min	8.638272	46
RXL-3000	His-Aib-Glu-Gly-Thr-Phe(2-F)-Thr-Ser-Asp-Bip-Bip	0.12	9.920819	5nx2_ha_1_min	9.920819	44
RXL-3011	His-Aib-Glu-Gly-Thr-Phe(2-Cl)-Thr-Ser-Asp-Bip-Bip	0.59	9.229148	5nx2_ha_2_min	9.229148	46
RXL-3012	His-Aib-Glu-Gly-Thr-Phe(2-CF3)-Thr-Ser-Asp-Bip-Bip	11.7	7.931814	5nx2_ha_3_min	7.931814	47
RXL-3013	His-Aib-Glu-Gly-Thr-Phe(2-CH3)-Thr-Ser-Asp-Bip-Bip	1.54	8.812479	5nx2_ha_4_min	8.812479	50
RXL-3014	His-Aib-Glu-Gly-Thr-Phe(2-NO2)-Thr-Ser-Asp-Bip-Bip	1.13	8.946922	5nx2_ha_5_mi	8.946922	43
RXL-3015	His-Aib-Glu-Gly-Thr-Phe(2-CN)-Thr-Ser-Asp-Bip-Bip	9.05	8.043351	5nx2_ha_6_min	8.043351	41
RXL-3039	His-Aib-Glu-Gly-Thr-Phe(2,6-F)-Thr-Ser-Asp-Bip-Bip	0.16	9.79588	5nx2_ha_7_min	9.79588	44
RXL-3017	His-Aib-Glu-Gly-Thr-Phe(3,4,5-F)-Thr-Ser-Asp-Bip-Bip	10.8	7.966576	5nx2_ha_8_min	7.966576	39
RXL-3001	H Ala-Aib-Glu-Gly-Thr-Phe(2-F)-Thr-Ser-Asp-Bip-Bip NH2	13.7	7.863279	5nx2_ala_1_min	7.863279	43
RXL-3002	H His-Ala-Glu-Gly-Thr-Phe(2-F)-Thr-Ser-Asp-Bip-Bip NH2	0.12	9.920819	5nx2_ala_2_min	9.920819	44
RXL-3003	H His-Aib-Ala-Gly-Thr-Phe(2-F)-Thr-Ser-Asp-Bip-Bip NH2	3.4	8.468521	5nx2_ala_3_min	8.468521	43
RXL-3037	H His-Aib-Glu-Ala-Thr-Phe(2F)-Thr-Ser-Asp-Bip-Bip NH2	347	6.459671	5nx2_ala_4_min	6.459671	43
RXL-3004	H His-Aib-Glu-Gly-Ala-Phe(2-F)-Thr-Ser-Asp-Bip-Bip NH2	6.88	8.162412	5nx2_ala_5_min	8.162412	44
RXL-3038	H His-Aib-Glu-Gly-Thr-Ala-Thr-Ser-Asp-Bip-Bip NH2 2,80	2800	5.552842	5nx2_ala_6_min	5.552842	38
RXL-3005	H His-Aib-Glu-Gly-Thr-Phe(2-F)-Ala-Ser-Asp-Bip-Bip NH2	21.2	7.673664	5nx2_ala_7_min	7.673664	41
RXL-3006	H His-Aib-Glu-Gly-Thr-Phe(2-F)-Thr-Ala-Asp-Bip-Bip NH2	1.91	8.718967	5nx2_ala_8_min	8.718967	44
RXL-3007	H His-Aib-Glu-Gly-Thr-Phe(2-F)-Thr-Ser-Ala-Bip-Bip NH2	4.74	8.324222	5nx2_ala_9_min	8.324222	43
RXL-3008	H His-Aib-Glu-Gly-Thr-Phe(2-F)-Thr-Ser-Asp-Ala-Bip NH2	429	6.367543	5nx2_ala_10_min	6.367543	31
RXL-3009	H His-Aib-Glu-Gly-Thr-Phe(2-F)-Thr-Ser-Asp-Bip-Ala NH2	10000	5	5nx2_ala_11_min	5	35
Res 1-101	His-Aib-Glu-Gly-Thr-aMePhe(2-F)-Thr-Ser-Asp-Bip-Bip	0.1	10	5nx2_aib_2_min	10	46
RXL-3030	Aib-Aib-Glu-Gly-Thr-aMePhe(2-F)-Thr-Ser-Asp-Bip-Bip	2.66	8.575118	5nx2_aib_1_min	8.575118	46
RXL-3031	His-Aib-Aib-Gly-Thr-aMePhe(2-F)-Thr-Ser-Asp-Bip-Bip	4.87	8.312471	5nx2_aib_3_min	8.312471	45
RXL-3032	His-Aib-Glu-Aib-Thr-aMePhe(2-F)-Thr-Ser-Asp-Bip-Bip	15.8	7.801343	5nx2_aib_4_min	7.801343	45
RXL-3033	His-Aib-Glu-Gly-Aib-aMePhe(2-F)-Thr-Ser-Asp-Bip-Bip	7.33	8.134896	5nx2_aib_5_min	8.134896	48
RXL-3028	His-Aib-Glu-Gly-Thr-Aib-Thr-Ser-Asp-Bip-Bip	104	6.982967	5nx2_aib_6_min	6.982967	37
RXL-3034	His-Aib-Glu-Gly-Thr-aMePhe(2-F)-Aib-Ser-Asp-Bip-Bip	91.4	7.039054	5nx2_aib_7_min	7.039054	43
RXL-3035	His-Aib-Glu-Gly-Thr-aMePhe(2-F)-Thr-Aib-Asp-Bip-Bip	129	6.88941	5nx2_aib_8_min	6.88941	46
RXL-3036	His-Aib-Glu-Gly-Thr-aMePhe(2-F)-Thr-Ser-Aib-Bip-Bip	91.6	7.038105	5nx2_aib_9_min	7.038105	44
RXL-3051	His-Aib-Glu-Gly-Thr-aMePhe(2-F)-Thr-Ser-Asp-Aib-Bip	10000	5	5nx2_aib_10_min	5	34
RXL-3052	His-Aib-Glu-Gly-Thr-aMePhe(2-F)-Thr-Ser-Asp-Bip-Aib	8.3	8.080922	5nx2_aib_11_min	8.080922	38

Regression Analysis									
OVERALL FIT									
Multiple R	0.652137513		AIC	3.183364				Q^2	0.337637
R Square	0.425283336		AICc	4.072253					
Adjusted R Square	0.40546552		SBC	6.051339					
Standard Error	1.020379019								
Observations	31								
ANOVA				Alpha	0.05				
	df	*SS*	*MS*	*F*	*p-value*	*sig*			
Regression	1	22.34321243	22.34321	21.45965	7.04E-05	yes			
Residual	29	30.19402691	1.041173						
Total	30	52.53723934							
	coeff	*std err*	*t stat*	*p-value*	*lower*	*upper*			
Intercept	-0.716920819	1.874333363	-0.38249	0.704884	-4.55036	3.116521			
Interaction Count	0.202782764	0.043774354	4.632456	7.04E-05	0.113254	0.292311			

QSAR 2	YASARA Quality QSAR Model					
**Peptide**	**Peptide Structure (N-Terminus, Amino Acid Sequence, C-T**	**cAMP EC5**	**pEC50**	**Molecules|**	**pEC50**	**BindPack1D|**
Res 1-100	His-Aib-Glu-Gly-Thr-Phe-Thr-Ser-Asp-Bip-Bip	2.3	8.638272	5nx2_ha_0_min	8.638272	-3206.45
RXL-3000	His-Aib-Glu-Gly-Thr-Phe(2-F)-Thr-Ser-Asp-Bip-Bip	0.12	9.920819	5nx2_ha_1_min	9.920819	-3102.33
RXL-3011	His-Aib-Glu-Gly-Thr-Phe(2-Cl)-Thr-Ser-Asp-Bip-Bip	0.59	9.229148	5nx2_ha_2_min	9.229148	-3183.44
RXL-3012	His-Aib-Glu-Gly-Thr-Phe(2-CF3)-Thr-Ser-Asp-Bip-Bip	11.7	7.931814	5nx2_ha_3_min	7.931814	-3284.76
RXL-3013	His-Aib-Glu-Gly-Thr-Phe(2-CH3)-Thr-Ser-Asp-Bip-Bip	1.54	8.812479	5nx2_ha_4_min	8.812479	-3206.56
RXL-3014	His-Aib-Glu-Gly-Thr-Phe(2-NO2)-Thr-Ser-Asp-Bip-Bip	1.13	8.946922	5nx2_ha_5_mi	8.946922	-3443.99
RXL-3015	His-Aib-Glu-Gly-Thr-Phe(2-CN)-Thr-Ser-Asp-Bip-Bip	9.05	8.043351	5nx2_ha_6_min	8.043351	-3214.15
RXL-3039	His-Aib-Glu-Gly-Thr-Phe(2,6-F)-Thr-Ser-Asp-Bip-Bip	0.16	9.79588	5nx2_ha_7_min	9.79588	-2944.29
RXL-3017	His-Aib-Glu-Gly-Thr-Phe(3,4,5-F)-Thr-Ser-Asp-Bip-Bip	10.8	7.966576	5nx2_ha_8_min	7.966576	-2916.81
RXL-3001	H Ala-Aib-Glu-Gly-Thr-Phe(2-F)-Thr-Ser-Asp-Bip-Bip NH2	13.7	7.863279	5nx2_ala_1_min	7.863279	-2459.28
RXL-3002	H His-Ala-Glu-Gly-Thr-Phe(2-F)-Thr-Ser-Asp-Bip-Bip NH2	0.12	9.920819	5nx2_ala_2_min	9.920819	-3113.45
RXL-3003	H His-Aib-Ala-Gly-Thr-Phe(2-F)-Thr-Ser-Asp-Bip-Bip NH2	3.4	8.468521	5nx2_ala_3_min	8.468521	-2999.63
RXL-3037	H His-Aib-Glu-Ala-Thr-Phe(2F)-Thr-Ser-Asp-Bip-Bip NH2	347	6.459671	5nx2_ala_4_min	6.459671	-3084.19
RXL-3004	H His-Aib-Glu-Gly-Ala-Phe(2-F)-Thr-Ser-Asp-Bip-Bip NH2	6.88	8.162412	5nx2_ala_5_min	8.162412	-3007.6
RXL-3038	H His-Aib-Glu-Gly-Thr-Ala-Thr-Ser-Asp-Bip-Bip NH2 2,80	2800	5.552842	5nx2_ala_6_min	5.552842	-2586.93
RXL-3005	H His-Aib-Glu-Gly-Thr-Phe(2-F)-Ala-Ser-Asp-Bip-Bip NH2	21.2	7.673664	5nx2_ala_7_min	7.673664	-3007.76
RXL-3006	H His-Aib-Glu-Gly-Thr-Phe(2-F)-Thr-Ala-Asp-Bip-Bip NH2	1.91	8.718967	5nx2_ala_8_min	8.718967	-3077.13
RXL-3007	H His-Aib-Glu-Gly-Thr-Phe(2-F)-Thr-Ser-Ala-Bip-Bip NH2	4.74	8.324222	5nx2_ala_9_min	8.324222	-3036.43
RXL-3008	H His-Aib-Glu-Gly-Thr-Phe(2-F)-Thr-Ser-Asp-Ala-Bip NH2	429	6.367543	5nx2_ala_10_min	6.367543	-2073.29
RXL-3009	H His-Aib-Glu-Gly-Thr-Phe(2-F)-Thr-Ser-Asp-Bip-Ala NH2	10000	5	5nx2_ala_11_min	5	-1941.74
Res 1-101	His-Aib-Glu-Gly-Thr-aMePhe(2-F)-Thr-Ser-Asp-Bip-Bip	0.1	10	5nx2_aib_2_min	10	-3172.3
RXL-3030	Aib-Aib-Glu-Gly-Thr-aMePhe(2-F)-Thr-Ser-Asp-Bip-Bip	2.66	8.575118	5nx2_aib_1_min	8.575118	-2608
RXL-3031	His-Aib-Aib-Gly-Thr-aMePhe(2-F)-Thr-Ser-Asp-Bip-Bip	4.87	8.312471	5nx2_aib_3_min	8.312471	-3047.41
RXL-3032	His-Aib-Glu-Aib-Thr-aMePhe(2-F)-Thr-Ser-Asp-Bip-Bip	15.8	7.801343	5nx2_aib_4_min	7.801343	-3214.98
RXL-3033	His-Aib-Glu-Gly-Aib-aMePhe(2-F)-Thr-Ser-Asp-Bip-Bip	7.33	8.134896	5nx2_aib_5_min	8.134896	-3136.49
RXL-3028	His-Aib-Glu-Gly-Thr-Aib-Thr-Ser-Asp-Bip-Bip	104	6.982967	5nx2_aib_6_min	6.982967	-2773.67
RXL-3034	His-Aib-Glu-Gly-Thr-aMePhe(2-F)-Aib-Ser-Asp-Bip-Bip	91.4	7.039054	5nx2_aib_7_min	7.039054	-3157.29
RXL-3035	His-Aib-Glu-Gly-Thr-aMePhe(2-F)-Thr-Aib-Asp-Bip-Bip	129	6.88941	5nx2_aib_8_min	6.88941	-3224.99
RXL-3036	His-Aib-Glu-Gly-Thr-aMePhe(2-F)-Thr-Ser-Aib-Bip-Bip	91.6	7.038105	5nx2_aib_9_min	7.038105	-3132.34
RXL-3051	His-Aib-Glu-Gly-Thr-aMePhe(2-F)-Thr-Ser-Asp-Aib-Bip	10000	5	5nx2_aib_10_min	5	-1992.49
RXL-3052	His-Aib-Glu-Gly-Thr-aMePhe(2-F)-Thr-Ser-Asp-Bip-Aib	8.3	8.080922	5nx2_aib_11_min	8.080922	-1938.68

Regression Analysis									
OVERALL FIT									
Multiple R	0.573262083		AIC	8.002132				Q^2	0.202243
R Square	0.328629415		AICc	8.891021					
Adjusted R Square	0.305478705		SBC	10.87011					
Standard Error	1.102848304								
Observations	31								
ANOVA				Alpha	0.05				
	df	*SS*	*MS*	*F*	*p-value*	*sig*			
Regression	1	17.26528225	17.26528	14.19522	0.000749	yes			
Residual	29	35.2719571	1.216274						
Total	30	52.53723934							
	coeff	*std err*	*t stat*	*p-value*	*lower*	*upper*			
Intercept	2.633132912	1.41825099	1.856606	0.073553	-0.26752	5.533782			
BindPack1D|	-0.001816662	0.000482173	-3.76765	0.000749	-0.0028	-0.00083			

QSAR 3	NOVA2 Terms QSAR Model						
**Peptide**	**Peptide Structure (N-Terminus, Amino Acid Sequence, C-T**	**cAMP EC5**	**pEC50**	**Molecules**	**pEC50**	**bindpot**	**bindsolvvdw**
Res 1-100	His-Aib-Glu-Gly-Thr-Phe-Thr-Ser-Asp-Bip-Bip	2.3	8.638272	5nx2_ha_0_min	8.638272	-263.21	468.53
RXL-3000	His-Aib-Glu-Gly-Thr-Phe(2-F)-Thr-Ser-Asp-Bip-Bip	0.12	9.920819	5nx2_ha_1_min	9.920819	-275.38	469.49
RXL-3011	His-Aib-Glu-Gly-Thr-Phe(2-Cl)-Thr-Ser-Asp-Bip-Bip	0.59	9.229148	5nx2_ha_2_min	9.229148	-277.51	473.5
RXL-3012	His-Aib-Glu-Gly-Thr-Phe(2-CF3)-Thr-Ser-Asp-Bip-Bip	11.7	7.931814	5nx2_ha_3_min	7.931814	-263.04	474.01
RXL-3013	His-Aib-Glu-Gly-Thr-Phe(2-CH3)-Thr-Ser-Asp-Bip-Bip	1.54	8.812479	5nx2_ha_4_min	8.812479	-275.36	472.9
RXL-3014	His-Aib-Glu-Gly-Thr-Phe(2-NO2)-Thr-Ser-Asp-Bip-Bip	1.13	8.946922	5nx2_ha_5_mi	8.946922	-275.54	471.74
RXL-3015	His-Aib-Glu-Gly-Thr-Phe(2-CN)-Thr-Ser-Asp-Bip-Bip	9.05	8.043351	5nx2_ha_6_min	8.043351	-256.17	465.7
RXL-3039	His-Aib-Glu-Gly-Thr-Phe(2,6-F)-Thr-Ser-Asp-Bip-Bip	0.16	9.79588	5nx2_ha_7_min	9.79588	-277.86	468.77
RXL-3017	His-Aib-Glu-Gly-Thr-Phe(3,4,5-F)-Thr-Ser-Asp-Bip-Bip	10.8	7.966576	5nx2_ha_8_min	7.966576	-258.79	470.73
RXL-3001	H Ala-Aib-Glu-Gly-Thr-Phe(2-F)-Thr-Ser-Asp-Bip-Bip NH2	13.7	7.863279	5nx2_ala_1_min	7.863279	-260.53	427.75
RXL-3002	H His-Ala-Glu-Gly-Thr-Phe(2-F)-Thr-Ser-Asp-Bip-Bip NH2	0.12	9.920819	5nx2_ala_2_min	9.920819	-267.06	458.66
RXL-3003	H His-Aib-Ala-Gly-Thr-Phe(2-F)-Thr-Ser-Asp-Bip-Bip NH2	3.4	8.468521	5nx2_ala_3_min	8.468521	-254.2	439.88
RXL-3037	H His-Aib-Glu-Ala-Thr-Phe(2F)-Thr-Ser-Asp-Bip-Bip NH2	347	6.459671	5nx2_ala_4_min	6.459671	-188.52	471.17
RXL-3004	H His-Aib-Glu-Gly-Ala-Phe(2-F)-Thr-Ser-Asp-Bip-Bip NH2	6.88	8.162412	5nx2_ala_5_min	8.162412	-277.26	461.16
RXL-3038	H His-Aib-Glu-Gly-Thr-Ala-Thr-Ser-Asp-Bip-Bip NH2 2,80	2800	5.552842	5nx2_ala_6_min	5.552842	-244.31	401.09
RXL-3005	H His-Aib-Glu-Gly-Thr-Phe(2-F)-Ala-Ser-Asp-Bip-Bip NH2	21.2	7.673664	5nx2_ala_7_min	7.673664	-270.36	455.5
RXL-3006	H His-Aib-Glu-Gly-Thr-Phe(2-F)-Thr-Ala-Asp-Bip-Bip NH2	1.91	8.718967	5nx2_ala_8_min	8.718967	-275.06	466.66
RXL-3007	H His-Aib-Glu-Gly-Thr-Phe(2-F)-Thr-Ser-Ala-Bip-Bip NH2	4.74	8.324222	5nx2_ala_9_min	8.324222	-258.37	466.39
RXL-3008	H His-Aib-Glu-Gly-Thr-Phe(2-F)-Thr-Ser-Asp-Ala-Bip NH2	429	6.367543	5nx2_ala_10_min	6.367543	-219.59	394.11
RXL-3009	H His-Aib-Glu-Gly-Thr-Phe(2-F)-Thr-Ser-Asp-Bip-Ala NH2	10000	5	5nx2_ala_11_min	5	-222.68	385.22
Res 1-101	His-Aib-Glu-Gly-Thr-aMePhe(2-F)-Thr-Ser-Asp-Bip-Bip	0.1	10	5nx2_aib_2_min	10	-277.63	468.2
RXL-3030	Aib-Aib-Glu-Gly-Thr-aMePhe(2-F)-Thr-Ser-Asp-Bip-Bip	2.66	8.575118	5nx2_aib_1_min	8.575118	-275.36	447.32
RXL-3031	His-Aib-Aib-Gly-Thr-aMePhe(2-F)-Thr-Ser-Asp-Bip-Bip	4.87	8.312471	5nx2_aib_3_min	8.312471	-266.35	451.13
RXL-3032	His-Aib-Glu-Aib-Thr-aMePhe(2-F)-Thr-Ser-Asp-Bip-Bip	15.8	7.801343	5nx2_aib_4_min	7.801343	-252.19	475.99
RXL-3033	His-Aib-Glu-Gly-Aib-aMePhe(2-F)-Thr-Ser-Asp-Bip-Bip	7.33	8.134896	5nx2_aib_5_min	8.134896	-283.73	466.8
RXL-3028	His-Aib-Glu-Gly-Thr-Aib-Thr-Ser-Asp-Bip-Bip	104	6.982967	5nx2_aib_6_min	6.982967	-274.55	438.04
RXL-3034	His-Aib-Glu-Gly-Thr-aMePhe(2-F)-Aib-Ser-Asp-Bip-Bip	91.4	7.039054	5nx2_aib_7_min	7.039054	-274.59	454.53
RXL-3035	His-Aib-Glu-Gly-Thr-aMePhe(2-F)-Thr-Aib-Asp-Bip-Bip	129	6.88941	5nx2_aib_8_min	6.88941	-209.66	468.59
RXL-3036	His-Aib-Glu-Gly-Thr-aMePhe(2-F)-Thr-Ser-Aib-Bip-Bip	91.6	7.038105	5nx2_aib_9_min	7.038105	-264.65	466.44
RXL-3051	His-Aib-Glu-Gly-Thr-aMePhe(2-F)-Thr-Ser-Asp-Aib-Bip	10000	5	5nx2_aib_10_min	5	-192.99	402.32
RXL-3052	His-Aib-Glu-Gly-Thr-aMePhe(2-F)-Thr-Ser-Asp-Bip-Aib	8.3	8.080922	5nx2_aib_11_min	8.080922	-228.64	398.37

Regression Analysis								
OVERALL FIT								
Multiple R	0.781915425		AIC	-6.9471			Q^2	0.529874
R Square	0.611391732		AICc	-5.40864				
Adjusted R Square	0.583633998		SBC	-2.64514				
Standard Error	0.853907104							
Observations	31							
ANOVA				Alpha	0.05			
	df	*SS*	*MS*	*F*	*p-value*	*sig*		
Regression	2	32.12083	16.06042	22.026	1.79E-06	yes		
Residual	28	20.41641	0.729157					
Total	30	52.53724						
	coeff	*std err*	*t stat*	*p-value*	*lower*	*upper*	*vif*	
Intercept	-7.260140446	2.598996	-2.79344	0.009301	-12.5839	-1.9363383		
bindpot	-0.02634962	0.007063	-3.73085	0.000861	-0.04082	-0.01188247	1.356206204	
bindsolvvdw	0.018637855	0.006653	2.801609	0.00912	0.005011	0.03226499	1.356206204	

**Model**	**R**	**R^2**	**Q^2**
Original (QSAR3)	0.78	0.61	0.53
Random 1	0.21	0.04	-0.28
Random 2	0.18	0.03	-0.21
Random 3	0.06	0.00	-0.20
Random 4	0.30	0.09	-0.15
Random 5	0.13	0.02	-0.15
Random 6	0.26	0.07	-0.10
Random 7	0.50	0.25	0.00
Random 8	0.27	0.07	-0.12
Random 9	0.34	0.12	-0.07
Random 10	0.29	0.08	-0.09
Random 11	0.37	0.13	-0.10
Random 12	0.24	0.06	-0.15
Random 13	0.35	0.12	-0.20
Random 14	0.06	0.00	-0.19
Random 15	0.40	0.16	-0.04
Random 16	0.38	0.15	-0.07
Random 17	0.10	0.01	-0.20
Random 18	0.22	0.05	-0.20
Random 19	0.12	0.01	-0.22
Random 20	0.32	0.10	-0.07
Random 21	0.15	0.02	-0.19
Random 22	0.19	0.03	-0.17
Random 23	0.31	0.09	-0.08
Random 24	0.31	0.10	-0.39
Random 25	0.24	0.06	-0.11
Random 26	0.21	0.05	-0.21
Random 27	0.34	0.12	-0.18
Random 28	0.24	0.06	-0.16
Random 29	0.15	0.02	-0.26
Random 30	0.32	0.10	-0.03
Random 31	0.07	0.01	-0.25
Random 32	0.42	0.18	-0.11
Random 33	0.47	0.22	0.01
Random 34	0.18	0.03	-0.24
Random 35	0.15	0.02	-0.27
Random 36	0.04	0.00	-0.21
Random 37	0.23	0.06	-0.24
Random 38	0.04	0.00	-0.21
Random 39	0.30	0.09	-0.17
Random 40	0.08	0.01	-0.12
Random 41	0.26	0.07	-0.13
Random 42	0.24	0.06	-0.11
Random 43	0.12	0.02	-0.17
Random 44	0.30	0.09	-0.19
Random 45	0.19	0.04	-0.15
Random 46	0.07	0.00	-0.22
Random 47	0.19	0.04	-0.12
Random 48	0.29	0.08	-0.06
Random 49	0.46	0.21	0.09
Random 50	0.20	0.04	-0.20
Random Models Parameters			
Average r :	0.24		
Average r^2 :	0.07		
Average Q^2 :	-0.15		
cRp^2 :	0.58		

QSAR 4	Mixed QSAR Model							
Peptide	Peptide Structure (N-Terminus, Amino Acid Sequence,	cAMP EC5	pEC50	Molecules|	pEC50	bindpot|	bindsolvvdv	CompPack1D|
Res 1-100	His-Aib-Glu-Gly-Thr-Phe-Thr-Ser-Asp-Bip-Bip	2.3	8.638272	5nx2_ha_0_min	8.638272	-263.21	468.53	-20066.82
RXL-3000	His-Aib-Glu-Gly-Thr-Phe(2-F)-Thr-Ser-Asp-Bip-Bip	0.12	9.920819	5nx2_ha_1_min	9.920819	-275.38	469.49	-19945.25
RXL-3011	His-Aib-Glu-Gly-Thr-Phe(2-Cl)-Thr-Ser-Asp-Bip-Bip	0.59	9.229148	5nx2_ha_2_min	9.229148	-277.51	473.5	-20042.97
RXL-3012	His-Aib-Glu-Gly-Thr-Phe(2-CF3)-Thr-Ser-Asp-Bip-Bip	11.7	7.931814	5nx2_ha_3_min	7.931814	-263.04	474.01	-20178.65
RXL-3013	His-Aib-Glu-Gly-Thr-Phe(2-CH3)-Thr-Ser-Asp-Bip-Bip	1.54	8.812479	5nx2_ha_4_min	8.812479	-275.36	472.9	-20081.46
RXL-3014	His-Aib-Glu-Gly-Thr-Phe(2-NO2)-Thr-Ser-Asp-Bip-Bip	1.13	8.946922	5nx2_ha_5_mi	8.946922	-275.54	471.74	-20327.74
RXL-3015	His-Aib-Glu-Gly-Thr-Phe(2-CN)-Thr-Ser-Asp-Bip-Bip	9.05	8.043351	5nx2_ha_6_min	8.043351	-256.17	465.7	-20098.51
RXL-3016	His-Aib-Glu-Gly-Thr-Phe(2,6-F)-Thr-Ser-Asp-Bip-Bip	0.16	9.79588	5nx2_ha_7_min	9.79588	-277.86	468.77	-19757.81
RXL-3017	His-Aib-Glu-Gly-Thr-Phe(3,4,5-F)-Thr-Ser-Asp-Bip-Bip	10.8	7.966576	5nx2_ha_8_min	7.966576	-258.79	470.73	-19689.89
RXL-3001	H Ala-Aib-Glu-Gly-Thr-Phe(2-F)-Thr-Ser-Asp-Bip-Bip NH	13.7	7.863279	5nx2_ala_1_min	7.863279	-260.53	427.75	-17411.16
RXL-3002	H His-Ala-Glu-Gly-Thr-Phe(2-F)-Thr-Ser-Asp-Bip-Bip NH	0.12	9.920819	5nx2_ala_2_min	9.920819	-267.06	458.66	-19948.94
RXL-3003	H His-Aib-Ala-Gly-Thr-Phe(2-F)-Thr-Ser-Asp-Bip-Bip NH	3.4	8.468521	5nx2_ala_3_min	8.468521	-254.2	439.88	-19636.29
RXL-3037	H His-Aib-Glu-Ala-Thr-Phe(2F)-Thr-Ser-Asp-Bip-Bip NH	347	6.459671	5nx2_ala_4_min	6.459671	-188.52	471.17	-19909.34
RXL-3004	H His-Aib-Glu-Gly-Ala-Phe(2-F)-Thr-Ser-Asp-Bip-Bip NH	6.88	8.162412	5nx2_ala_5_min	8.162412	-277.26	461.16	-19807.04
RXL-3038	H His-Aib-Glu-Gly-Thr-Ala-Thr-Ser-Asp-Bip-Bip NH2 2,8	2800	5.552842	5nx2_ala_6_min	5.552842	-244.31	401.09	-19318.44
RXL-3005	H His-Aib-Glu-Gly-Thr-Phe(2-F)-Ala-Ser-Asp-Bip-Bip NH	21.2	7.673664	5nx2_ala_7_min	7.673664	-270.36	455.5	-19812.34
RXL-3006	H His-Aib-Glu-Gly-Thr-Phe(2-F)-Thr-Ala-Asp-Bip-Bip NH	1.91	8.718967	5nx2_ala_8_min	8.718967	-275.06	466.66	-19895.37
RXL-3007	H His-Aib-Glu-Gly-Thr-Phe(2-F)-Thr-Ser-Ala-Bip-Bip NH	4.74	8.324222	5nx2_ala_9_min	8.324222	-258.37	466.39	-19849.2
RXL-3008	H His-Aib-Glu-Gly-Thr-Phe(2-F)-Thr-Ser-Asp-Ala-Bip NH	429	6.367543	5nx2_ala_10_mir	6.367543	-219.59	394.11	-16538.54
RXL-3009	H His-Aib-Glu-Gly-Thr-Phe(2-F)-Thr-Ser-Asp-Bip-Ala NH	10000	5	5nx2_ala_11_mir	5	-222.68	385.22	-14151.54
Res 1-101	His-Aib-Glu-Gly-Thr-aMePhe(2-F)-Thr-Ser-Asp-Bip-Bip	0.1	10	5nx2_aib_2_min	10	-277.63	468.2	-20041.65
RXL-3030	Aib-Aib-Glu-Gly-Thr-aMePhe(2-F)-Thr-Ser-Asp-Bip-Bip	2.66	8.575118	5nx2_aib_1_min	8.575118	-275.36	447.32	-18483.57
RXL-3031	His-Aib-Aib-Gly-Thr-aMePhe(2-F)-Thr-Ser-Asp-Bip-Bip	4.87	8.312471	5nx2_aib_3_min	8.312471	-266.35	451.13	-19717.01
RXL-3032	His-Aib-Glu-Aib-Thr-aMePhe(2-F)-Thr-Ser-Asp-Bip-Bip	15.8	7.801343	5nx2_aib_4_min	7.801343	-252.19	475.99	-20049.46
RXL-3033	His-Aib-Glu-Gly-Aib-aMePhe(2-F)-Thr-Ser-Asp-Bip-Bip	7.33	8.134896	5nx2_aib_5_min	8.134896	-283.73	466.8	-19967.85
RXL-3028	His-Aib-Glu-Gly-Thr-Aib-Thr-Ser-Asp-Bip-Bip	104	6.982967	5nx2_aib_6_min	6.982967	-274.55	438.04	-20113.29
RXL-3034	His-Aib-Glu-Gly-Thr-aMePhe(2-F)-Aib-Ser-Asp-Bip-Bip	91.4	7.039054	5nx2_aib_7_min	7.039054	-274.59	454.53	-19997.04
RXL-3035	His-Aib-Glu-Gly-Thr-aMePhe(2-F)-Thr-Aib-Asp-Bip-Bip	129	6.88941	5nx2_aib_8_min	6.88941	-209.66	468.59	-20071.12
RXL-3036	His-Aib-Glu-Gly-Thr-aMePhe(2-F)-Thr-Ser-Aib-Bip-Bip	91.6	7.038105	5nx2_aib_9_min	7.038105	-264.65	466.44	-19960.32
RXL-3051	His-Aib-Glu-Gly-Thr-aMePhe(2-F)-Thr-Ser-Asp-Aib-Bip	10000	5	5nx2_aib_10_mir	5	-192.99	402.32	-16419.33
RXL-3052	His-Aib-Glu-Gly-Thr-aMePhe(2-F)-Thr-Ser-Asp-Bip-Aib	8.3	8.080922	5nx2_aib_11_mir	8.080922	-228.64	398.37	-14180.98

Regression Analysis

OVERALL FIT						
Multiple R	0.817183	AIC	-9.80788	Q^2	0.540406
R Square	0.667788	AICc	-7.40788		
Adjusted R Squar	0.630876	SBC	-4.07193		
Standard Error	0.804006				
Observations	31				

ANOVA				Alpha	0.05	
	df	*SS*	*MS*	*F*	*p-value*	*sig*
Regression	3	35.08375	11.69458	18.09116	1.2249E-06	yes
Residual	27	17.45349	0.646425			
Total	30	52.53724				

	coeff	*std err*	*t stat*	*p-value*	*lower*	*upper*	*vif*
Intercept	-9.13005	2.598311	-3.51384	0.001576	-14.4613458	-3.798757992	
bindpot|	-0.02946	0.006807	-4.32805	0.000185	-0.0434263	-0.015493688	1.420938
bindsolvvdw|	0.037175	0.010687	3.478634	0.001726	0.01524784	0.059102496	3.947663
CompPack1D|	0.00038	0.000178	2.140921	0.041464	1.5817E-05	0.000744358	4.075996

Supplementary Table S12. All 6X18-based QSAR descriptors.

Peptide	Peptide Structure (N-Terminus, Amino Acid Sequence, C-Termir	cAMP EC5	pEC50	Molecules	bindpo	bindpotcoul	bindpotvdw	bindsolv	bindsolvcoul	bindsolvvdw	bindentropy	bindenergy	CompMQ
Res 1-100	His-Aib-Glu-Gly-Thr-Phe-Thr-Ser-Asp-Bip-Bip	2.30	8.64	6x18_ha_0_min	-214.11	-39.36	-174.75	658.80	252.60	406.19	-2184.80	-1740.11	-1.56
RXL-3000	His-Aib-Glu-Gly-Thr-**Phe(2-F)**-Thr-Ser-Asp-Bip-Bip	0.12	9.92	6x18_ha_1_min	-225.13	-40.48	-184.64	668.60	260.86	407.73	-2186.03	-1742.56	-1.58
RXL-3011	His-Aib-Glu-Gly-Thr-**Phe(2-Cl)**-Thr-Ser-Asp-Bip-Bip	0.59	9.23	6x18_ha_2_min	-221.25	-40.07	-181.17	680.13	259.60	420.54	-2184.15	-1725.26	-1.57
RXL-3012	His-Aib-Glu-Gly-Thr-**Phe(2-CF3)**-Thr-Ser-Asp-Bip-Bip	11.70	7.93	6x18_ha_3_min	-206.64	-41.54	-165.10	688.11	268.14	419.97	-2182.47	-1701.00	-1.57
RXL-3013	His-Aib-Glu-Gly-Thr-**Phe(2-CH3)**-Thr-Ser-Asp-Bip-Bip	1.54	8.81	6x18_ha_4_min	-221.64	-39.09	-182.54	679.95	259.47	420.48	-2183.84	-1725.53	-1.57
RXL-3014	His-Aib-Glu-Gly-Thr-**Phe(2-NO2)**-Thr-Ser-Asp-Bip-Bip	1.13	8.95	6x18_ha_5_mi	-209.31	-39.97	-169.34	682.67	263.02	419.65	-2178.34	-1704.98	-1.58
RXL-3015	His-Aib-Glu-Gly-Thr-**Phe(2-CN)**-Thr-Ser-Asp-Bip-Bip	9.05	8.04	6x18_ha_6_min	-206.27	-24.11	-182.15	694.30	281.58	412.72	-2194.53	-1706.50	-1.56
RXL-3039	His-Aib-Glu-Gly-Thr-**Phe(2,6-F)**-Thr-Ser-Asp-Bip-Bip	0.16	9.80	6x18_ha_7_min	-224.07	-41.73	-182.34	670.49	261.22	409.28	-2188.00	-1741.57	-1.59
RXL-3017	His-Aib-Glu-Gly-Thr-**Phe(3,4,5-F)**-Thr-Ser-Asp-Bip-Bip	10.80	7.97	6x18_ha_8_min	-218.98	-34.84	-184.14	674.16	264.67	409.50	-2188.19	-1733.01	-1.61
RXL-3001	H **Ala**-Aib-Glu-Gly-Thr-Phe(2-F)-Thr-Ser-Asp-Bip-Bip NH2	13.70	7.86	6x18_ala_1_min	-207.37	-33.78	-173.59	601.95	236.06	365.88	-2135.82	-1741.24	-1.60
RXL-3002	H His-**Ala**-Glu-Gly-Thr-Phe(2-F)-Thr-Ser-Asp-Bip-Bip NH2	0.12	9.92	6x18_ala_2_min	-234.99	-42.05	-192.94	666.65	263.54	403.11	-2185.99	-1754.33	-1.57
RXL-3003	H His-Aib-**Ala**-Gly-Thr-Phe(2-F)-Thr-Ser-Asp-Bip-Bip NH2	3.40	8.47	6x18_ala_3_min	-200.59	-23.46	-177.14	623.89	237.94	385.95	-2125.08	-1701.78	-1.59
RXL-3037	H His-Aib-Glu-**Ala**-Thr-Phe(2F)-Thr-Ser-Asp-Bip-Bip NH2	347.00	6.46	6x18_ala_4_min	693.69	-40.52	734.21	668.09	257.48	410.60	-2185.82	-824.04	-1.57
RXL-3004	H His-Aib-Glu-Gly-**Ala**-Phe(2-F)-Thr-Ser-Asp-Bip-Bip NH2	6.88	8.16	6x18_ala_5_min	-220.77	-39.90	-180.86	646.27	255.91	390.36	-2167.75	-1742.24	-1.59
RXL-3038	H His-Aib-Glu-Gly-Thr-**Ala**-Thr-Ser-Asp-Bip-Bip NH2 2,80	2800.00	5.55	6x18_ala_6_min	-193.67	-39.26	-154.41	596.01	244.92	351.09	-2076.57	-1674.23	-1.60
RXL-3005	H His-Aib-Glu-Gly-Thr-Phe(2-F)-**Ala**-Ser-Asp-Bip-Bip NH2	21.20	7.67	6x18_ala_7_min	-221.44	-39.42	-182.02	661.34	260.37	400.97	-2170.43	-1730.52	-1.59
RXL-3006	H His-Aib-Glu-Gly-Thr-Phe(2-F)-Thr-**Ala**-Asp-Bip-Bip NH2	1.91	8.72	6x18_ala_8_min	-221.40	-36.35	-185.05	664.90	258.46	406.44	-2178.90	-1735.39	-1.58
RXL-3007	H His-Aib-Glu-Gly-Thr-Phe(2-F)-Thr-Ser-**Ala**-Bip-Bip NH2	4.74	8.32	6x18_ala_9_min	-207.31	-18.26	-189.05	632.86	227.44	405.41	-2162.23	-1736.69	-1.58
RXL-3008	H His-Aib-Glu-Gly-Thr-Phe(2-F)-Thr-Ser-Asp-**Ala**-Bip NH2	429.00	6.37	6x18_ala_10_min	-195.49	-41.18	-154.31	615.96	246.69	369.27	-1958.45	-1537.98	-1.58
RXL-3009	H His-Aib-Glu-Gly-Thr-Phe(2-F)-Thr-Ser-Asp-Bip-**Ala** NH2	10000.00	5.00	6x18_ala_11_min	-184.29	-41.69	-142.61	542.54	217.02	325.52	-1861.24	-1502.99	-1.59
Res 1-101	His-Aib-Glu-Gly-Thr-**aMePhe(2-F)**-Thr-Ser-Asp-Bip-Bip	0.10	10.00	6x18_aib_2_min	-182.03	-43.68	-138.35	655.05	255.93	399.12	-2175.57	-1702.56	-1.84
RXL-3030	**Aib**-Aib-Glu-Gly-Thr-aMePhe(2-F)-Thr-Ser-Asp-Bip-Bip	2.66	8.58	6x18_aib_1_min	-186.42	-43.78	-142.64	631.98	271.96	360.02	-2189.45	-1743.89	-1.86
RXL-3031	His-Aib-**Aib**-Gly-Thr-aMePhe(2-F)-Thr-Ser-Asp-Bip-Bip	4.87	8.31	6x18_aib_3_min	-163.79	-30.73	-133.06	630.52	238.70	391.82	-2129.05	-1662.32	-1.85
RXL-3032	His-Aib-Glu-**Aib**-Thr-aMePhe(2-F)-Thr-Ser-Asp-Bip-Bip	15.80	7.80	6x18_aib_4_min	-42.40	-39.91	-2.49	660.33	253.93	406.40	-2189.55	-1571.62	-1.85
RXL-3033	His-Aib-Glu-Gly-**Aib**-aMePhe(2-F)-Thr-Ser-Asp-Bip-Bip	7.33	8.13	6x18_aib_5_min	-186.15	-43.11	-143.04	642.51	252.33	390.19	-2171.98	-1715.61	-1.85
RXL-3028	His-Aib-Glu-Gly-Thr-**Aib**-Thr-Ser-Asp-Bip-Bip	104.00	6.98	6x18_aib_6_min	-182.71	-40.85	-141.86	601.40	242.43	358.97	-2073.74	-1655.05	-1.86
RXL-3034	His-Aib-Glu-Gly-Thr-aMePhe(2-F)-**Aib**-Ser-Asp-Bip-Bip	91.40	7.04	6x18_aib_7_min	-180.69	-44.40	-136.29	654.37	257.92	396.45	-2155.55	-1681.87	-1.86
RXL-3035	His-Aib-Glu-Gly-Thr-aMePhe(2-F)-Thr-**Aib**-Asp-Bip-Bip	129.00	6.89	6x18_aib_8_min	-134.22	-34.51	-99.71	635.08	233.80	401.28	-2169.27	-1668.42	-1.86
RXL-3036	His-Aib-Glu-Gly-Thr-aMePhe(2-F)-Thr-Ser-**Aib**-Bip-Bip	91.60	7.04	6x18_aib_9_min	-168.42	-23.42	-145.00	627.77	230.85	396.92	-2163.73	-1704.37	-1.85
RXL-3051	His-Aib-Glu-Gly-Thr-aMePhe(2-F)-Thr-Ser-Asp-**Aib**-Bip	10000.00	5.00	6x18_aib_10_min	-136.48	-44.32	-92.16	618.63	244.94	373.69	-1962.19	-1480.05	-1.85
RXL-3052	His-Aib-Glu-Gly-Thr-aMePhe(2-F)-Thr-Ser-Asp-Bip-**Aib**	8.30	8.08	6x18_aib_11_min	-117.56	-42.38	-75.18	530.38	210.29	320.09	-1856.90	-1444.08	-1.86

Peptide	BindMQ	HB Energy|	HB Total	H Num	H Energy	Cation-ion	Cation-ion Energy	Ion-Ion	Ion-Ion Energy	PiPi Num	PiPi Energy	Energy	Interface Num	CompPack1D	BindPack1D	CompPack3D	BindPack3D
Res 1-100	-0.21	107.25	5.00	43.00	23.78	0.00	0.00	1.00	0.96	10.00	6.33	137.36	58.00	-19797.75	-2223.28	448869.94	24666.35
RXL-3000	-0.13	107.25	5.00	40.00	21.85	0.00	0.00	1.00	0.96	11.00	7.33	136.43	56.00	-19683.61	-2124.62	448737.92	24506.37
RXL-3011	-0.21	107.25	5.00	38.00	21.20	0.00	0.00	1.00	0.96	11.00	7.33	135.78	54.00	-19709.36	-2131.42	448747.55	24518.33
RXL-3012	0.03	107.25	5.00	37.00	20.68	0.00	0.00	1.00	0.96	11.00	7.33	135.26	53.00	-19782.06	-2179.38	449133.54	24886.53
RXL-3013	-0.15	107.25	5.00	42.00	22.74	0.00	0.00	1.00	0.96	11.00	7.33	137.32	58.00	-19743.06	-2155.02	449559.53	25270.61
RXL-3014	0.05	107.25	5.00	38.00	21.39	0.00	0.00	1.00	0.96	11.00	7.33	135.97	54.00	-20044.90	-2426.09	448696.75	24454.34
RXL-3015	-0.21	107.25	5.00	42.00	23.39	0.00	0.00	1.00	0.96	11.00	7.33	137.97	58.00	-19881.04	-2199.95	446065.12	24763.55
RXL-3039	0.13	107.25	5.00	39.00	20.59	1.00	1.00	1.00	0.96	10.00	6.33	134.17	54.00	-19580.36	-2044.18	448551.60	24305.91
RXL-3017	0.29	107.25	5.00	37.00	19.68	1.00	1.00	1.00	0.96	8.00	4.33	131.26	50.00	-19361.84	-1809.73	446675.98	24034.88
RXL-3001	-0.37	82.25	4.00	36.00	19.70	0.00	0.00	1.00	0.96	9.00	5.33	107.28	49.00	-19320.91	-1833.98	448139.20	23983.01
RXL-3002	-0.50	107.25	5.00	40.00	21.84	0.00	0.00	1.00	0.96	11.00	7.33	136.42	56.00	-19669.71	-2103.41	448770.19	24616.97
RXL-3003	-0.49	91.22	4.00	40.00	21.85	0.00	0.00	0.00	0.00	11.00	7.33	120.40	55.00	-19965.35	-2181.99	451659.89	24430.88
RXL-3037	-0.19	107.25	5.00	38.00	20.78	0.00	0.00	1.00	0.96	11.00	7.33	135.36	54.00	-19640.35	-2072.96	448977.83	24794.34
RXL-3004	-0.23	107.25	5.00	40.00	21.86	0.00	0.00	1.00	0.96	11.00	7.33	136.44	56.00	-19484.31	-1963.01	448682.78	24479.52
RXL-3038	-0.46	107.25	5.00	35.00	19.08	0.00	0.00	1.00	0.96	8.00	4.33	130.66	48.00	-19057.30	-1582.13	447367.89	23212.33
RXL-3005	-0.21	107.25	5.00	38.00	21.10	0.00	0.00	1.00	0.96	11.00	7.33	135.68	54.00	-19619.94	-2100.90	448402.02	24214.83
RXL-3006	-0.22	82.25	4.00	40.00	21.85	0.00	0.00	1.00	0.96	11.00	7.33	111.43	55.00	-19599.64	-2059.70	448614.93	24417.82
RXL-3007	-0.47	89.47	4.00	40.00	21.85	0.00	0.00	1.00	0.96	11.00	7.33	118.65	55.00	-19818.85	-2282.63	447631.73	23470.48
RXL-3008	-0.28	107.25	5.00	35.00	19.27	0.00	0.00	1.00	0.96	11.00	7.33	133.85	51.00	-18648.69	-1239.62	446090.35	20045.25
RXL-3009	-0.35	107.25	5.00	22.00	12.15	0.00	0.00	1.00	0.96	5.00	5.00	124.40	32.00	-11550.67	-639.59	541535.24	24894.82
Res 1-101	0.18	107.25	5.00	42.00	21.10	0.00	0.00	1.00	0.96	11.00	7.33	135.68	58.00	-19588.14	-2015.27	448982.34	24479.27
RXL-3030	-0.06	82.25	4.00	38.00	18.93	0.00	0.00	1.00	0.96	9.00	5.33	106.51	51.00	-19386.08	-1864.40	448217.09	23778.32
RXL-3031	0.06	91.22	4.00	42.00	21.10	0.00	0.00	0.00	0.00	11.00	7.33	119.65	57.00	-19965.11	-2139.37	451769.03	24291.67
RXL-3032	0.56	107.25	5.00	41.00	20.09	0.00	0.00	1.00	0.96	11.00	7.33	134.67	57.00	-19572.36	-1999.83	449243.37	24622.86
RXL-3033	0.40	107.25	5.00	42.00	21.24	0.00	0.00	1.00	0.96	11.00	7.33	135.82	58.00	-19433.18	-1906.89	448847.07	24269.22
RXL-3028	-0.08	107.25	5.00	35.00	18.97	0.00	0.00	1.00	0.96	8.00	4.33	130.55	48.00	-18955.03	-1478.36	447520.04	23070.79
RXL-3034	0.46	107.25	5.00	41.00	20.45	0.00	0.00	1.00	0.96	11.00	7.33	135.03	57.00	-19586.95	-2044.17	448580.64	23969.94
RXL-3035	0.46	82.25	4.00	42.00	21.09	0.00	0.00	1.00	0.96	11.00	7.33	110.67	57.00	-19558.72	-1996.73	448949.44	24317.55
RXL-3036	0.28	89.47	4.00	42.00	21.09	0.00	0.00	1.00	0.96	11.00	7.33	117.89	57.00	-19617.74	-2083.68	447868.56	23309.28
RXL-3051	0.34	107.25	5.00	37.00	18.52	0.00	0.00	1.00	0.96	11.00	7.33	133.10	53.00	-18515.06	-1073.67	445758.17	19771.06
RXL-3052	0.24	107.25	5.00	25.00	11.45	0.00	0.00	1.00	0.96	5.00	5.00	123.70	35.00	-11283.47	-611.82	540427.19	24632.23

Supplementary Tables S13-S17. 6X18-based QSAR models.

**Reference**	**NOVA2 Binding Energy QSAR Model**						
Regression Analysis							
OVERALL FIT							
Multiple R	0.47614575		AIC	12.383259			
R Square	0.22671477		AICc	13.272148			
Adjusted F	0.20004977		SBC	15.251234			
Standard I	1.18359871						
Observati	31						
ANOVA				Alpha	0.05		
	df	*SS*	*MS*	*F*	*p-value*	*sig*	
Regressior	1	11.9109683	11.9109683	8.5023329	0.006776	yes	
Residual	29	40.6262711	1.4009059				
Total	30	52.5372393					
	coeff	*std err*	*t stat*	*p-value*	*lower*	*upper*	
Intercept	1.97254947	2.05217353	0.96120013	0.344398	-2.224617	6.16972	
BE	-0.00360133	0.00123508	-2.91587601	0.0067759	-0.006127	-0.00108	

	**QSAR 1**	**Interface Count QSAR Model**							
	Regression Analysis								
	OVERALL FIT								
	Multiple R	0.476439		AIC	12.37204				
	R Square	0.226995		AICc	13.26093				
	Adjusted F	0.200339		SBC	15.24002				
	Standard I	1.183385							
	Observati	31							
	ANOVA				Alpha	0.05			
		df	*SS*	*MS*	*F*	*p-value*	*sig*		
	Regressior	1	11.92567	11.92567	8.515905	0.006737	yes		
	Residual	29	40.61157	1.400399					
	Total	30	52.53724						
		coeff	*std err*	*t stat*	*p-value*	*lower*	*upper*		
	Intercept	2.998135	1.70139	1.762168	0.08858	-0.4816	6.477868		
	H Energy	0.242142	0.082976	2.918202	0.006737	0.072436	0.411848	1	

**QSAR 2**	**YASARA Quality QSAR Model**							
Regression Analysis								
OVERALL FIT								
Multiple R	0.669247		AIC	3.939284				
R Square	0.447891		AICc	5.477746				
Adjusted R Squa	0.408455		SBC	8.241246				
Standard Error	1.017811							
Observations	31							
ANOVA				Alpha	0.05			
	df	*SS*	*MS*	*F*	*p-value*	*sig*		
Regression	2	23.53096	11.76548	11.35731	0.000245	yes		
Residual	28	29.00628	1.035939					
Total	30	52.53724						
	coeff	*std err*	*t stat*	*p-value*	*lower*	*upper*	*vif*	
Intercept	-3.21818	3.792508	-0.84856	0.403321	-10.9868	4.550418		
bindPack1D|	-0.00139	0.000462	-3.005	0.005548	-0.00233	-0.00044	1.214649	
bindPack3D|	0.000355	0.00017	2.093294	0.045503	7.61E-06	0.000702	1.214649	

**QSAR 3 NOVA2 Terms QSAR Model**								
Regression Analysis								
OVERALL FIT								
Multiple R	0.671771		AIC	3.748629				
R Square	0.451276		AICc	5.28709				
Adjusted F	0.412082		SBC	8.05059				
Standard I	1.014686							
Observatio	31							
ANOVA				Alpha	0.05			
	df	*SS*	*MS*	*F*	*p-value*	*sig*		
Regressior	2	23.7088	11.8544	11.51374	0.000224	yes		
Residual	28	28.82844	1.029587					
Total	30	52.53724						
	coeff	*std err*	*t stat*	*p-value*	*lower*	*upper*	*vif*	
Intercept	-10.6578	4.214892	-2.5286	0.017369	-19.2916	-2.02397		
bindpot|	-0.00245	0.001132	-2.16644	0.038942	-0.00477	-0.00013	1.000444	
bindentro	-0.00853	0.001971	-4.32649	0.000174	-0.01256	-0.00449	1.000444	

**QSAR 4**	**Mixed QSAR Model**						
Regression Analysis							
OVERALL FIT							
Multiple R	0.737187		AIC	0.048134			
R Square	0.543445		AICc	2.448134			
Adjusted F	0.492717		SBC	5.784083			
Standard E	0.942536						
Observatio	31						
ANOVA				Alpha	0.05		
	df	*SS*	*MS*	*F*	*p-value*	*sig*	
Regression	3	28.55112	9.517039	10.71286	8.17E-05	yes	
Residual	27	23.98612	0.888375				
Total	30	52.53724					
	coeff	*std err*	*t stat*	*p-value*	*lower*	*upper*	*vif*
Intercept	-4.32749	3.542892	-1.22146	0.232472	-11.5969	2.941927	
bindpot|	-0.00251	0.001056	-2.37717	0.024792	-0.00468	-0.00034	1.008202
bindPack1	-0.00134	0.000428	-3.11965	0.004276	-0.00221	-0.00046	1.217811
bindPack3	0.000388	0.000158	2.463034	0.020441	6.48E-05	0.000712	1.224343

Supplementary Tables S18-S19. 5NX2-based QSAR model 3 potency-matched test set.

Peptide (train)	pEC50	Peptide (test)	pEC50	bindpot	bindsolvvdw	Predicted pEC50
Res 1-100	5.00	RXL-3000	5.00	-222.68	385.22	4.85
RXL-3011	5.55	RXL-3012	6.37	-244.31	401.09	5.91
RXL-3013	6.46	RXL-3014	6.89	-188.52	471.17	6.63
RXL-3015	6.98	RXL-3039	7.04	-274.55	438.04	7.84
RXL-3017	7.04	RXL-3001	7.67	-274.59	454.53	8.35
RXL-3002	7.80	RXL-3003	7.86	-252.19	475.99	8.43
RXL-3037	7.93	RXL-3004	7.97	-263.04	474.01	8.65
RXL-3038	8.04	RXL-3005	8.08	-256.17	465.7	8.22
RXL-3006	8.13	RXL-3007	8.16	-283.73	466.8	8.97
RXL-3008	8.31	RXL-3009	8.32	-266.35	451.13	8.03
Res 1-101	8.47	RXL-3030	8.58	-254.2	439.88	7.37
RXL-3031	8.64	RXL-3032	8.72	-263.21	468.53	8.49
RXL-3033	8.81	RXL-3028	8.95	-275.36	472.9	8.94
RXL-3034	9.23	RXL-3035	9.80	-277.51	473.5	9.01
RXL-3036	9.92	RXL-3051	9.92	-275.38	469.49	8.83
RXL-3052	10.00			-277.63	468.2	8.85
**Average Potency**	7.90		7.95			

**Regression Analysis**							
OVERALL FIT							
Multiple R	0.853747362		AIC	-4.936210129			
R Square	0.728884557		AICc	-1.299846493			
Adjusted R	0.687174489		SBC	-2.618443962			
Standard E	0.788252441						
Observatio	16						
ANOVA				Alpha	0.05		
	df	*SS*	*MS*	*F*	*p-value*	*sig*	
Regression	2	21.71593	10.85796653	17.47502678	0.000206777	yes	
Residual	13	8.077445	0.62134191				
Total	15	29.79338					
	coeff	*std err*	*t stat*	*p-value*	*lower*	*upper*	*vif*
Intercept	-12.87946315	3.605098	-3.572569808	0.003406004	-20.6678032	-5.0911231	
bindpot|	-0.025792961	0.008832	-2.920342157	0.011932662	-0.044873706	-0.00671222	1.12842368
bindsolvvd	0.030974114	0.008038	3.8536791	0.001993578	0.013610057	0.048338172	1.12842368

Supplementary Table S20. 5NX2-based QSAR model 3 potency rank-ordered test set.

QSAR 3	NOVA2 Terms QSAR Model							
**Peptide**	**Peptide Structure (N-Terminus, Amino Acid Sequence, C-Termi**	**pEC50**	**Molecules**	**pEC50**	**bindpot**	**bindsolvvdw**	**Pred pEC50 (80/20)**	**Pred pEC50 (60/40)**
Res 1-100	His-Aib-Glu-Gly-Thr-Phe-Thr-Ser-Asp-Bip-Bip	5.00	5nx2_ala_11_min	5.00	-222.68	385.22	6.00	5.88
RXL-3000	His-Aib-Glu-Gly-Thr-Phe(2-F)-Thr-Ser-Asp-Bip-Bip	5.00	5nx2_aib_10_min	5.00	-192.99	402.32	5.65	5.61
RXL-3011	His-Aib-Glu-Gly-Thr-Phe(2-Cl)-Thr-Ser-Asp-Bip-Bip	5.55	5nx2_ala_6_min	5.55	-244.31	401.09	6.71	6.49
RXL-3012	His-Aib-Glu-Gly-Thr-Phe(2-CF3)-Thr-Ser-Asp-Bip-Bip	6.37	5nx2_ala_10_min	6.37	-219.59	394.11	6.08	5.95
RXL-3013	His-Aib-Glu-Gly-Thr-Phe(2-CH3)-Thr-Ser-Asp-Bip-Bip	6.46	5nx2_ala_4_min	6.46	-188.52	471.17	6.66	6.55
RXL-3014	His-Aib-Glu-Gly-Thr-Phe(2-NO2)-Thr-Ser-Asp-Bip-Bip	6.89	5nx2_aib_8_min	6.89	-209.66	468.59	7.06	6.88
RXL-3015	His-Aib-Glu-Gly-Thr-Phe(2-CN)-Thr-Ser-Asp-Bip-Bip	6.98	5nx2_aib_6_min	6.98	-274.55	438.04	7.93	7.56
RXL-3039	His-Aib-Glu-Gly-Thr-Phe(2,6-F)-Thr-Ser-Asp-Bip-Bip	7.04	5nx2_aib_9_min	7.04	-264.65	466.44	8.18	7.80
RXL-3017	His-Aib-Glu-Gly-Thr-Phe(3,4,5-F)-Thr-Ser-Asp-Bip-Bip	7.04	5nx2_aib_7_min	7.04	-274.59	454.53	8.20	7.80
RXL-3001	H Ala-Aib-Glu-Gly-Thr-Phe(2-F)-Thr-Ser-Asp-Bip-Bip NH2	7.67	5nx2_ala_7_min	7.67	-270.36	455.50	8.13	7.74
RXL-3002	H His-Ala-Glu-Gly-Thr-Phe(2-F)-Thr-Ser-Asp-Bip-Bip NH2	7.80	5nx2_aib_4_min	7.80	-252.19	475.99	8.07	7.73
RXL-3003	H His-Aib-Ala-Gly-Thr-Phe(2-F)-Thr-Ser-Asp-Bip-Bip NH2	7.86	5nx2_ala_1_min	7.86	-260.53	427.75	7.48	7.16
RXL-3037	H His-Aib-Glu-Ala-Thr-Phe(2F)-Thr-Ser-Asp-Bip-Bip NH2	7.93	5nx2_ha_3_min	7.93	-263.04	474.01	8.27	7.88
RXL-3004	H His-Aib-Glu-Gly-Ala-Phe(2-F)-Thr-Ser-Asp-Bip-Bip NH2	7.97	5nx2_ha_8_min	7.97	-258.79	470.73	8.13	7.76
RXL-3038	H His-Aib-Glu-Gly-Thr-Ala-Thr-Ser-Asp-Bip-Bip NH2 2,80	8.04	5nx2_ha_6_min	8.04	-256.17	465.70	7.99	7.64
RXL-3005	H His-Aib-Glu-Gly-Thr-Phe(2-F)-Ala-Ser-Asp-Bip-Bip NH2	8.08	5nx2_aib_11_min	8.08	-228.64	398.37	6.34	6.17
RXL-3006	H His-Aib-Glu-Gly-Thr-Phe(2-F)-Thr-Ala-Asp-Bip-Bip NH2	8.13	5nx2_aib_5_min	8.13	-283.73	466.80	8.59	8.14
RXL-3007	H His-Aib-Glu-Gly-Thr-Phe(2-F)-Thr-Ser-Ala-Bip-Bip NH2	8.16	5nx2_ala_5_min	8.16	-277.26	461.16	8.36	7.94
RXL-3008	H His-Aib-Glu-Gly-Thr-Phe(2-F)-Thr-Ser-Asp-Ala-Bip NH2	8.31	5nx2_aib_3_min	8.31	-266.35	451.13	7.97	7.61
RXL-3009	H His-Aib-Glu-Gly-Thr-Phe(2-F)-Thr-Ser-Asp-Bip-Ala NH2	8.32	5nx2_ala_9_min	8.32	-258.37	466.39	8.05	**7.69**
Res 1-101	His-Aib-Glu-Gly-Thr-aMePhe(2-F)-Thr-Ser-Asp-Bip-Bip	8.47	5nx2_ala_3_min	8.47	-254.20	439.88	7.54	**7.23**
RXL-3030	Aib-Aib-Glu-Gly-Thr-aMePhe(2-F)-Thr-Ser-Asp-Bip-Bip	8.58	5nx2_aib_1_min	8.58	-275.36	447.32	8.10	**7.71**
RXL-3031	His-Aib-Aib-Gly-Thr-aMePhe(2-F)-Thr-Ser-Asp-Bip-Bip	8.64	5nx2_ha_0_min	8.64	-263.21	468.53	8.18	**7.81**
RXL-3032	His-Aib-Glu-Aib-Thr-aMePhe(2-F)-Thr-Ser-Asp-Bip-Bip	8.72	5nx2_ala_8_min	8.72	-275.06	466.66	8.40	**7.99**
RXL-3033	His-Aib-Glu-Gly-Aib-aMePhe(2-F)-Thr-Ser-Asp-Bip-Bip	8.81	5nx2_ha_4_min	8.81	-275.36	472.90	8.51	**8.08**
RXL-3028	His-Aib-Glu-Gly-Thr-Aib-Thr-Ser-Asp-Bip-Bip	8.95	5nx2_ha_5_mi	8.95	-275.54	471.74	**8.49**	**8.07**
RXL-3034	His-Aib-Glu-Gly-Thr-aMePhe(2-F)-Aib-Ser-Asp-Bip-Bip	9.23	5nx2_ha_2_min	9.23	-277.51	473.50	**8.56**	**8.13**
RXL-3035	His-Aib-Glu-Gly-Thr-aMePhe(2-F)-Thr-Aib-Asp-Bip-Bip	9.80	5nx2_ha_7_min	9.80	-277.86	468.77	**8.50**	**8.07**
RXL-3036	His-Aib-Glu-Gly-Thr-aMePhe(2-F)-Thr-Ser-Aib-Bip-Bip	9.92	5nx2_ha_1_min	9.92	-275.38	469.49	**8.45**	**8.03**
RXL-3051	His-Aib-Glu-Gly-Thr-aMePhe(2-F)-Thr-Ser-Asp-Aib-Bip	9.92	5nx2_ala_2_min	9.92	-267.06	458.66	**8.11**	**7.73**
RXL-3052	His-Aib-Glu-Gly-Thr-aMePhe(2-F)-Thr-Ser-Asp-Bip-Aib	10.00	5nx2_aib_2_min	10.00	-277.63	468.20	**8.48**	**8.05**
						**Avg pEC50 Train**	0.81	0.71
						**Avg pEC50 Test**	2.12	1.94

Supplementary Figures S9-S10. QSAR potency rank-ordered test set results.

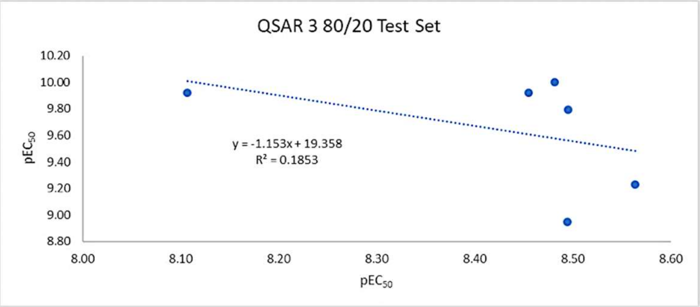

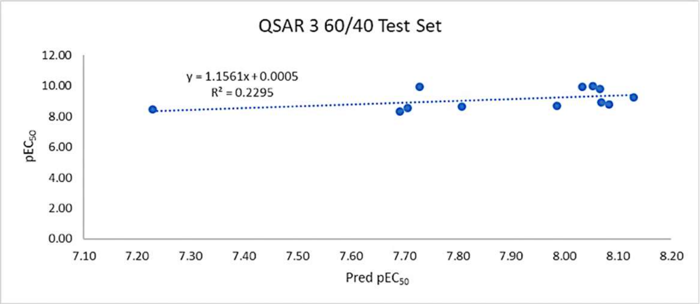

Supplement: Supplementary file 1 [file molecules-30-00012-s001.zip › molecules-2974778-supplementary.pdf]
